# Complete mitochondrial genomes of three fairy shrimps from snowmelt pools in Japan

**DOI:** 10.1186/s40850-022-00111-2

**Published:** 2022-02-09

**Authors:** Takashi Kitano, Hidenori Sato, Norihito Takahashi, Seiki Igarashi, Yushi Hatanaka, Keiji Igarashi, Kazuo Umetsu

**Affiliations:** 1 Graduate School of Science and Engineering, Ibaraki University, 4-12-1 Nakanarusawa, Hitachi, 316-8511 Japan; 2 Yamagata University School of Medicine, 2-2-2 Iidanishi, Yamagata, 990-9585 Japan; 3 Kukuma System Design, 524–15 Minehama, Rausu, 086–1751 Japan; 4 Research Institute of Energy, Environment and Geology, Hokkaido Research Organization, Kita 19, Nishi 12, Kita-Ku, Sapporo, 060-0819 Japan; 5 Yamagata Prefectural Natural Park, 19-1 Fukura, Yuza, 999-8521 Japan; 6 Sakata UNESCO Association, 2-59, Chuonishimachi, Sakata, 998-0034 Japan

**Keywords:** Branchiopoda, Anostraca, Mitochondrion, Phylogeny, GC content

## Abstract

**Background:**

Fairy shrimps belong to order Anostraca, class Branchiopoda, subphylum Crustacea, and phylum Arthropoda. Three fairy shrimp species (*Eubranchipus uchidai*, *E. asanumai*, and *E. hatanakai*) that inhabit snowmelt pools are currently known in Japan. Whole mitochondrial genomes are useful genetic information for conducting phylogenetic analyses. Mitochondrial genome sequences for Branchiopoda members are gradually being collated.

**Results:**

Six whole mitochondrial genomes from the three *Eubranchipus* species are presented here. *Eubranchipus* species share the anostracan pattern of gene arrangement in their mitochondrial genomes. The mitochondrial genomes of the *Eubranchipus* species have a higher GC content than those of other anostracans. Accelerated substitution rates in the lineage of *Eubranchipus* species were observed.

**Conclusion:**

This study is the first to obtain whole mitochondrial genomes for Far Eastern *Eubranchipus* species. We show that the nucleotide sequences of cytochrome oxidase subunit I and the 16S ribosomal RNA of *E. asanumai* presented in a previous study were nuclear mitochondrial DNA segments. Higher GC contents and accelerated substitution rates are specific characteristics of the mitochondrial genomes of Far Eastern *Eubranchipus*. The results will be useful for further investigations of the evolution of Anostraca as well as Branchiopoda.

**Supplementary Information:**

The online version contains supplementary material available at 10.1186/s40850-022-00111-2.

## Background

The fairy shrimp genus *Eubranchipus* belongs to family Chirocephalidae, order Anostraca, class Branchiopoda, subphylum Crustacea, and phylum Arthropoda. These shrimps occur in temporary pools formed by snowmelt in the forest groves in northern Japan [1]. They hatch from resting eggs when the snowmelt water appears in early spring and mature in the pool and lay eggs just before the water dries up in late spring. Thus far, studies of these shrimps have been limited [1–4], because they are found only in inconspicuous locations such as forest bushes and only during a short period [5]. In 2018, Takahashi et al. [1] described three new species of *Eubranchipus* for the first time in 62 years, from Far East Asia. They also reviewed the morphological ambiguity of the earlier description of *E. uchidai* and updated the molecular systematics with the new Asian taxa.

The animal mitochondrial genome is a small, extrachromosomal genome. It has a simple conserved structure that is approximately 16 kb long, with 13 protein-coding genes (*COX1*, *COX2*, *ATP8*, *ATP6*, *COX3*, *ND3*, *ND5*, *ND4*, *ND4L*, *ND6*, *CYTB*, *ND1*, and *ND2*), two ribosomal RNA genes (*16S* and *12S*), 22 tRNA genes, and a noncoding region known as a control region [6]. Utilization of the complete mitochondrial genome for molecular phylogenetic analysis has two main advantages: one is that more phylogenetic information can be obtained than when using partial sequences, and the other is that NUMTs are avoided. In this study, we determined the complete mitochondrial genomes of three *Eubranchipus* species in Japan and compared these with those of other anostracans.

Although the animal mitochondrial genome is conserved across the animal kingdom, rearrangements in gene order may occur. In the Branchiopoda, three gene order patterns have been reported [7, 8]. The first is the “ancestral pancrustacean pattern” [8], which is shared by *Daphnia* and *Triops* species. The second, based on the ancestral pancrustacean pattern, is the “anostracan pattern” [7], which translocates (*trnI* + *trnQ*) from between the control region and *trnM* to between *trnW* and *trnC*, together with an inversion of *trnI*. *Artemia*, *Phallocryptus*, and *Streptocephalus* species share this pattern. The third pattern is observed in *Branchinella kugenumaensis*, which underwent a further large inversion of the block (*trnM* + *ND2* + *trnW* + *trnI*) from the anostracan pattern [7].

GC content differences have been observed in branchiopod mitochondrial genomes [9]. Anostraca (36.8%) and Onychocaudata (*Limnadia lenticularis* + Cladocera) (35.0%) have a significantly higher GC content than Notostraca (30.5%), which is explained by the preferential AT to GC substitution bias during the evolution of the Anostraca and Onychocaudata lineages.

In this study, we report on the sequencing and analysis of six mitochondrial genomes from three *Eubranchipus* species of Chirocephalidae, Anostraca. The newly obtained mitochondrial genomes were analyzed alongside all currently available mitochondrial genomes of Branchiopoda members to gain insight into the evolution of this class of crustaceans.

## Results

### Complete mitochondrial genomes

**Fig. 1:**
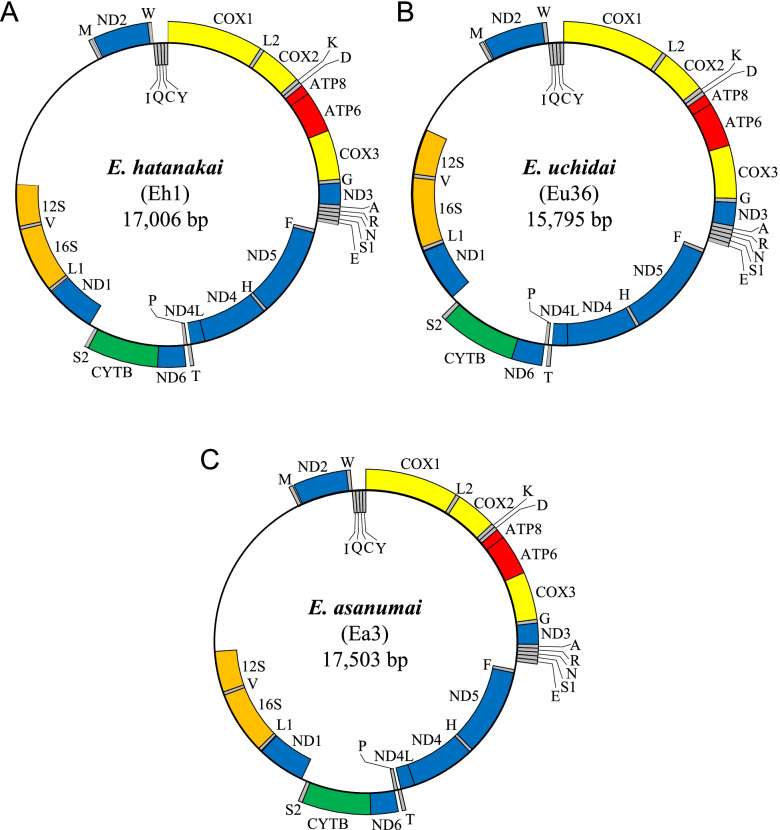
Schematic structures of the mitochondrial genomes of *E. hatanakai* (Eh1) (**A**), *E. uchidai* (Eu36) (**B**), and *E. asanumai* (Ea3) (**C**). Yellow: cytochrome c oxidase, blue: NADH dehydrogenase, red: ATP synthase, green: cytochrome b, orange: ribosomal RNA, gray: transfer RNA

We used two individuals of each species (*Eubranchipus hatanakai* Takahashi & Hamasaki, in Takahashi et al., 2018 [Sample IDs: Eh1, Eh6], *Eubranchipus uchidai* (Kikuchi, 1957) [Sample IDs: Eu17, Eu36], and *Eubranchipus asanumai* Takahashi, in Takahashi et al., 2018 [Sample IDs: Ea3, Ea4]), to identify the complete mitochondrial genomes. The estimated respective DINs and concentrations of the extracted genomic DNAs were as follows: Eh1 (7.4, 39.7 ng/μL), Eh6 (7.3, 57.8 ng/μL), Eu17 (7.6, 53.5 ng/μL), Eu36 (7.8, 59.1 ng/μL), Ea3 (7.8, 24.2 ng/μL), and Ea4 (7.5, 34.2 ng/μL). In total, 4.4–7.3 Gb of corrected data were obtained from 5.0–7.8 Gb of raw data (i.e., 90.2–93.7%) for each fastq dataset using the Pollux 1.0.2 program [10] (Additional file 1: Table S1). The estimated k-mers are shown in Additional file 1: Table S1. De novo genome sequence assembly was performed using the Ray 2.1.0 program [11], and 329,762–491,652 assembled sequences were obtained. Partial mitochondrial DNA sequences were obtained from these assembled sequences via tBLASTn searches [12] ([Eh1]: scaffold-325,202 (7647 bp) containing *ND2*-*COX1*-*COX2*-*ATP8*-*ATP6*-*COX3*-*ND3*, scaffold-21 (4137 bp) containing *ND5*-*ND4*-*ND4L*-*ND6*-*CYTB*, scaffold-42 (3350 bp) containing ND1; [Eh6]: scaffold-0 (7649 bp) containing *ND2*-*COX1*-*COX2*-*ATP8*-*ATP6*-*COX3*-*ND3*, scaffold-328,332 (8201 bp) containing *ND5*-*ND4*-*ND4L*-*ND6*-*CYTB*-*ND1*; [Eu17]: scaffold-96 (3279 bp) containing *ND2*-*COX1*, scaffold-31 (3483 bp) containing *COX2*-*ATP8*-*ATP6*-*COX3*-*ND3*, scaffold-12 (7853 bp) containing *ND5*-*ND4*-*ND4L*-*ND6*-*CYTB*-*ND1*; [Eu36]: scaffold-34 (6444 bp) containing *ND2*-*COX1*-*COX2*-*ATP8*-*ATP6*-*COX3*-*ND3*, scaffold-12 (7889 bp) containing *ND5*-*ND4*-*ND4L*-*ND6*-*CYTB*-*ND1*; [Ea3]: scaffold-157 (5954 bp) containing *ND2*-*COX1*-*COX2*-*ATP8*-*ATP6*-*COX3*-*ND3*, scaffold-12 (8281 bp) containing *ND5*-*ND4*-*ND4L*-*ND6*-*CYTB*-*ND1*; [Ea4]: scaffold-403,293 (6440 bp) containing *ND2*-*COX1*-*COX2*-*ATP8*-*ATP6*-*COX3*-*ND3*, scaffold-4 (8198 bp) containing *ND5*-*ND4*-*ND4L*-*ND6*-*CYTB*-*ND1*) (Additional file 2: Selected scaffold sequences). We then performed mitochondrial genome sequence assembly in the NOVOPlasty 3.2 program [13] using the longest mitochondrial DNA sequence obtained as the seed for each dataset (Eh6: scaffold-328,332, Eu17: scaffold-12, Eu36: scaffold-12, Ea3: scaffold-12, Ea4: scaffold-4). Because the complete mitochondrial genome sequence of Eh1 could not be obtained using scaffold-325,202 as the seed in the NOVOPlasty assembly, we used a combined sequence comprising two scaffolds (scaffold-325,202 and scaffold-21) as the seed. Circularized assembly sequences were obtained for all samples. Next, we remapped the fastq reads to the assembled mitochondrial genome sequence to check the sequence read depth (Additional file 3: Fig. S1). We thus obtained complete mitochondrial genomes for *E. hatanakai* (Eh1 [17,006 bp], Eh6 [17,006 bp]), *E. uchidai* (Eu17 [15,795 bp], Eu36 [15,795 bp]), and *E. asanumai* (Ea3 [17,503 bp], Ea4 [17,503 bp]) (Fig. 1). We found three variant sites (172AG, 9010 AC, and 14234CT) in Eh1, one (4804AG) in Eh6, and one (13191AT) in Eu17. Lists of the annotated loci of the six sequences are given in Additional file 4: Table S2.

These *Eubranchipus* species share the anostracan gene arrangement pattern in their mitochondrial genomes [7]. The nucleotide sequence data obtained in this study were deposited in the DDBJ/EMBL/GenBank International Nucleotide Sequence Database (accession numbers: LC633437–LC633442).

### *COX1* and *16S* sequences

**Fig. 2:**
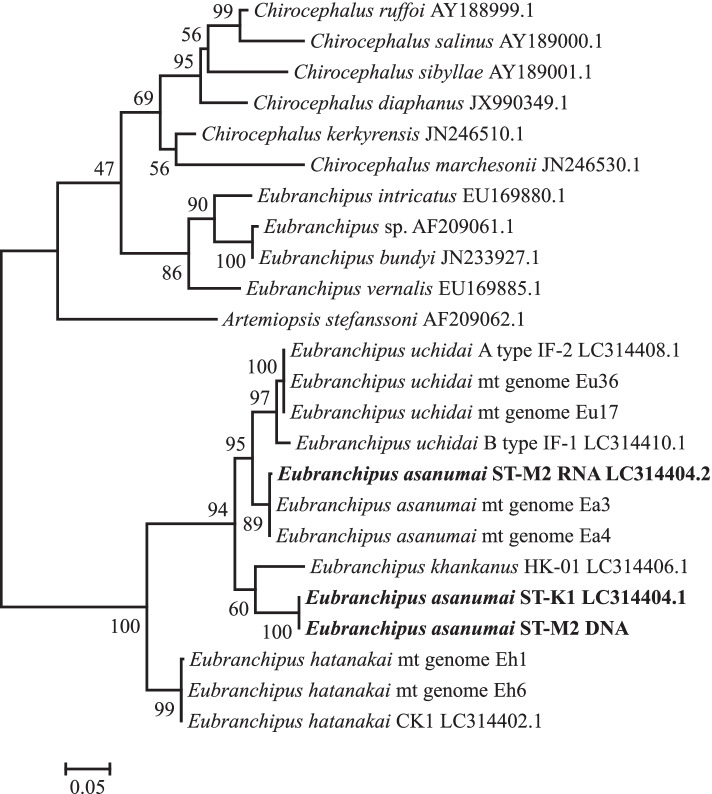
Phylogenetic tree of Chirocephalidae species based on *COX1* nucleotide sequencing data. The tree was constructed using the maximum likelihood method and the GTR + γ model. The tree was rooted using midpoint rooting. The scale bar represents the number of nucleotide substitutions per site. Bootstrap values (percentages of 500 replicates) are shown at the nodes. *E. asanumai* sequences from individuals ST-K1 and ST-M2 are shown in bold

**Fig. 3:**
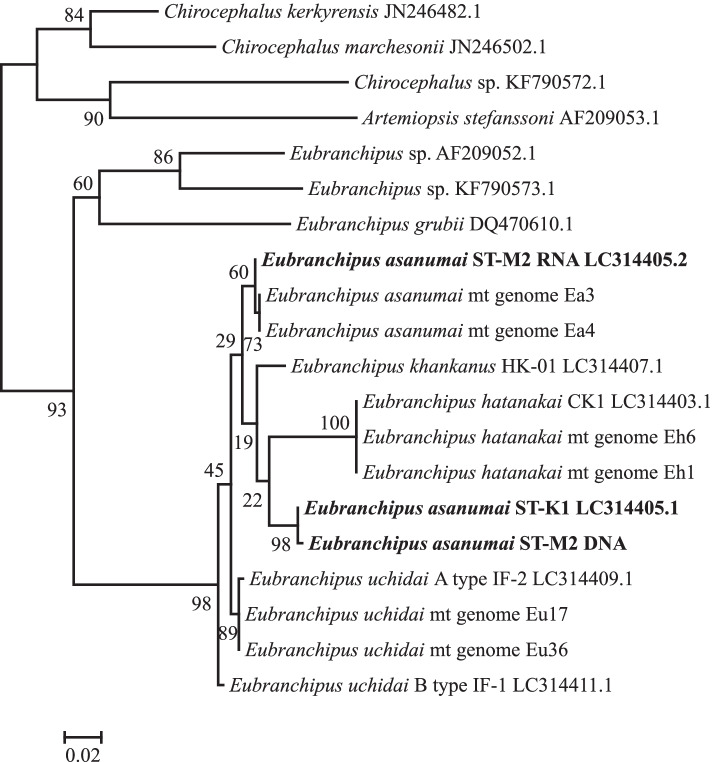
Phylogenetic tree of Chirocephalidae species based on *16S* nucleotide sequencing data. The tree was constructed using the maximum likelihood method and the GTR + γ model. The tree was rooted using midpoint rooting. The scale bar represents the number of nucleotide substitutions per site. Bootstrap values (percentages of 500 replicates) are shown at the nodes. *E. asanumai* sequences from individuals ST-K1 and ST-M2 are shown in bold

We constructed phylogenetic trees for the *COX1* (Fig. 2) and *16S* (Fig. 3) sequences of Chirocephalidae species. For *E. uchidai*, the *COX1* sequences of Eu17 and Eu36 were identical to that of the A type of *E. uchidai* in the DNA databank (LC314408.1, [1]), but we observed one nucleotide difference in the *16S* sequence (LC314409.1, [1]). For *E. hatanakai*, the *COX1* sequence of Eh6 was identical to that of *E. hatanakai* in the DNA databank (LC314402.1, [1]), while there was one nucleotide difference in that of Eh1. The *16S* sequences of both Eh1 and Eh6, however, were identical to that of *E. hatanakai* in the DNA databank (LC314403.1, [1]).

We observed distinct differences for *E. asanumai*, however: there were 44 nucleotide differences (0.090 differences per site) between the *COX1* sequences of Ea3 and Ea4 and that of *E. asanumai* in the DNA databank (LC314404.1, [1]). In addition, there were 13 nucleotide differences (0.027 differences per site) between the *16S* sequences of Ea3 and Ea4 and that of *E. asanumai* in the DNA databank (LC314405.1, [1]).

We extracted genomic DNA and total RNA samples from one individual of *E. asanumai* (ST-M2) and conducted PCR and RT-PCR analyses on them using the same primer sets. The *COX1* sequence amplified from the genomic DNA sample was identical to that of the *E. asanumai* sequence in the DNA databank (LC314404.1, [1]) (Fig. 2), and the *16S* sequence had only one nucleotide different from that of the *E. asanumai* sequence in the DNA databank (LC314405.1, [1]) (Fig. 3). In contrast, both the *COX1* sequence and the *16S* sequence amplified from the total RNA sample had only one nucleotide different from Ea3 and Ea4 (Figs. 2 and 3).

### Nucleotide differences in mitochondrial DNA within and between *Eubranchipus* species

We observed four nucleotide differences between the two mitochondrial DNA sequences (Eh1 and Eh6) of *E. hatanakai*: 172AG nonsynonymous in *COX1*, 3368CG nonsynonymous in *COX3*, 7766AG synonymous in *ND4*, and 8628AG nonsynonymous in *ND6*. We also observed four nucleotide differences between the two mitochondrial DNA sequences (Eu17 and Eu36) of *E. uchidai*: 2675AG nonsynonymous in *ATP6*, 3150CT nonsynonymous in *ATP6*, 8010AG nonsynonymous in *ND4L*, and 13,703 AC in the region between *rrnS* and *trnM* (probably the D-loop region). However, we found 20 nucleotide differences between the two mitochondrial DNA sequences (Ea3 and Ea4) of *E. asanumai*, 10 of which were as follows: 1727AG synonymous in *COX2*, 2959CT synonymous in *ATP6*, 3243GA synonymous in *COX3*, 3324AG synonymous in *COX3*, 5484TC synonymous in *ND5*, 6989TC nonsynonymous in *ND4*, 7849AG synonymous in *ND4L*, 7923AG synonymous in *ND4L*, 8199CT in *trnP*, and 16998AG synonymous in *ND2*, and the remaining 10 were 10542AG, 10974CT, 12940AG, 13547CT, 13793TA, 14906TC, 15843TG, 15899TC, 15957CT, and 16195GA in the region between *rrnS* and *trnM* (probably the D-loop region). There were no insertion or deletion differences between the two sequences of each species.

The genetic distances for each locus among the *Eubranchipus* species are listed in Additional file 5: Table S3. The overall genetic distance between *E. hatanakai* and *E. uchidai* was 0.192, ranging from 0.033 for *trnH* to 0.340 for *ATP8*. The overall genetic distance between *E. hatanakai* and *E. asanumai* was 0.189, ranging from 0.033 for *trnL1* to 0.331 for *ATP8*. In addition, the overall genetic distance between *E. uchidai* and *E. asanumai* was 0.041, ranging from 0 for 10 tRNA loci (*trnL2*, *trnK*, *trnD*, *trnG*, *trnR*, *trnS1*, *trnH*, *trnT*, *trnI*, and *trnC*) to 0.070 for *trnV*. The overall genetic distances between *E. hatanakai* and *E. grubii*, between *E. uchidai* and *E. grubii*, and between *E. asanumai* and *E. grubii* were 0.269, 0.260, and 0.260, respectively.

### GC content of mitochondrial genomes of Branchiopoda species

We estimated the GC contents of the whole mitochondrial genomes, 13 protein-coding genes with first, second, and third codon positions, and two rRNA regions for Branchiopoda (Tables 1 and 2). The GC content of the whole mitochondrial genomes of the Anostraca, Artemiidae, ranged from 35.49% for *Artemia sinica* to 37.55% for *A. urmiana*; that of the Chirocephalidae ranged from 33.04% in *E. grubii* to 38.96% in *E. hatanakai*; and that of the Thamnocephalidae and Streptocephalidae ranged from 31.83% in *B. kugenumaensis* (Japan) to 35.40% in *Streptocephalus sirindhornae*. These were significantly different among taxa (Kruskal–Wallis, *p* < 0.05). Multiple comparisons indicated that Artemiidae had a significantly higher GC content than the Thamnocephalidae and Streptocephalidae (Steel–Dwass, *p* < 0.05), but other comparisons did not indicate significant differences. We obtained similar results for the third codon position and two rRNAs. Multiple comparisons of the third codon position (Steel–Dwass, *p* < 0.05) and the two rRNAs (Steel–Dwass, *p* < 0.05) also indicated that the Artemiidae had a significantly higher GC content than the Thamnocephalidae and Streptocephalidae, while other comparisons did not indicate any significant differences.

**Table 1:** Mitochondrial genome data used in the study

Order	Family	Species	Accession number	Reference
Anostraca	Chirocephalidae	*Eubranchipus hatanakai* Eh1	LC633439.1	This study
Anostraca	Chirocephalidae	*Eubranchipus hatanakai* Eh6	LC633440.1	This study
Anostraca	Chirocephalidae	*Eubranchipus uchidai* Eu17	LC633441.1	This study
Anostraca	Chirocephalidae	*Eubranchipus uchidai* Eu36	LC633442.1	This study
Anostraca	Chirocephalidae	*Eubranchipus asanumai* Ea3	LC633437.1	This study
Anostraca	Chirocephalidae	*Eubranchipus asanumai* Ea4	LC633438.1	This study
Anostraca	Chirocephalidae	*Eubranchipus grubii*	MT410793.1	No reference
Anostraca	Thamnocephalidae	*Branchinella kugenumaensis* Japan	MW136376.1	No reference
Anostraca	Thamnocephalidae	*Branchinella kugenumaensis* China	MN660045.1	[7]
Anostraca	Thamnocephalidae	*Phallocryptus tserensodnomi*	KP273592.1	[14]
Anostraca	Streptocephalidae	*Streptocephalus cafer*	MN720104.1	[15]
Anostraca	Streptocephalidae	*Streptocephalus sirindhornae*	KP273593.1	[16]
Anostraca	Artemiidae	*Artemia sinica*	MK069595.1	[17]
Anostraca	Artemiidae	*Artemia urmiana*	JQ975176.1	[18]
Anostraca	Artemiidae	*Artemia tibetiana*	JQ975178.1	[18]
Anostraca	Artemiidae	*Artemia franciscana*	X69067.1	[19]
Diplostraca	Daphniidae	*Daphnia laevis*	MK059395.1	[20]
Diplostraca	Daphniidae	*Daphnia similis*	MH688061.1	[21]
Diplostraca	Daphniidae	*Daphnia galeata*	LC152879.1	[22]
Diplostraca	Daphniidae	*Daphnia pulex*	AF117817.1	[23]
Diplostraca	Daphniidae	*Daphnia magna*	MT199637.1	[24]
Diplostraca	Sididae	*Diaphanosoma dubium*	MG428405.1	[25]
Diplostraca	Limnadiidae	*Limnadia lenticularis*	MH618637.1	[26]
Diplostraca	Limnadiidae	*Gondwanalimnadia* sp. MT-2020	MN625703.1	[27]
Notostraca	Triopsidae	*Triops granarius*	MF496656.1	[28]
Notostraca	Triopsidae	*Triops cancriformis*	AB084514.1	[29]
Notostraca	Triopsidae	*Triops australiensis*	LK391946.1	[30]
Notostraca	Triopsidae	*Triops longicaudatus*	AY639934.1	[8]
Notostraca	Triopsidae	*Lepidurus apus lubbocki*	MK579381.1	[9]
Notostraca	Triopsidae	*Lepidurus arcticus*	MK579380.1	[9]

**Table 2:** GC content of mitochondrial genomes of Branchiopoda

			GC content					
Order	Family	Species	Total	13PCGs	1st	2nd	3rd	2rRNAs
Anostraca	Chirocephalidae	*Eubranchipus hatanakai* Eh1	38.96	39.47	45.41	38.22	34.75	36.38
Anostraca	Chirocephalidae	*Eubranchipus uchidai* Eu36	38.57	39.30	45.72	38.11	34.06	35.68
Anostraca	Chirocephalidae	*Eubranchipus asanumai* Ea3	38.71	39.22	45.63	37.97	34.03	36.27
Anostraca	Chirocephalidae	*Eubranchipus grubii*	33.04	33.73	42.13	37.09	21.93	32.38
Anostraca	Thamnocephalidae	*Branchinella kugenumaensis* Japan	31.83	32.27	39.62	35.59	21.58	30.24
Anostraca	Thamnocephalidae	*Branchinella kugenumaensis* China	32.22	32.46	39.72	35.98	21.66	29.91
Anostraca	Thamnocephalidae	*Phallocryptus tserensodnomi*	34.58	36.15	42.22	36.83	29.39	33.19
Anostraca	Streptocephalidae	*Streptocephalus cafer*	31.84	32.00	40.49	36.06	19.45	27.94
Anostraca	Streptocephalidae	*Streptocephalus sirindhornae*	35.40	35.57	42.11	36.47	28.12	33.16
Anostraca	Artemiidae	*Artemia sinica*	35.49	35.97	41.22	36.52	30.14	36.88
Anostraca	Artemiidae	*Artemia urmiana*	37.55	38.19	43.21	36.72	34.65	37.50
Anostraca	Artemiidae	*Artemia tibetiana*	37.30	37.92	43.02	36.96	33.78	38.17
Anostraca	Artemiidae	*Artemia franciscana*	35.56	36.05	41.15	36.40	30.59	37.00
Diplostraca	Daphniidae	*Daphnia laevis*	31.39	32.60	40.18	36.69	20.90	27.86
Diplostraca	Daphniidae	*Daphnia similis*	29.63	30.61	36.89	34.93	20.00	27.00
Diplostraca	Daphniidae	*Daphnia galeata*	36.22	38.22	44.42	37.73	32.51	31.24
Diplostraca	Daphniidae	*Daphnia pulex*	37.74	39.57	44.56	37.40	36.75	32.17
Diplostraca	Daphniidae	*Daphnia magna*	32.42	34.06	40.09	35.63	26.46	29.24
Diplostraca	Sididae	*Diaphanosoma dubium*	34.32	34.57	41.07	36.79	25.83	31.87
Diplostraca	Limnadiidae	*Limnadia lenticularis*	34.96	36.04	41.21	36.25	30.66	31.28
Diplostraca	Limnadiidae	*Gondwanalimnadia* sp. MT-2020	33.52	34.14	39.42	36.08	26.92	29.91
Notostraca	Triopsidae	*Triops granarius*	29.77	30.44	35.65	35.40	20.25	27.03
Notostraca	Triopsidae	*Triops cancriformis*	31.21	31.68	38.00	35.59	21.43	28.95
Notostraca	Triopsidae	*Triops australiensis*	28.46	28.77	36.18	35.37	14.76	27.11
Notostraca	Triopsidae	*Triops longicaudatus*	30.71	31.42	37.78	35.97	20.49	28.12
Notostraca	Triopsidae	*Lepidurus apus lubbocki*	27.84	28.99	34.46	35.10	17.41	26.53
Notostraca	Triopsidae	*Lepidurus arcticus*	32.47	33.87	39.13	36.53	25.94	27.87

*13PCGs* 13 protein-coding genes, *1st* first codon position, *2nd* second codon position, *3rd* third codon position, *2rRNA* two rRNA genes

The GC content of the second codon position of the Artemiidae ranged from 36.40% in *A. franciscana* to 36.96% in *A. tibetiana*; that of the Chirocephalidae ranged from 37.09% in *E. grubii* to 38.22% in *E. hatanakai*; and that of the Thamnocephalidae and Streptocephalidae ranged from 35.59% in *B. kugenumaensis* (Japan) to 36.83% in *Phallocryptus tserensodnomi*, and these values differed significantly among taxa (Kruskal–Wallis, *p* < 0.05). Multiple comparisons indicated that the Chirocephalidae had a significantly higher GC content than the Thamnocephalidae and Streptocephalidae (Steel–Dwass, *p* < 0.05), while other comparisons did not indicate any significant differences.

### Phylogenetic tree of Branchiopoda species

In addition to our three mitochondrial genome sequences from *E. hatanakai* (Eh1), *E. uchidai* (Eu36), and *E. asanumai* (Ea3), we used mitochondrial DNA sequences from ten other Anostraca species, six Notostraca species, and eight Diplostraca species (Table 1). Selected substitution models are listed in Additional file 6: Table S4. For the ML approach [31], we used the models selected by AIC [32]. The corrected AIC (AICc) [33] selected the same models as were selected by AIC. For the BS approach [34], we used the models selected by the BIC [35]. For the NJ method [36], we selected the Tamura and Nei [37] model with a γ correction (α = 0.41) as the best available model.

**Fig. 4:**
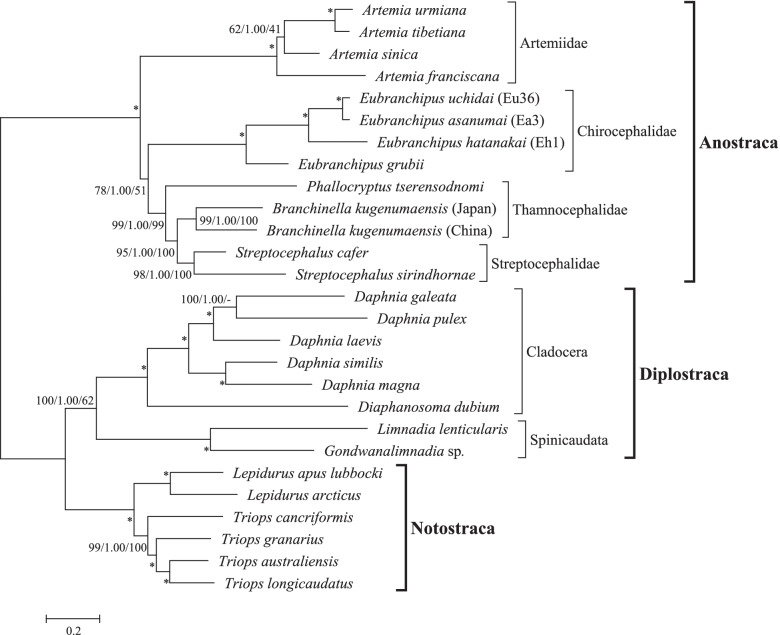
A phylogenetic tree of Branchiopoda species based on the 13 concatenated protein-coding genes in the mitochondrial genome. The tree was constructed using the neighbor-joining method. The scale bar represents the number of nucleotide substitutions per site. Values near the nodes are the percent bootstrap probability of the neighbor-joining method, Bayesian posterior probability, and percent bootstrap probability of the maximum likelihood method (NJ/BS/ML). Support of 100/1.00/100 is indicated by an asterisk

We constructed the NJ tree of Branchiopoda species using the 13 protein-coding mitochondrial genes (Fig. 4). The divergent orders in Anostraca were Artemiidae, Chirocephalidae, Thamnocephalidae and Streptocephalidae. *P. tserensodnomi* and *B. kugenumaensis* (Thamnocephalidae) did not form a monophyletic cluster, whereas Cladocera and Spinicaudata (Diplostraca) did form a cluster. Two *B. kugenumaensis* mitochondrial genome sequences had been deposited in the DNA database, one from Japan and the other from China, and there are large nucleotide differences between them.

We also reconstructed the BS tree (Additional file 7: Fig. S2) and the ML tree (Additional file 8: Fig. S3). The topologies of the NJ (Fig. 4) and BS (Additional file 7: Fig. S2) trees were identical, but there was one difference between the topology of the ML tree and those of the NJ and BS trees: *Daphnia galeata* and *D. laevis* formed a cluster with a 64% bootstrap value in the ML tree (Additional file 8: Fig. S3). We compared the two topologies (NJ and BS versus ML) using the likelihood method (Table 3). The likelihood of the topology of the ML tree was higher than that of the NJ and BS trees, but according to three tests, this difference was not statistically significant.

**Table 3:** Comparisons of two topologies of the 13 protein-coding genes in the mitochondrial genomes of Branchiopoda

Topology	li	Dli	± SE	pKH	pSH	pRELL
ML	− 180,106.145	0.000	0.000	N/A	N/A	0.669
NJ/BS	−180,112.298	−6.153	14.654	0.337	0.326	0.331

li: log-likelihood value

Dli: difference in the log-likelihood value

± SE: standard error

pKH: *p*-value for the Kishino and Hasegawa normal test [38]

pSH: *p*-value with multiple-comparison correction (MC in Table 1 of [39])

pRELL: Resampling estimated log-likelihood (RELL) bootstrap proportions [38]

In all three phylogenetic trees (Fig. 4, Additional file 7: Fig. S2, and Additional file 8: Fig. S3), the branches of the Diplostraca, Artemiidae, and Chirocephalidae clusters appeared longer than those of the Notostraca, Thamnocephalidae, and Streptocephalidae clusters. We investigated the constancy of the substitution rate and the variation among lineages (Table 4, Additional file 9: Fig. S4). The “no clock” model means that substitution rates are entirely free to vary from branch to branch, whereas the “global clock” model means that all branches have the same substitution rate. There was a significant difference between the no clock and global clock models, suggesting variations in the substitution rates among lineages. The “local clock1” model assumes that the Artemiidae, Chirocephalidae, and Diplostraca lineages have higher substitution rates than those of the Notostraca, Thamnocephalidae, and Streptocephalidae. There was a significant difference between the global clock and local clock1 models. In the “local clock2” model, the substitution rates of the Artemiidae, Chirocephalidae, and Diplostraca were assumed to be independent, and this was also significantly different from the global clock model. This implies that the acceleration of the substitution rate occurred independently in the Artemiidae, Chirocephalidae, and Diplostraca lineages.

**Table 4:** Substitution rate comparisons of the phylogenetic tree of the 13 protein-coding genes of mitochondrial genomes of Branchiopoda

Models	LR	d.f.	*p*
No clock vs. Global clock	336.07	25	3.24 × 10^−56^
Global clock vs. Local clock1	129.64	1	4.97 × 10^−30^
Global clock vs. Local clock2	154.46	3	2.87 × 10^−33^

No clock: substitution rates are entirely free to vary from branch to branch (lnL: − 180,112.2984, 51 parameters)

Global clock: all branches have the same substitution rate (lnL: − 180,280.3318, 26 parameters)

Local clock1: Artemiidae (#1), Chirocephalidae (#2), and Diplostraca (#3) have higher substitution rates than Notostraca, Thamnocephalidae, and Streptocephalidae, and the three ratios (#1 = #2 = #3) are equal (lnL: − 180,215.5223, 27 parameters)

Local clock2: Artemiidae (#1), Chirocephalidae (#2), and Diplostraca (#3) have higher substitution rates than Notostraca, Thamnocephalidae, and Streptocephalidae, and the three ratios (#1, #2, and #3) are independent (lnL: − 180,203.1015, 29 parameters)

*LR* the likelihood scores of the two models

*d.f.* degrees of freedom

*p*: *p*-value from the χ^2^ test

We estimated the overall ratios of nonsynonymous substitutions per nonsynonymous site to synonymous substitutions per synonymous site (ω) to be 0.109, 0.106, 0.121, 0.166, and 0.138 for the Artemiidae, Chirocephalidae, Thamnocephalidae and Streptocephalidae, Diplostraca, and Notostraca clusters, respectively.

## Discussion

### Nuclear mitochondrial DNA segments in previous annotations of *Eubranchipus asanumai*

After the study conducted by Takahashi et al. [1] was published, different types of sequence of both *COX1* and *16S* were obtained from different *E. asanumai* individuals based on PCR and sequencing using genomic DNA. We initially considered the possibility of the presence of another species in the Shiretoko area. However, based on morphological observations, this was not possible [1]. In this study, therefore, we determined the complete mitochondrial genome. This is thus the first study to reveal the whole mitochondrial genomes of Far Eastern *Eubranchipus* species. At the same time, we performed RT-PCR using extracted total RNA. Because the sequences amplified using this technique would be functional, these sequences could not be NUMTs. It is regrettable that (for unknown reasons) the *COX1* and *16S* sequences of *E. asanumai* presented by Takahashi et al. [1] were incorrect. In addition, separate from the complete mitochondrial genome sequences of *E. asanumai*, we observed a 244 bp fragment from the Ea3 Ray genome assembly data (scaffold-278,847) and a 387 bp fragment from the Ea4 Ray genome assembly data (scaffold-236,301) (Additional file 2: Selected scaffold sequences). They were almost identical (one nucleotide difference) to the sequence of LC314404.1 (NUMT of *COXI*), which was published by Takahashi et al. We also identified a 139 bp fragment from the Ea3 Ray genome assembly data (scaffold-458,031), which was almost identical (one nucleotide difference) to the sequence of LC314405.1 (NUMT of *16S*), also published by Takahashi et al. (Additional file 2: Selected scaffold sequences). Further, we identified a fragment (around 350 bp) from the Eu17 (scaffold-250,790) and Eu36 (scaffold-122,170) Ray genome assembly data for *E. uchidai* (Additional file 2: Selected scaffold sequences) that was identical to the nucleotide sequence of LC314404.1 (NUMT of *COX1*). We were not able to observe the 3′ end of the nucleotide sequence, which contains the reverse primer khCOI-R [1] region. We were therefore unable to amplify the NUMT of *COX1* using the primer set of Takahashi et al. [1]. It is likely that the NUMT of *COX1* occurred in the common ancestor of *E. asanumai* and *E. uchidai*, and that the 3′ part of the sequence was lost in the *E. uchidai* genome. We were also not able to observe the NUMT of *16S* in the Ray genome assembly data for *E. uchidai* (Eu17 and Eu36), or that of *COX1* or *16S* in the data for *E. hatanakai* (Eh1 and Eh6).

Although Takahashi et al. [1] checked that there were no gaps in the *COX1* or *16S* rRNA compared with other species and that all nucleotide changes, which are subject to functional constraints, in *COX1* were synonymous, this was not sufficient, since further analysis (such as RT-PCR) would have been needed to avoid NUMTs. The entries in the DDBJ/EMBL/GenBank International Nucleotide Sequence Database (LC314404.2 for *COX1* and LC314405.2 for *16S*) have been corrected accordingly.

Although both of the corrected nucleotide sequences for *E. asanumai* were more similar to those of *E. uchidai* than the previous sequences, they nevertheless formed distinct clusters in the phylogenetic trees (Figs. 2 and 3). Nucleotide differences between *E. asanumai* and *E. uchidai* ranged from 5.5 to 6.0% for *COX1* (Additional file 10: Table S5) and from 1.8 to 2.0% for *16S* (Additional file 11: Table S6).

### The high GC content of the mitochondrial genomes of *Eubranchipus* species

Luchetti et al. [9] showed that the mitochondrial genomes of the Anostraca and Onychocaudata (*L. lenticularis* + Cladocera) have a significantly higher GC content than those of Notostraca species. According to these authors, this can be explained by a preferential AT to GC substitution bias during the evolution of the Anostraca and Onychocaudata lineages. In this study, we observed differences in substitution bias among Anostraca species. The Artemiidae and Chirocephalidae tend to have a higher GC content than other Anostraca species. Although *Eubranchipus* species have a similar or higher GC content than *Artemia* species, we did not find any significant differences between the Chirocephalidae and Thamnocephalidae + Streptocephalidae (Table 2). For this reason, *E. grubii*, which has a relatively low GC content, is included in the Chirocephalidae. If *E. grubii* were eliminated from comparisons, the number of species of Chirocephalidae would decrease, and statistical tests could not be performed. Thus, we suggest that Far Eastern *Eubranchipus* species also have a relatively high GC content, equivalent to that of *Artemia* species. We infer that the higher GC contents of *Eubranchipus* and *Artemia* species than that of the Thamnocephalidae and Streptocephalidae are also caused by a preferential AT to GC substitution bias. In this study, however, the number of *Eubranchipus* species analyzed is limited. Further investigation, including other *Eubranchipus* species, is thus needed to clarify the differences in substitution bias among Anostraca species.

### Phylogenetic tree of the mitochondrial genome data for Branchiopoda

The current knowledge on the phylogenetic relationships of Branchiopoda is that Anostraca diverged first, followed by Notostraca and Diplostraca (Cladocera and Spinicaudata) [9, 40–44]. The topologies we obtained (Fig. 4, Additional file 7: Fig. S2, and Additional file 8: Fig. S3) support these relationships.

Luchetti et al. [9] indicated that the Anostraca and Onychocaudata (Diplostraca) have a significantly higher substitution rate than the Notostraca, similar to what we found (Table 4). Further, we observed differences in substitution rates among the Anostraca: accelerated substitution rates within the Artemiidae and Chirocephalidae lineages. In this study, we assumed an acceleration of the Chirocephalidae lineage, including *E. grubii* (Additional file 9: Fig. S4); however, there is also the possibility that the acceleration occurred in the common ancestor of the Far Eastern *Eubranchipus* species. Because we used only one species, *E. grubii*, as out of Far Eastern *Eubranchipus* species in the study, further investigation including other *Eubranchipus* species is needed to clarify whether the acceleration occurred in the common ancestor of the Chirocephalidae or that of the Far Eastern *Eubranchipus* lineage. It is likely that substitution rate accelerations in the Artemiidae and Chirocephalidae lineages occurred independently, because the Artemiidae and Chirocephalidae do not form a monophyletic cluster (Fig. 4). This phylogenetic relationship is similar to that reported in previous studies [7, 45–47].

Accelerations of substitution rates in the Artemiidae, Chirocephalidae, and Diplostraca lineages are not associated with increases in nonsynonymous substitutions, because the overall ω values of the Artemiidae (0.109), Chirocephalidae (0.106), and Diplostraca (0.166) do not differ from those of the Thamnocephalidae and Streptocephalidae (0.121) or the Notostraca (0.138). Lower ω values (< 1) were commonly reported in previous studies [9, 18, 21, 22, 28]. This is due to selective constraints acting on the genes of mitochondrial genomes.

For the Artemiidae, it has been suggested that halophilic habits may be correlated with accelerated substitution rates [48]. This is not the case for the Chirocephalidae because members of this family inhabit freshwater bodies. Other potential reasons for accelerated substitution rates such as body size, temperature, generation time, and population size have been considered [49–52]. The typical body length of Anostraca is 1–5 cm [53], and the three *Eubranchipus* species are within this range [1, 2, 4]. Generation time is difficult to estimate for Branchiopoda. Most branchiopod eggs are drought-resistant and can remain dormant for decades under anoxic conditions, and during pool inundation, only a fraction of each egg clutch hatches [53]. *Eubranchipus* species also have this “bet-hedging” strategy. It is thus not straightforward to estimate or compare generation times among Anostraca species.

We infer that the effect of population size is the most plausible reason for the accelerated substitution rate of *Eubranchipus* species. It has been suggested that evolution occurs rapidly in small populations [54]. Habitat for *Eubranchipus* species is rather limited in Japan. Presumably, a few eggs originally came from another location to the current habitats a long time ago, and acceleration of the substitution rate occurred in these small populations, after which the population sizes increased rapidly over several generations. The genetic diversity of these species would therefore also be expected to be small. In this study, we used mitochondrial genome data for our phylogenetic analyses, but it is also necessary to conduct investigations using nuclear genome data, to better understand the substitution rates and genetic diversity of *Eubranchipus* species.

## Conclusions

In the current study, we present six new mitochondrial genome sequences from three *Eubranchipus* species. These are the first reports of the entire mitochondrial genome sequences of these *Eubranchipus* species. We show that these species shared the anostracan pattern of gene arrangements in their mitochondrial genomes. We observed a higher GC substitution bias in *Eubranchipus* than in other Anostraca species. We noted that the *COX1* and *16S* sequences presented in Takahashi et al. [1] were NUMTs, and we have corrected these in the present study. We also conducted a phylogenetic analysis of Branchiopoda species using mitochondrial genome data. We observed accelerations of substitution rates within the lineages of *Eubranchipus* species. Higher GC content and accelerated substitution rates are the specific characteristics of the mitochondrial genome of *Eubranchipus*.

## Methods

### Samples

*Eubranchipus uchidai* specimens were collected from a temporary snowmelt pool in Ishikari, Hokkaido, on 29 April 2017. *Eubranchipus asanumai* specimens were collected from a temporary snowmelt pool in Shiretoko, Hokkaido, on 18 May 2018. *Eubranchipus hatanakai* specimens were collected from a temporary snowmelt pool in Yuza, Yamagata, on 10 April 2018.

### Genomic DNA extraction

Two *E. hatanakai* individuals (sample IDs: Eh1 [male], Eh6 [male]), two *E. uchidai* individuals (sample IDs: Eu17 [female], Eu36 [female]), and two *E. asanumai* individuals (sample IDs: Ea3 [male], Ea4 [male]) were used for genomic DNA extraction and sequencing. Each individual was homogenized using a disposable BioMasher II homogenizer (Nippi, Tokyo, Japan). The genomic DNA was extracted using the conventional sodium dodecyl sulfate lysis and phenol–chloroform method and RNase A (Sigma, St. Louis, MO, USA) was used to digest any contaminated RNA. Then, to remove RNase A proteins, the genomic DNA was purified using a NucleoSpin gDNA Clean-up Kit (TaKaRa Bio, Kusatsu, Japan). The quality of the extracted genomic DNA was checked using an Agilent 2200 TapeStation (Agilent Technologies, Santa Clara, CA, USA).

### DNA sequencing

A sequencing library was constructed using the Ion Xpress Plus Fragment Library Kit (Thermo Fisher Scientific, Waltham, MA, USA), and sequencing was performed using the Ion Proton System (Thermo Fisher Scientific). Approximately 6 Gb were sequenced per sample. To correct homopolymer errors in the fastq data, we used Pollux 1.0.2 [10] with option “-k 31”. The optimal k-mer length for the corrected fastq data was then estimated using KmerGenie 1.7016 [55].

### Sequence assembly

The de novo genome sequence assembly was performed using Ray 2.1.0 [11] with option “-k”. The k-mer values used are shown in Additional file 1: Table S1. Next, standalone BLAST (tBLASTn) searches [12] were performed using 13 amino acid sequences from the mitochondrial genome sequence data of *E. grubii* (MT410793.1) as queries against the Ray assembly data. The longest mitochondrial DNA sequence obtained for each dataset was used as the seed for the mitochondrial genome sequence assembly in NOVOPlasty 3.2 [13]. To check the sequence read depth, read remapping to the assembled mitochondrial genome sequence was performed using BWA 0.7.12 [56] with the “aln” option and SAMtools 0.1.19 [57]. The remapping was performed using the pipeline of the Management and Analysis System for Enormous Reads (Maser) of the Platform Project for Supporting Drug Discovery and Life Science Research (Platform for Drug Discovery, Informatics, and Structural Life Science) [58]. The Integrative Genomics Viewer 2.8.13 [59] was used to visualize mapped data. Mapped sites were counted using igvtools [59].

Gene annotations were performed using the MITOS WebServer [60] and manually corrected by following the annotation of the complete mitochondrial genome of *E. grubii* (MT410793.1).

Average genetic distances in the *Eubranchipus* species for each locus were calculated using the K2P nucleotide model [61] in MEGA7 [62].

### Reanalysis of mitochondrial DNA sequences of *E. asanumai*

We used one *E. asanumai* individual (ST-M2) from the same locality as the paratypes. It was collected on 28 May 2016 and had been stored in ethanol. Its whole body was cut in half along the median plane; one half was used for genomic DNA extraction, and the other was used for total RNA extraction. Each half was homogenized independently using a disposable BioMasher II homogenizer (Nippi, Tokyo, Japan). Genomic DNA was extracted using the conventional sodium dodecyl sulfate lysis and phenol–chloroform method. Total RNA was extracted using TRIzol Reagent (Thermo Fisher Scientific) and treated with DNase I (Nippon Gene, Tokyo, Japan) to digest any contaminated genomic DNA. Next, to remove DNase I proteins, the RNA sample was extracted using TRIzol LS Reagent (Thermo Fisher Scientific). Extracted DNA and RNA were confirmed using 1% agarose gel electrophoresis.

The PCRs for *COX1* and *16S* sequences using the genomic DNA were performed using the same primers and PCR conditions as used by Takahashi et al. [1]. The RT-PCR using total RNA was performed in one tube in a 5.0 μL mixture containing approximately 0.5 μg total RNA, 0.2 μM of each primer, 1× KAPA 2G Fast Multiplex PCR Kits HS (KAPA Biosystems, Wilmington, MA, USA), and 10 U of SuperScriptIII Reverse Transcriptase (Thermo Fisher Scientific). The reaction conditions were 50 °C for 30 min and 94 °C for 2 min, followed by 35 cycles of denaturation at 94 °C for 15 s, annealing at 50 °C for 30 s, and extension at 72 °C for 30 s. The same primers for the *COX1* and *16S* sequences were used for RT-PCR. The PCR products were confirmed via 1% agarose gel electrophoresis and purified using a High Pure PCR Product Purification Kit (Roche Diagnostics, Mannheim, Germany). DNA sequencing was performed on the PCR products using a BigDye Terminator v1.1 Cycle Sequencing Kit and the ABI PRISM 310 Genetic Analyzer (Applied Biosystems, Waltham, MA, USA). To confirm the sequence, both strands of DNA were sequenced.

Phylogenetic analysis of *COX1* and *16S* was carried out following Takahashi et al. [1]. Multiple alignments were performed using MUSCLE [63], implemented in MEGA7. The ML method [31] was used to construct phylogenetic trees from the *COX1* and *16S* data using RAxML [64] with the general time reversible (GTR) [65] + γ model. Bootstrap probabilities [66] were computed from 500 replicates. Pair-wise p-distances as percentages were also calculated using MEGA7.

### Phylogenetic inference among Branchiopoda species

The nucleotide sequences of 13 protein-coding genes were retrieved, concatenated, and used for the analysis. Multiple alignments for the translated amino acid sequences of each gene were performed using MUSCLE [63], implemented using MEGA7. Sites containing gaps were removed. The translated amino acid sequences were then returned to the nucleotide sequencing data. The GC content was calculated using MEGA7.

ModelTest-NG 0.1.6 [67] was used to select the best-fitting nucleotide substitution models for the genes. The “-T mrbayes” option was used to select models for the Bayesian approach [34].

The ML method was used to construct a phylogenetic tree using RAxML-NG 1.0.1 [68] with 1000 bootstrap replicates. The BS approach was used to construct a phylogenetic tree using MrBayes version 3.2 [69] with 10,000,000 generations. Nucleotide substitution models selected by ModelTest-NG 0.1.6 were used for both the ML method and the BS approach. The NJ method [36] was used to construct a phylogenetic tree with 1000 bootstrap replicates in MEGA7. The “Find Best DNA/Protein Models (ML)” option of MEGA7 was used to select the best-fitting nucleotide substitution models for the NJ tree. To compare the topologies obtained from these three methods, we conducted the likelihood analysis using BASEML with GTR (also known as REV) + γ in PAML version 4.4 [70]. Differences in substitution rates among lineages were tested using BASEML with GTR + γ. We used CODEML in PAML version 4.4 to estimate the ω values, and performed the Kishino and Hasegawa test [38] and the Shimodaira and Hasegawa test [39].

## Supplementary Information

**Additional file 1 **: **Table S1**. Summary of sequencing statistics.

**Additional file 2.**

**Additional file 3.**

**Additional file 4 **: **Table S2**. Annotation of mitochondrial genes in three Eubranchipus species.

**Additional file 5 **: **Table S3**. Genetic distance among Eubranchipus species.

**Additional file 6 **: **Table S4**. Selected nucleoitde substitution models.

**Additional file 7.**

**Additional file 8.**

**Additional file 9.**

**Additional file 10 **: **Table S5.** Pair-wise nucleotide difference per site in % for COX1 data. Compared sites were 487 bp.

**Additional file 11 **: **Table S6**. Pair-wise nucleotide difference per site in % for 16S rRNA data. Compared sites were 399 bp.

## Data Availability

DNA sequences determined in this study were deposited in the DDBJ/EMBL/GenBank International Nucleotide Sequence Database (accession numbers: LC633437–LC633442).

## Abbreviations

12S: 12S ribosomal RNA
16S: 16S ribosomal RNA
AIC: Akaike information criterion
ATP8: ATP synthase F0 subunit 8
ATP6: ATP synthase F0 subunit 6
BIC: Bayesian information criterion
BS: Bayesian
COX1: Cytochrome c oxidase subunit I
COX2: Cytochrome c oxidase subunit II
COX3: Cytochrome c oxidase subunit III
CYTB: Cytochrome b
DDBJ: DNA Data Bank of Japan
DIN: DNA integrity number
EMBL: European Molecular Biology Laboratory
GTR: General time reversible
K2P: Kimura two-parameter
ML: Maximum likelihood
ND1: NADH dehydrogenase subunit 1
ND2: NADH dehydrogenase subunit 2
ND3: NADH dehydrogenase subunit 3
ND4: NADH dehydrogenase subunit 4
ND4L: NADH dehydrogenase subunit 4 L
ND5: NADH dehydrogenase subunit 5
ND6: NADH dehydrogenase subunit 6
NJ: Neighbor-joining
NUMT: Nuclear mitochondrial DNA segments
PCR: Polymerase chain reaction
rRNA: Ribosomal RNA
RT-PCR: Reverse transcription polymerase chain reaction
