# Supplementary material for: Complete mitochondrial genomes of three fairy shrimps from snowmelt pools in Japan
Source: BMC Zool. 2022 Feb 9;7:11. doi: 10.1186/s40850-022-00111-2 (PMC10127424; doi:10.1186/s40850-022-00111-2)
Supplement: Supplementary file 2 — Additional file 2. [file 40850_2022_111_MOESM2_ESM.pdf]

>Eh1_scaffold-325202
CCCCATACCTCCCCTCTCTACAGATAGCCTATATGTCCTATCTACAGTAAGATGCTATAGAGT
ATGGGACTATTTACTCACGACCCAGGATATGATCTATGCAGGTGACCATCCCAGCAAGGCTA
AGACGGTCTATTAGAATAAGAGATTTAGATAAAATATCGTAGGCTGTCTGGTACTCTACTATA
GGGGCATAAGAGAGTTGTTTTCCTATGAGAGGGCCAATTACAGAACAGCAATAATCAAATTC
ATGATTTCTATACGAGGATCCCGGTATCCTTCTAATCACCCAAATGCACGAATTCAATACCCC
GAATATCTCCTCCTTTCTTAGATCCTCTACTCCAGTACCCGAGGGTACTATAGACATAAACTC
TTGACGAAGAATAGATACAGCGATACCCCGATTTTCTAGAACCAGATGTTCTGAGTTATCTCT
GAACCTTAGGATTAGTTGAGCGTGGAGATTGTCCCATTCATCACACATAAGAGCTCCTACGC
ACACGTCATTAACAGAAATTGCCTCTAAGTGAGCAGAGTGAAGAATTGCATGAAAACCTAGT
ATATCACCTTGAGTTTGGTACCTATCTAACAAAGTCTTTGCTTCTTCTATAGGCATAGGAGGA
GTAAATGTGGAGACATTAGTCCCAAAAGCAACATGAAAAGCTCCTGCGATCTCTTCAAAGAC
TAAACGTGGGACACAAGAGTCATCCCCGTATTCTACCATTGCGGGGTGTGACCTTATACCCA
CTACAGTGATAAGGTGTCTTTCCCCAAGACGTCATATCGAAAGGGGGAAATCGTCTATCTTT
GCGTCTACAAAATCCCGTGTAATAGATGAAGTGTTTTTATTAATATTAATTCTTAGGATAGAC
TGATTTTGGTGATATTGAGTGAGCAACCTTTCTTTGGCCATGAGGAAATTATTAAAAATAAAT
CTATGGGTTGTCCCAGATCAATCTTCTCCCACTAAAGCGGGGGAAGGGGTACGACTTTCTCC
CGAGGAGAGATTCCCGTTATGACTAGTATTATTAAAAAGACAGGGAGGAGAATCTGGCAGGC
CTATACCTTCAAAAGTAGGATCTAAACACTGAGGTCTCAGAGATTGGGCAAAAAAAGGAAAG
GAAAGTTCAGTAGTAATAAGAGAAGAAAGTACTCTATCAGTCCAAGAAATAAAAACAAGCCC
AGTAACAACCAATACTCAAAGCAGAAACAAAAAGACTGGGTCATAGGAATGTGTCCTCTTATT
TGAGGACAAAAATAAGATTAGGGATAAAAATATGAAGATAAATAGGAATTCAATTAGATTTAC
TACGAGGCTATCGCTCCAGTCTTAGACCAAGATAGGCTTAACAATCTTTTAAAGGATGGGGC
TTCATTCCTCTGCCTTCCAACAGCAGCATCTTCGATGACCTTTACCTTAATTGAAGATCAATT
TTATTGTTCTTGATGAAATTTAGTATTTTATTTTTCATGGAGAAATTAATTACTGAAGACTTTA
CTCTGTCTAACTAGTATAAAACATCAAATTAGTTTATACCCGTAGTTTACTATTCCAAAGAGG
TAAGCTAAACAATAAGCTATCAGGTTCATACCCTGCCGATAGAGTCATCTCCTCTTTAAAGAA
ACACCAAATTCTTGATAATAAAATGACTAATTTTAGCTCTATCCTATATTTTGATATTCTCAAC
TGAATCTTGACTAGGGCTATGATTGGCCCTAGAATTAAACGCCCTTAGATTTATTCCTATTTT
GGTATGGAGGGACAAGGAAATAAGATTAAAATATTTTTTAGTACAATCTCTAGGTTCCCTTCT
GTTTTTATCGGGGGTCATCAACCCATGACTCCATGTTGGTCTGATCTGTGGGCTTCTTTTAAA
AATCGGAGCAGCCCCTCTTCACCTATGAGTCCCAGCAGTAACACCAAATATATCATGAGTCT
CTCTATTTTTTTTATTAACCTTTCAAAAACTAGGGCCTTTACTTGGACTTCTAATGCTTACATT
GGATAGGGGGGCTATCGTGATACTCTCAGCACTCATTGGGGCTTTAGGAGGTATCCTGCAAT
CCAATTTGCGCTTACTTATTACCTACTCTTCTATCACACACTTGGCGTGAGTGTTTATAAATA
TAAAGAGTCTACTACTACTAATGTCTTATTTTTGGGTATATACACTGATTACCGCAACTCTAG
TTCTGATCTTTCAGAGATTGGGTGCGTATTTTGTCAGGCAGATAAATAGCCCCATGACGGCA
GGGATAAAGAGAGCTCTTGGCTCTTGTCTTCTTTCTTTGGGAGGACTTCCACCTCTTCTGGG
GTTTATAATTAAGTGAATAACTTTGGAAAACTCAGTGCCTTCTCTGGTGATCCTGACCAGACT
TGTTTCATCGACATGCATGTCTCTATTCTTTTATTTTAAAATTATAATAATTCCTATTTTATCA
CCTTTGTCTGTTTCTCGGGGTGATTTCTGGACGTGATTAGTAATTGGATTTCACTGAATAGTA
CCTGTGGCTATGCTTTAGAACCTTAAGTTAATATAAACTAGTAGCCTTCAAAGCTGACCATAA

GAAAATCTTAGGTTCTAGACAGGGGCATTTATGCATATTTACAGTATCAACGTAACCCTTTAA
TCAGGCACCCTGTCTTTATACCTTTATACCTAGAGATTAGAACTCTACTCTAAGGATCAAAAC
CTTAAGTGCACGATACACCAAGATATAAAATCTCAGCCAAAGGCACCTAAAGATTTGCAATCT
TCTATTCTTTTAAACTATAAGACTTAGTAGAGGGAGGACGACTCCTTGTCAAGGTTTACAGCC
TTACACCTAAATCAGCCACTCTACTTTTGCAACGATGACTCTATTCCACTAATCACAAGGATA
TCGGCACGCTTTATTTTTTATTCGGAGCTTGATCAGGAATGGTAGGCACTTCTCTTAGAATAC
TAATTCGGGCCGAACTAGGTCAGCCAGGATCCCTGTTAGGAGATGAGCAACTTTACAATGTT
ATTGTAACCACCCATGCTTTTGTCATAATTTTCTTTATAGTGATACCTATTCTCATTGGGGGG
TTTGGTAACTGACTAGTCCCAATCATATTAGGAGCACCAGACATAGCTTTTCCGCGTCTTAAC
AACTTAAGATTTTGAATACTACCACCATCACTAACTCTCCTGTTAGCGGGGTCTTTAGTAGAA
AGGGGAGCAGGAACCGGGTGGACAGTATATCCTCCTCTCTCGTCAGGAATCGCTCATGCAGG
ACCGTCTGTAGACCTTTCTATCTTTTCTCTCCATCTCGCAGGGATCTCTTCGATTCTGGGGGC
AGTTAATTTCATTACAACTATCTTGAACATGCGTCCCCAATCAATGTCATTAGACCGAATACC
ACTTTTTGTGTGGTCAGTTGGAATCACAGCAGTTCTTCTTCTTTTATCTTTACCCGTCTTGGC
AGGAGCTATTACAATGCTACTTACGGATCGAAATTTGAACACATCGTTCTTCGACCCTGCAG
GAGGGGGGGATCCTATCCTTTATCAACATCTCTTTTGATTTTTTGGACACCCTGAGGTGTATA
TTTTAATTCTGCCAGGGTTTGGTATAGTGTCCCATATTATCAGGCAGGAAAGAGGGAAAAAAG
AAGCATTCGGCACTTTAGGTATAATCTATGCTATACTGGCAATCGGAGTTCTGGGATTCGTGG
TCTGGGCGCATCACATATTCACAGTAGGTATAGATGTTGATACACGGGCTTATTTCACAGCTG
CCACTATAATTATTGCGATCCCTACAGGAATTAAAATCTTTAGATGAATTGGAACGTTGCACG
GTACTCGGTTTACTATAAGACCTTCACTTATGTGAGCCCTAGGATTCGTATTCTTATTCACTG
TGGGTGGACTAACAGGGGTGGTATTGGCTAACTCTAGGGTCGATATTATTTTACACGACACC
TATTACGTTGTAGCACATTTTCACTATGTTCTTTCAATGGGAGCTGTATTTGCCATTTTAGGG
GGATTCACACACTGGTTCCCACTTCTAACAGGAGTATCCCTTAATACTACTTACTTAAAAGTT
CATTTCATAATTATATTCTTGGGAGTAAATGTTACGTTCTTTCCTCAACACTTTCTTGGACTAG
CAGGGATACCACGGCGATACGCCGATTACCCAGACAGCTATGCTTCTTGGAATATGGTCTCT
TCAGTCGGAAGAATTATCTCTTTTATAGGGACCCTGGGGTTTATCTTTTGCTTATGAGAGGCC
TTTACCTTAAAGCGGGCAAATGTGTTCTCCCTTAACCTAAGATCTAACATTGAGTGACATCAC
CCCCATCCTCCTGCTGACCATAGCTACGAAGAACTCACTCTCGTCTCTTCTTTCTAATGTGG
CAGAAATTATATGCAGTGGACTTAAGCTCCATCCAAGGGAGTTTCCCCTTTAGAAAATGTCTC
AGTGATTTCAGTTAGGACTACAAAATGGGGTGTCTCCTTTGATAGAACAGTTAGTATTTTTTC
ATGATCACGCTCTACTAATTGTAGTATTGATCACGTCCTTGGTAGGATTTTTTATAGTGTCTTT
ATTTTTTAATAATTACGTACACCGCTATTTGCTAGACGGACAGATAATTGAGACCATTTGAAC
TGTTCTCCCTGCAGTAATCCTGATTTTTATCGCTTTGCCCTCTATTCGGCTTCTTTACTTAATT
GATGAAGTACAAAACCCAGCCCTAACTATTAAAGTAACAGGGCACCAATGATACTGGGGGTA
TGAATACTCAGATTTTAATGATCTCCAGTTTGACTCTTATATAGTACCTACTCAAGACCTAGA
ACAAGGACTATTCCGCCTTCTGGATGTAGACAACCGAGTACCTCTCCCTTTTAATCAGCCAA
TTCGGCTTATAATTACATCCGATGATGTTCTTCATTCATGAGCTCTTCCTTCTATTGGGATCA
AGATAGATGCTATCCCAGGGCGCTTAAACCAATCGGGAATACTTATTAACATACCAGGCATT
TTTTATGGGCAATGCTCGGAAATCTGTGGAGCAGGCCACAGGTTTATACCAATTGTTCTTGAA
TCGGTAGACACTAAAAGATTCCTCAATTGATTACAGAGACAAATTTCATAAGATGGCTGACAG
AGAGCAAAGGCTTTTTAAGCCTTAGTAGGGTGTTACTACCTCTTATGGCAGAAATTGAGTTTT

ACAAGAATAGGAGAGTGTCATTCTCCAGGTACTCAAAGAGTAATTTCTTATACCTCAAATAGC
TCCTCTCCCGTGAACAACCCTATTAGTCACTTCAGTTCTAATCATTATTTTAGTAATGACTAT
GACTTATTTCATCGACCAACCCATCGCCCTTAAAAGGAATATCAATACTCCCCGAGTAAGAG
TAAATAGTTGAGTATGATAACTAACCTATTTTCAGTGTTTGATCCTACATCCTCTCTTTTAACC
AACTGGCTGTCCATGACATTAACGTTTATTATACTACCATGAGCATTCTGGGCTATACCTAGA
CGAGTTCAGGTGATATGGGATACCGTCATGATAAAATTAGACCAGGAATTTTCTCTTTTGATA
GGACCCAATAAAAGGGGGTCACCACTTTTACTGGTGAGAGTTTTTTCATTCATTCTATTCAAT
AACTTGATGGGGATATTCCCGTACCTATTTACTGCAACTAGTCATCTAGCCATTACTCTAGCT
CTAGCACTTCCTCTCTGGCTATCTTTCCTGCTGCATGGATGAATTAAAGAGAGTAAACACGCA
CTAGCACATTTAGTACCTCTAGGTACTCCTCCTGTTTTAATACCTTTTATGGTAGTAATTGAG
TTGGTGAGTAATTTTATCCGGCCTATCACCCTTTCAGTACGACTCGCTGCCAATATAATTGCT
GGACATTTACTTCTCACCCTTCTAGGCAACCAAGCAATTGGGGCAGGAGTGTTGCCGGTACT
GGTTGTGTTAATAAGACAAGTAATGCTTCTAGTACTTGAGTTTTCTGTAGCAATTATTCAAGC
ATACGTGTTTGCTACCTTATCTACTCTCTACGCCAGGGAATAATGACTCAACTGAACCATCCA
TACCATATTGTGAACGTAAGACCGTGACCTATTGTTAGAGCCTTAGCAGCTTTTAGGCTGACA
ACAGGGCTTGTCCAATGATTTCACCAGTTTAACTCGTCTCTTTTCATTCTAGGAGTTGTGACA
GCATGCATCGTATCAATGCAGTGATGACGTGATATTAGACGTGAGAGAACACTCCAAGGACA
TCATTCTTCCAAAGTAGGAAAAGGACTGCGATGAGGCATAATTCTGTTTATCGCGTCTGAAAT
TTTTTTCTTTGTATCATTTTTTTGAGCTTTCTTTCACAGAAGACTCTCGCCCAATGTAGAAGTA
GGAGCAGTATGACCACCTGTGGGCATTGAAGGGTTTAATCCCTTCCAGATTCCCTTATTAAA
CACAAGAATTCTTTTGGCGAGAGGAGTAACTATTACGTGAGCTCACCATGGATTGATAGAAA
ACAACTTTGACCAGTGTAAACAGGGACTGCTCCTTACTGTTATTCTAGGGGTATACTTCTCGT
TTCTTCAAGGAGTAGAATATATTGAGGCATCATTCTCTATCGCTGATAGAGTTTACGGGTCCA
CCTTTTTTGTAGCAACGGGATTCCATGGACTCCATGTCATTATTGGCTCATCATTTTTAATAG
TATGTCTCGCACGTCATTTACGATGTCATTTCTCCCAAGAGCACCACTTTGGATTTGAAGCAG
CTGCTTGGTACTGGCATTTTGTAGATGTAGTGTGACTCTTCCTCTATGTTTCTATCTACTGAT
GAGGTGAATAACTTACTTAGTATATAAGTATATCTGACTTCCAATCAGAAGGCCCACATGTGG
AGTAAGTAATTGTTTGGATTGCCCTACTGTGAATTTCTCTAATCATGCTCTCTACTATTATGTT
GTTAGCCAGCAGAGTGATTAACAAAAAAGCAATTATGGAACGAGAAAAAAACTCACCTTTTG
AATGTGGGTTTGACCCCCAAAATTCAACACGAATGCCATTTTCACTGCGATTTTTTGTCATCA
CACTTATTTTCCTTATCTTTGATGTCGAAATCACACTTTTGCTGCCAATTAATTCTTTGATAGG
GTTAGAATTAACAAGAGTCACAGTCTTTGTTTTCTTTATTGTCATTTTAGTGACAGGAGTTTTA
TACGAATGAGAAGAAGGGGCACTTAGGTGAATCAAATAGGAGCGTAGTTTAATTAAAATACTT
GATTTGCAATCAAGAGAAGACAAGCGTCTGCTCTCGGGAAAGGAATTGATGTATCATCCTTA
GTTTCGACCTAAAATTAGGCCTTGAGCCCCTTTCTTTAGTAGAAGCTAATAAGCAATCCACTG
TTACTGGAGAGAGAGGATTTGTTTATCCCTATTAAAAAATGAAGAGGAACCAAGAAAAGCTTC
TAACTTTGCCTTGAGATAGTTTAACTCTATCTTTTGTTTGCTTTGATAGTATAATAAATACATC
ACATTTTCAATGTGAAGATAGGGTCACTCTTTAAAGCTATCCCATGAAATAGCTTATTAGTCA
AGATGATAGAGGAGATAGGGGGCCTCTCCTTCTCCTGTATACT
>Eh1_scaffold-21
TATGTCCCTAGAAAAGAATTCACCCTAACATCTTCAGTGTCATGCTCTTATTAAGCTATAGGA
ACTAAACAAATCAAATCCCAGTAAAAGACACTAAAAGGAGAAGTTTAAAACCTGTTGACATAA

AGATTTCTCTCAAAGAACTCAAAAAAAGAATCCCTGTCTTCTGAGCACCGGGGCCCAAATAT
TCTACTCAACCGTGATCTCCCTGGCTGTAAATGATGTCTCCTCTTTTAAGCAATGGCACCCTA
AGAATAGATGTTAAATAAGGAAGGAAAGTTATAGTGGTTAAGAAATCAAACACACGCCCCTTA
CTCGCTGTTATCCTAAAACCTGCTAAAACCCCCACCATAATACACAAAAGCAAAGTTAATTTT
TCTATAGAAGAAACTATAAGACTTTCTGTACCTAAAAAAAATCAATAAAACAAGGATCCCCCG
AATAAGGCCCCTCTAAACAAAACCTTAAGAGGAGTAGAGTAGTTCCCTTCATCCCCTCTAAT
AAAAGAAACTCAGTTAAAGACTGAAGAAGTAAGTGCCACTGAAATAAGACGAAAGGAATAGC
CGCAGGTTAAGAGAGCAGCAGCAAGGATAGCGAGGGAAGGGAATATTAGTAATTCTCTAGTA
GAACTCTCAATAATTGTGTCTTTAGAATAAAACCCAGCTAAAAAAGGAAACCCCATTAAGCTA
AAGGAAGCAGCCCTAAGAGCTAAGCAAGAAAACGGAAGAAAGAGGGAAACTCCCCCCAAAC
GACGAATATCCTGAGTACCCCCAGAGGAATGGATAATAACCCCAGAACACATGAACAAGAGA
GCCTTAAACAAGGCATGAGTAAATAAATGAAAGTAGCATAGAAGAGGGCAGCCTACCCCCAA
AGAGAACATCATCACACCTAGTTGTCTTAGAGTAGAGAGGGCAATAACTCGTTTGAGGTCGT
ACTCCGTTATAGCCACTAGACCGGAGAGAAGTGCAGTAATAGAACCGAGTAAAATTAAAAGG
AGATTGCCTGAATGGGAAAAGCACGGAGAAAGACGAACTATCAAAAAGATGCCTGCTGTTAC
AAGAGTAGACGAGTGGACAAGTGAAGAGACAGGGGTAGGAGCTGCTATAGCCGCGGGTAAT
CAAGCTGAAAAAGGAATTTGAGCCCTTTTAGTTAAAGACGCCAATAAAAGAAGAATTAAAACT
GTATAAGAGGACTCGGCAGAAATAAAACTGTAATCCCAAGAACCAAGAGAGTAAGTAAGACC
TAGAGATCAGAGAATAAGAATATCTCCTACTCGGTTACTTAGAGCAGTAATTATTCCTCTTGA
AGAAGATTTAGGATTGGAGTAAAAAAGAATCAAAGCATAAGAAGTAACTCCTAGCCCATCTC
ACCCTAATAAAAGACTCACACCGTCTGAAGAAATGATAAGAAAAGCTATGGAGAGTACAAAA
AACAACAATAGGTACTTAAACCGGTCATAAAATACCTCCCCTCCCATGTAATAAGAAGAATAC
ATGAGAACTTGAGAGGAGATTAACCTTACAAGGAAAAGAAAAGAAAGAGAAATTCAATCAAA
GTGTACTTGATAAATAAAGGAGAAGAAAGAAACTGACACGAGATAAATAGAATCACTCAGAT
GGGATAAGAAGGATAACCCTAAAAAGGTACATCCCAAAACAGAGAGAAAAAATCTATAAATG
TAGTAAGTCAAAGTTCTTGGAGATATTCACCGCTAGAATCACAATCTAGAATCCTTCTTTAGA
CTACAAGAACATAAAGTGACTAACAAAAGGAGGGGGATGAAATGGAGAGCTCTTACTAGAAG
TTCATTAATGTCTGAATGTTGAAGACCAGTCTTTATTAGAGTCTCTCCGTGAATAGTTCAAGA
GTATAGGAATAAGCAGTAGCAAGCTGACAAGAATGAAATTAAGCCTGCTAAAACAAGGAATG
ATAAGTGAAGACCGCCGGAAGCGATAAACAAGTAAAGTTCCGAAAACAAGTTTAGAGAAGGA
GGAACCCCCATATTAAAGATGATAAGGAGAAATCATCATAAAGCAATAAAGGGAGAAAAAAC
AATAATACCACGAGTTATTAAGAAAGAACGAGAGCCAAAATAAGTGTAAATTAGATAACTTAG
GTAAAAAAGACCTGAGGAACAAAGACCATGGGCGACTATCAACAGGAGGGAAGCTCTATTCC
CATAACTTCCCATAAATAGCATCCCTAAGATAATAAATGCTATATGAGCAACCCTAGAATAAG
CGATTAAGGACTTCACATCAGTTTGACGGAGTGAGAGCAGACATCTTAAAACGCCTCCTCAT
CCGGCTAAGAGACAGATATAGCCGAGTAGACTTACCCCTATTAAAGGTTGCACTAAGAATAG
CCCATACCCGCCTAGCTTTAATAAGATAGCAGCTAAAACCATTGAGCCAGTGACAGGAGCTT
CTACATGAGCTTTAGGCAATCAGACATGAGTAAAGTATATGGGCAACTTAACTAAAAAGGCG
AAGACTATAAAGAACACAAAGAGTTTGTCTGCACTGGAGGGAAGAAAAAAAGAGGTTGAAGT
CTGGTACTCTATATCTAGGCCCAAGAGAATAAAGAGGAGAGGCAGTGAAGCTAAGAGGGTAT
AGAATAAAAAATAAAGGGATGCGGGGAGACGCTCGGGCTGGTAACCTCAGCCCATAATTAAG
AGAAGGGTGGGGATTAGAGAGGACTCAAAAAAAATATAAAAGGAGATAAGAGAATTGGAAGT

GAATGTGAGGACTAACAGCAAAAAGAGAAGAATAATTAAAATGCTGTAGGTACGCTTATAGG
AAGGGGATCAGCATATAATCATTAAGAAGAAAATTCACCCCCTAAGGATAAGAAGATAGTGA
GATCAACTGAATTTGAAAGAAGAACACAAAATAAGAAATGGGGAATAAAACAAGAGACAAGA
AGGCATAAAAAGTTTTCTAGCCATCTAATATAGAGACCCGATCTGAGCCGGAGGACCGCACC
AAAGTTACTAAGAGCGAGAGCCCTAGTGCTCTCTCACATACAGATAAAGTCAAGTAAGTAAA
TCTGTTCAGGTAGGTTAAACAACTAGAGTAGCACAGTACCCCGAATAACAACAAAACAAGAA
ACTCCAAACACAAAAGGGTGACCAAGAGATGTTTAGTGGAAGAAAGAAAAATAATAAGAGCT
ATAAATAGACAGATAAGTAACAAAGATTCAATGAGACTAAGTGGTGAATTATCACACTGGTCT
TGTAAACCAGAGTTGGACAAGTTCCCTAAGTCTTCAAAAGGGTGAATCTCACACATCTACCTT
CCAAAGGTAGAATTCTTATTAAATTACCTTTTGACATTATAGTAGTTATACTAGCTATCAGTTC
CGCGATAATTTTTATCAATCATCCGCTATCGTTTACTCTCTGCCTTCTACTCCAAACAGTCCT
CATATGCGGGATATTGACGACTTTATCCCCTTGAGTCTCCCTTGTCTTATTTCTTATCTTCTT
GGGGGGGATTCTGGTGATGTTTTTATATGTTGCGTCACTGAGAGCAAATGAGAGATTCGTTAT
GGATAGATCTATCTTATTGACAGTAGCAGTAACTGTTATCACAACTCTAGTTCTCTGATCGGA
GATTAGAGTAACTAATCCCTCTAGTAAAAGGCTGCATCAAAATATAGACAGGGTATTGCATTG
GACAGGAAGCCCTCTCTTCCTTTCTCTCACTATTTATTTATTTTTAGCCCTTCTACTGATTGTA
GAATTTTTAAATATTAATAAAAAACCCTTGCGGTCTCTCTTATAATGGTATCTCTACGGAAGA
ATCACCCAGCTCTAAGTATTGCTAATTCTGCCCTTGTTGATCTACCTGTGCCCTCGAATATTT
CTATCTGATGGAACATAGGGTCATTGTTGGGGCTATGCCTGATCACTCAGATCATTACGGGC
CTATTCTTAGCTATGCATTACACAGCAGATGTTGATCTTGCATTCTCAAGTGTAGCCCACATC
TGTCGAGACGTAAATTATGGGTGGCTTCTTCGATCTTTACATGCTAATGGAGCATCTTTTTTT
TTTATTTGTGTGTACCTTCACATCGGACGGGGACTGTACTACGGATCATTTCGATATATAGAA
ACATGAAACACGGGAAT
>Eh1_scaffold-42
TAGAGGCTCTAAGGTAGGAAGTAAATATTGCTTCTAATGTGGACCTTATTGTTAATATTGTAA
CACACACAAACCTTTACCTGCACTTCCGTTTTCTTATAGTTCTCATTCTTATGCATTCTTCCA
TAAACTAAAAGTTTCGTAAAGTTTGATCCTGTCTAAGGGTTTAATCTTTCTAGGGGCAATATG
CAAATTGTTTCTTTCGGATTTATAAGTGCAGTTGTTAATATTAGTATTTACTGGCATAGAACTA
GTCAAAGAATGTTAGACAAGCCAATCTTGTGCCAGCAGCTGCGGTCACACAAGAATGTTGAA
TTTAACCTTTTAAGTTAAATTTCTTTGTTTTTTGGAATAAGAATAAGCCTTTAAAGGGTGAAAT
CTAAATATCTTATCCAGGTTTCTCTATAACTTGATCTGCATCACTAAAATTACTGGGATTAGA
TACCCCACTATTGTGCTTGCCCTATATCTTGGGTAGTATTCTAAAAACCCGAAGGATTTGGCG
GTTCTTAAACCTACTAGGGGAACCTGTCCTTTAATCGATAGTCCACGCACTGGTGTACTTTAT
CTAGTCTTCAGTATATATACCGCCGTCTAGGGGACTGTACTAACATCTCCTCTCGATAAAGGA
ATCTTTATAAGTCAGGTCAAGGTGTAGCTTATGGTAGAGTAGAGATGGGTTACAATAACTTGT
TACACGGAAGACGATGTTTAATTATTGTCTGAAGGTGGACTTAGAAGTAAACCTTTTAAATTT
GAAGTAGGCTCTTAGGAATGTACACATCGCCCGTCGCTCTCTTGTCTGAAGAGATAAGTCGT
AACAAAGTAGGTGTACTGGAAGGTATATCTAGACAGATTAGAAGGGAGCTTGAATAAGTTCC
TCACTTACACTGAGGAGATCCCGCCAGGGTCTTCTAAAAGCACAGAATGCCTGTTTTTTGTA
CGCAAATAATTGTATCAGGAGTAATAGTTATAAAACATTGGTTTGGCGGTTAGTATCGTGAGA
GAAATCATCTACATTATTAATAAGTTGTTGTATGTACCTTTTGTATCAGGGTTAATCAACTTTG
TATCTCTATGGAGTATTCCCGAAATTTTGTGAATTACCCCTTTTGATTATGCATGTATCATCAT

GCTAGCCAATTTAGGGGTCGTTTCTATAAGTCATTCGGACAAATATATCTGGTTGATCATCGA
TCCTTTTAGAGGAGGTAATATAGTTTTACTCAATTGATAGGGGATGAGCTCTATTAACAGTTA
AATATTTCTGTAATATCTATACTTTTTAAAGCTTAGAAGTAGCTGAAGTTTAGCAACTCGGGT
ATAAAGAAAAAGATTTAAATTATATTAATAATAAAGATGCTCATTGTTTTGTACTCATCTAAAA
TATGAATAATGATTAAATGAGTAAAATTAAAATTTTTAAAATGTAATTATTCACACTTATTTAA
AGACTTTTATAGTAGAAAAGGAACTCGGCAAAGAAGTCTCCGCCTGTTTAACAAAGACATGG
CCTTATAGTTTTATAAGGTCTAGCCTGCCCACTGATGATTTGAAGGGCCGTGGTATACTGACC
ATGCGAAGGTAGCATAATCATTTGCCCCTTAATTAGGGGCTAGAATGAAGGGTCGGACGAGG
GACTTCCTGTCTCTTTTATTAGCTTGAAATTAATCACTGGGTGAAAAAGCTCAGGTTATTTTG
AAGGACGATAAGACCCTATGGATCTTTATAAATAGACTTGAAGAGTTTGGTGGTTAGAATCTT
AGAGGATTTTTATTGTGTTGGGGCGACACTGAGAGGAGAACAACCCCTCAGCTTATTAAAAC
ACTTCTTTGTGGTTAAAGATCCTTGACTGAAATTAGACCAAGTTACCCTAGGGATAACAGCGT
TATATCTTTTGAGAGTTCAAATCGACAAAGATGATTACGACCTCGATGTTGGTTCAGGGAACC
TACAAGGTGCAGCAGCTTTGAGAGGAGGTCTGTTCGACCTTTAAACCCCTACGTGATCTGAG
TTCAGACCGGCGTAAGCCAGGTTAGTTTCTACCCTCAAAATACTTTTTCCTAGAGTAGTACGA
AAGGACCCTCTAGAATTTTACCATCTTGGCAGATAAATGCAATAGGCTTAGGACCTATCTATA
GAATATTTTCTAGCTGGTAAGCCTTGCTTATAATAATATTATCTGTTTTGTTACAAATTATTCT
AGTGTTGGTATCTGTCGCTTTCTTGACTCTCTTAGAACGTAAGATTTTAGGGTATATTCAACT
TCGTAAGGGTCCTAACAAAGTCGGATTTTTAGGCTTACTTCAACCCTTTGCCGATGGTGTCAA
GCTTTTTACTAAAGAGATATCCCACCCCAGGATGTCTAATACCCTCCCTTTTTACATCTCTCC
TGTCCTTAGTCTTTCTCTTTCTTTAGTTGGATGAACTTTAATCCCTTTTTACAGATACGCGTAC
GCCTACTCCTATTCAGTGGTCCTTTTCTTATGTGTTGTTAGATTAAGTGTGTACACTGTAATA
ACAGCTGGTTGATCCTCTAATTCTAAGTATTCTCTGCTAGGAGGTATACGGGCTGGTGCCCA
GACCATTTCTTACGAGGTCTCTCTTATTCTCGTCCTTCTATCTCCTCTTTTGATATGGGGGGC
CTACAGGTATCAATTTATTGCTCACATTTCCCTATACACAGGTCCCCTTCTCCTACTAATACT
TCCTCTTTCTTTAGCTTGACTTGTCACAATTCTAGCTGAAACAAACCGTACTCCTTTTGATTT
AGCTGAAGGGGAGTCTGAGCTTGTATCTGGGTTCAATACCGAGTATAGAAGGGTAGGTTTTG
CTCTTATCATGCTATCTGAATACGCGAGTATCCTTCTTATATCATTTATATTTATGCTTATCTT
CTCTGGTGCGGATATTTTAGTTTTTATCTTTGTGGTGTATGTGTTTCTCTGGTCTCGAGGGTC
TTATCCTCGTTACCGTTATGATCACCTTATATCCCTTTCTTGGAAAAGATTCCTTCCTTTGTCT
ATCAGGTTTATGCCCTTTTATCTCGGACTAAGCTATTTACTCTAATAGTTCAATAAGAAGGTT
TGGTTTCATACTTTTTATAGGGATGAATACTTTTGTATGCAGTAAAAAAAATACTACGGTAAA
GCACATTTTTATTAACCTTACTAGGTTCTTGGTAGCTTAATTTACCCTCTACTGTTTTCAAGAC
AGTCGCTTCTATCGGCCAAAGAACCACTTAATAAGGTTATCTAGCATTTTGAAAATTATAGGG
GTGATAACATAGAATCTGAAGTAGACTACAGTTAGAATCTGACCTGTTAAGATAAAGGGGTCT
TCCACGGGTC
>Eh6_scaffold-0
GAGTATACAGGAGAAGGAGAGGCCCCCTATCTCCTCTATCATCTTGACTAATAAGCTATTTCA
TGGGATAGCTTTAAAGAGTGACCCTATCTTCACATTGAAAATGTGATGTATTTATTATACTAT
CAAAGCAAACAAAAGATAGAGTTAAACTATCTCAAGGCAAAGTTAGAAGCTTTTCTTGGTTCC
TCTTCATTTTTTAATAGGGATAAACAAATCCTCTCTCTCCAGTAACAGTGGATTGCTTATTAG
CTTCTACTAAAGAAAGGGGCTCAAGGCCTAATTTTAGGTCGAAACTAAGGATGATACATCAAT

TCCTTTCCCGAGAGCAGACGCTTGTCTTCTCTTGATTGCAAATCAAGTATTTTAATTAAACTA
CGCTCCTATTTGATTCACCTAAGTGCCCCTTCTTCTCATTCGTATAAAACTCCTGTCACTAAA
ATGACAATAAAGAAAACAAAGACTGTGACTCTTGTTAATTCTAACCCTATCAAAGAATTAATT
GGCAGCAAAAGTGTGATTTCGACATCAAAGATAAGGAAAATAAGTGTGATGACAAAAAATCG
CAGTGAAAATGGCATTCGTGTTGAATTTTGGGGGTCAAACCCACATTCAAAAGGTGAGTTTTT
TTCTCGTTCCATAATTGCTTTTTTGTTAATCACTCTGCTGGCTAACAACATAATAGTAGAGAG
CATGATTAGAGAAATTCACAGTAGGGCAATCCAAACAATTACTTACTCCACATGTGGGCCTTC
TGATTGGAAGTCAGATATACTTATATACTAAGTAAGTTATTCACCTCATCAGTAGATAGAAAC
ATAGAGGAAGAGTCACACTACATCTACAAAATGCCAGTACCAAGCAGCTGCTTCAAATCCAA
AGTGGTGCTCTTGGGAGAAATGACATCGTAAATGACGTGCGAGACATACTATTAAAAATGAT
GAGCCAATAATGACATGGAGTCCATGGAATCCCGTTGCTACAAAAAAGGTGGACCCGTAAAC
TCTATCAGCGATAGAGAATGATGCCTCAATATATTCTACTCCTTGAAGAAACGAGAAGTATAC
CCCTAGAATAACAGTAAGGAGCAGTCCCTGTTTACACTGGTCAAAGTTGTTTTCTATCAATCC
ATGGTGAGCTCACGTAATAGTTACTCCTCTCGCCAAAAGAATTCTTGTGTTTAATAAGGGAAT
CTGGAAGGGATTAAACCCTTCAATGCCCACAGGTGGTCATACTGCTCCTACTTCTACATTGG
GCGAGAGTCTTCTGTGAAAGAAAGCTCAAAAAAATGATACAAAGAAAAAAATTTCAGACGCG
ATAAACAGAATTATGCCTCATCGCAGTCCTTTTCCTACTTTGGAAGAATGATGTCCTTGGAGT
GTTCTCTCACGTCTAATATCACGTCATCACTGCATTGATACGATGCATGCTCTCACAACTCCT
AGAATGAAAAGAGACGAGTTAAACTGGTGAAATCATTGGACAAGCCCTGTTGTCAGCCTAAA
AGCTGCTAAGGCTCTAACAATAGGTCACGGTCTTACGTTCACAATATGGTATGGATGGTTCA
GTTGAGTCATTATTCCCTGGCGTAGAGAGTAGATAAGGTAGCAAACACGTATGCTTGAATAAT
TGCTACAGAAAACTCAAGTACTAGAAGCATTACTTGTCTTATTAACACAACCAGTACCGGCAA
CACTCCTGCCCCAATTGCTTGGTTGCCTAGAAGGGTGAGAAGTAAATGTCCAGCAATTATAT
TGGCAGCGAGTCGTACTGAAAGGGTGATAGGCCGGATAAAATTACTCACCAACTCAATTACT
ACCATAAAAGGTATTAAAACAGGAGGAGTACCTAGAGGTACTAAATGTGCTAGTGCGTGTTT
ACTCTCTTTAATTCATCCATGCAGCAGGAAAGATAGCCAGAGAGGAAGTGCTAGAGCTAGAG
TAATGGCTAGATGACTAGTTGCAGTAAATAGGTACGGGAATATCCCCATCAAGTTATTGAATA
GAATGAATGAAAAAACTCTCACCAGTAAAAGTGGTGACCCCCTTTTATTGGGTCCTATCAAAA
GAGAAAATTCCTGGTCTAATTTTATCATGACGGTATCCCATATCACCTGAACTCGTCTAGGTA
TAGCCCAGAATGCTCATGGTAGTATAATAAACGTTAATGTCATGGACAGCCAGTTGGTTAAAA
GAGAGGATGTAGGATCAAACACTGAAAATAGGTTAGTTATCATACTCAACTATTTACTCTTAC
TCGGGGAGTATTGATATTCCTTTTAAGGGCGATGGGTTGGTCGATGAAATAAGTCATAGTCAT
TACTAAAATAATGATTAGAACTGAAGTGACTAATAGGGTTGTTCACGGGAGAGGAGCTATTTG
AGGTATAAGAAATTACTCTTTGAGTACCTGGAGAATGACACTCTCCTATTCTTGTAAAACTCA
ATTTCTGCCATAAGAGGTAGTAACACCCTACTAAGGCTTAAAAAGCCTTTGCTCTCTGTCAGC
CATCTTATGAAATTTGTCTCTGTAATCAATTGAGGAATCTTTTAGTGTCTACCGATTCAAGAA
CAATTGGTATAAACCTGTGGCCTGCTCCACAGATTTCCGAGCATTGCCCATAAAAAATGCCT
GGTATGTTAATAAGTATTCCCGATTGGTTTAAGCGCCCTGGGATAGCATCTATCTTGATCCCA
ATAGAAGGAAGAGCTCATGAATGAAGAACATCATCGGATGTAATTATAAGCCGAATTGGCTG
ATTAAAAGGGAGAGGTACTCGGTTGTCTACATCCAGAAGGCGGAATAGTCCTTGTTCTAGGT
CTTGAGTAGGTACTATATAAGAGTCAAACTGGAGATCATTAAAATCTGAGTATTCATACCCCC
AGTATCATTGGTGCCCTGTTACTTTAATAGTTAGGGCTGGGTTTTGTACTTCATCAATTAAGT

AAAGAAGCCGAATAGAGGGCAAAGCGATAAAAATCAGGATTACTGCAGGGAGAACAGTTCAA
ATGGTCTCAATTATCTGTCCGTCTAGCAAATAGCGGTGTACGTAATTATTAAAAAATAAAGAC
ACTATAAAAAATCCTACCAAGGACGTGATCAATACTACAATTAGTAGAGCGTGATCATGAAAA
AATACTAACTGTTCTATCAAAGGAGACACCCCATTTTGTAGTCCTAACTGAAATCACTGAGAC
ATTTTCTAAAGGGGAAACTCCCTTGGATGGAGCTTAAGTCCACTGCATATAATTTCTGCCACA
TTAGAAAGAAGAGACGAGAGTGAGTTCTTCGTAGCTATGGTCAGCAGGAGGATGGGGGTGAT
GTCACTCAATGTTAGATCTTAGGTTAAGGGAGAACACATTTGCCCGCTTTAAGGTAAAGGCCT
CTCATAAGCAAAAGATAAACCCCAGGGTCCCTATAAAAGAGATAATTCTTCCGACTGAAGAG
ACCATATTCCAAGAAGCATAGCTGTCTGGGTAATCGGCGTATCGCCGTGGTATCCCTGCTAG
TCCAAGAAAGTGTTGAGGAAAGAACGTAACATTTACTCCCAAGAATATAATTATGAAATGAAC
TTTTAAGTAAGTAGTATTAAGGGATACTCCTGTTAGAAGTGGGAACCAGTGTGTGAATCCCCC
TAAAATGGCAAATACAGCTCCCATTGAAAGAACATAGTGAAAATGTGCTACAACGTAATAGGT
GTCGTGTAAAATAATATCGACCCTAGAGTTAGCCAATACCACCCCTGTTAGTCCACCCACAG
TGAATAAGAATACGAATCCTAGGGCTCACATAAGTGAAGGTCTTATAGTAAACCGAGTACCG
TGCAACGTTCCAATTCATCTAAAGATTTTAATTCCTGTAGGGATCGCAATAATTATAGTGGCA
GCTGTGAAATAAGCCCGTGTATCAACATCTATACCTACTGTGAATATGTGATGCGCCCAGAC
CACGAATCCCAGAACTCCGATTGCCAGTATAGCATAGATTATACCTAAAGTGCCGAATGCTT
CTTTTTTCCCTCTTTCCTGCCTGATAATATGGGACACTATACCAAACCCTGGCAGAATTAAAA
TATACACCTCAGGGTGTCCAAAAAATCAAAAGAGATGTTGATAAAGGATAGGATCCCCCCCT
CCTGCAGGGTCGAAGAACGATGTGTTCAAATTTCGATCCGTAAGTAGCATTGTAATAGCTCC
TGCCAAGACGGGTAAAGATAAAAGAAGAAGAACTGCTGTGATTCCAACTGACCACACAAAAA
GTGGTATTCGGTCTAATGACATTGATTGGGGACGCATGTTCAAGATAGTTGTAATGAAATTAA
CTGCCCCCAGAATCGAAGAGATCCCTGCGAGATGGAGAGAAAAGATAGAAAGGTCTACAGAC
GGTCCTGCATGAGCGATTCCTGACGAGAGAGGAGGATATACTGTCCACCCGGTTCCTGCTCC
CCTTTCTACTAAAGACCCCGCTAACAGGAGAGTTAGTGATGGTGGTAGTATTCAAAATCTTAA
GTTGTTAAGACGCGGAAAAGCTATGTCTGGTGCTCCTAATATGATTGGGACTAGTCAGTTAC
CAAACCCCCCAATGAGAATAGGTATCACTATAAAGAAAATTATGACAAAAGCATGGGCGGTT
ACAATAACATTGTAAAGTTGCTCATCTCCTAACAGGGATCCTGGCTGACCTAGTTCGGCCCG
AATTAGTATTCTAAGAGAAGTGCCTACCATTCCTGATCAAGCTCCGAATAAAAAATAAAGCGT
GCCGATATCCTTGTGATTAGTGGAATAGAGTCATCGTTGCAAAAGTAGAGTGGCTGATTTAG
GTGTAAGGCTGTAAACCTTGACAAGGAGTCGTCCTCCCTCTACTAAGTCTTATAGTTTAAAAG
AATAGAAGATTGCAAATCTTTAGGTGCCTTTGGCTGAGATTTTATATCTTGGTGTATCGTGCA
CTTAAGGTTTTGATCCTTAGAGTAGAGTTCTAATCTCTAGGTATAAAGGTATAAAGACAGGGT
GCCTGATTAAAGGGTTACGTTGATACTGTAAATATGCATAAATGCCCCTGTCTAGAACCTAAG
ATTTTCTTATGGTCAGCTTTGAAGGCTACTAGTTTATATTAACTTAAGGTTCTAAAGCATAGC
CACAGGTACTATTCAGTGAAATCCAATTACTAATCACGTCCAGAAATCACCCCGAGAAACAG
ACAAAGGTGATAAAATAGGAATTATTATAATTTTAAAATAAAAGAATAGAGACATGCATGTCG
ATGAAACAAGTCTGGTCAGGATCACCAGAGAAGGCACTGAGTTTTCCAAAGTTATTCACTTAA
TTATAAACCCCAGAAGAGGTGGAAGTCCTCCCAAAGAAAGAAGACAAGAGCCAAGAGCTCTC
TTTATCCCTGCCGTCATGGGGCTATTTATCTGCCTGACAAAATACGCACCCAATCTCTGAAAG
ATCAGAACTAGAGTTGCGGTAATCAGTGTATATACCCAAAAATAAGACATTAGTAGTAGTAGA
CTCTTTATATTTATAAACACTCACGCCAAGTGTGTGATAGAAGAGTAGGTAATAAGTAAGCGC

AAATTGGATTGCAGGATACCTCCTAAAGCCCCAATGAGTGCTGAGAGTATCACGATAGCCCC
CCTATCCAATGTAAGCATTAGAAGTCCAAGTAAAGGCCCTAGTTTTTGAAAGGTTAATAAAAA
AAATAGAGAGACTCATGATATATTTGGTGTTACTGCTGGGACTCATAGGTGAAGAGGGGCTG
CTCCGATTTTTAAAAGAAGCCCACAGATCAGACCAACATGGAGTCATGGGTTGATGACCCCC
GATAAAAACAGAAGGGAACCTAGAGATTGTACTAAAAAATATTTTAATCTTATTTCCTTGTCC
CTCCATACCAAAATAGGAATAAATCTAAGGGCGTTTAATTCTAGGGCCAATCATAGCCCTAGT
CAAGATTCAGTTGAGAATATCAAAATATAGGATAGAGCTAAAATTAGTCATTTTATTATCAAG
AATTTGGTGTTTCTTTAAAGAGGAGATGACTCTATCGGCAGGGTATGAACCTGATAGCTTATT
GTTTAGCTTACCTCTTTGGAATAGTAAACTACGGGTATAAACTAATTTGATGTTTTATACTAGT
TAGACAGAGTAAAGTCTTCAGTAATTAATTTCTCCATGAAAAATAAAATACTAAATTTCATCA
AGAACAATAAAATTGATCTTCAATTAAGGTAAAGGTCATCGAAGATGCTGCTGTTGGAAGGCA
GAGGAATGAAGCCCCATCCTTTAAAAGATTGTTAAGCCTATCTTGGTCTAAGACTGGAGCGA
TAGCCTCGTAGTAAATCTAATTGAATTCCTATTTATCTTCATATTTTTATCCCTAATCTTATTT
TTGTCCTCAAATAAGAGGACACATTCCTATGACCCAGTCTTTTTGTTTCTGCTTTGAGTATTG
GTTGTTACTGGGCTTGTTTTTATTTCTTGGACTGATAGAGTACTTTCTTCTCTTATTACTACTG
AACTTTCCTTTCCTTTTTTTGCCCAATCTCTGAGACCTCAGTGTTTAGATCCTACTTTTGAAG
GTATAGGCCTGCCAGATTCTCCTCCCTGTCTTTTTAATAATACTAGTCATAACGGGAATCTCT
CCTCGGGAGAAAGTCGTACCCCTTCCCCCGCTTTAGTGGGAGAAGATTGATCTGGGACAACC
CATAGATTTATTTTTAATAATTTCCTCATGGCCAAAGAAAGGTTGCTCACTCAATATCACCAA
AATCAGTCTATCCTAAGAATTAATATTAATAAAAACACTTCATCTATTACACGGGATTTTGTA
GACGCAAAGATAGACGATTTCCCCCTTTCGATATGACGTCTTGGGGAAAGACACCTTATCAC
TGTAGTGGGTATAAGGTCACACCCCGCAATGGTAGAATACGGGGATGACTCTTGTGTCCCAC
GTTTAGTCTTTGAAGAGATCGCAGGAGCTTTTCATGTTGCTTTTGGGACTAATGTCTCCACAT
TTACTCCTCCTATGCCTATAGAAGAAGCAAAGACTTTGTTAGATAGGTACCAAACTCAAGGTG
ATATACTAGGTTTTCATGCAATTCTTCACTCTGCTCACTTAGAGGCAATTTCTGTTAATGACG
TGTGCGTAGGAGCTCTTATGTGTGATGAATGGGACAATCTCCACGCTCAACTAATCCTAAGG
TTCAGAGATAACTCAGAACATCTGGTTCTAGAAAATCGGGGTATCGCTGTATCTATTCTTCGT
CAAGAGTTTATGTCTATAGTACCCTCGGGTACTGGAGTAGAGGATCTAAGAAAGGAGGAGAT
ATTCGGGGTATTGAATTCGTGCATTTGGGTGATTAGAAGGATACCGGGATCCTCGTATAGAA
ATCATGAATTTGATTATTGCTGTTCTGTAATTGGCCCTCTCATAGGAAAACAACTCTCTTATG
CCCCTATAGTAGAGTACCAGACAGCCTACGATATTTTATCTAAATCTCTTATTCTAATAGACC
GTCTTAGCCTTGCTGGGATGGTCACCTGCATAGATCATATCCTGGGTCGTGAGTAAATAGTC
CCATACTCTATAGCATCTTACTGTAGATAGGACATATAGGCTATCTGTAGAGAGGGGAGGTAT
GGGGG
>Eh6_scaffold-328332
CCCCCATACCTCCCCTCTCTACAGATAGCTATGTATGTCCCTAGAAAAGAATTCACCCTAAC
ATCTTCAGTGTCATGCTCTTATTAAGCTATAGGAACTAAACAAATCAAATCCCAGTAAAAGAC
ACTAAAAGGAGAAGTTTAAAACCTGTTGACATAAAGATTTCTCTCAAAGAACTCAAAAAAAGA
ATCCCTGTCTTCTGAGCACCGGGGCCCAAATATTCTACTCAACCGTGATCTCCCTGGCTGTA
AATGATGTCTCCTCTTTTAAGCAATGGCACCCTAAGAATAGATGTTAAATAAGGAAGGAAAGT
TATAGTGGTTAAGAATCAAACACACGCCCCTTACTCGCTGTTATCCTAAAACCTGCTAAAAC
CCCCACCATAATACACAAAAGCAAAGTTAATTTTTCTATAGAAGAAACTATAAGACTTTCTGT

ACCTAAAAAAAATCAATAAAACAAGGATCCCCCGAATAAGGCCCCTCTAAACAAAACCTTAA
GAGGAGTAGAGTAGTTCCCTTCATCCCCTCTAATAAAAGAAACTCAGTTAAAGACTGAAGAA
GTAAGTGCCACTGAAATAAGACGAAAGGAATAGCCGCAGGTTAAGAGAGCAGCAGCAAGGAT
AGCGAGGGAAGGGAATATTAGTAATTCTCTAGTAGAACTCTCAATAATTGTGTCTTTAGAATA
AAACCCAGCTAAAAAAGGAAACCCCATTAAGCTAAAGGAAGCAGCCCTAAGAGCTAAGCAAG
AAAACGGAAGAAAGAGGGAAACTCCCCCCAAACGACGAATATCCTGAGTACCCCCAGAGGA
ATGGATAATAACCCCAGAACACATGAACAAGAGAGCCTTAAACAAGGCATGAGTAAATAAAT
GAAAGTAGCATAGAAGAGGGCAGCCTACCCCCAAAGAGAACATCATCACACCTAGTTGTCTT
AGAGTAGAGAGGGCAATAACTCGTTTGAGGTCGTACTCCGTTATAGCCACTAGACCGGAGAG
AAGTGCAGTAATAGAACCGAGTAAAATTAAAAGGAGATTGCCTGAATGGGAAAAGCACGGAG
AAAGACGAACTATCAAAAAGATGCCTGCTGTTACAAGAGTAGACGAGTGGACAAGTGAAGAG
ACAGGGGTAGGAGCTGCTATAGCCGCGGGTAATCAAGCTGAAAAAGGAATTTGAGCCCTTTT
AGTTAAAGACGCCAATAAAAGAAGAATTAAAACTGTATAAGAGGACTCGGCAGAAATAAAAC
TGTAATCCCAAGAACCAAGAGAGTAAGTAAGACCTAGAGATCAGAGAATAAGAATATCTCCT
ACTCGGTTACTTAGAGCAGTAATTATTCCTCTTGAAGAAGATTTAGGATTGGAGTAAAAAAGA
ATCAAAGCATAAGAAGTAACTCCTAGCCCATCTCACCCTAATAAAAGACTCACACCGTCTGA
AGAAATGATAAGAAAAGCTATGGAGAGTACAAAAAACAACAATAGGTACTTAAACCGGTCAT
AAAATACCTCCCCTCCCATGTAATAAGAAGAATACATGAGAACTTGAGAGGAGATTAACCTTA
CAAGGAAAAGAAAAGAAAGAGAAATTCAATCAAAGTGTACTTGATAAATAAAGGAGAAGAAA
GAAACTGACACGAGATAAATAGAATCACTCAGATGGGATAAGAAGGATAACCCTAAAAAGGT
ACATCCCAAAACAGAGAGAAAAAATCTATAAATGTAGTAAGTCAAAGTTCTTGGAGATATTCA
CCGCTAGAATCACAATCTAGAATCCTTCTTTAGACTACAAGAACATAAAGTGACTAACAAAAG
GAGGGGGATGAAATGGAGAGCTCTTACTAGAAGTTCATTAATGTCTGAATGTTGAAGACCAG
TCTTTATTAGAGTCTCTCCGTGAATAGTTCAAGAGTATAGGAATAAGCAGTAGCAAGCTGACA
AGAATGAAATTAAGCCTGCTAAAACAAGGAATGATAAGTGAAGACCGCCGGAAGCGATAAAC
AAGTAAAGTTCCGAAAACAAGTTTAGAGAAGGAGGAACCCCCATATTAAAGATGATAAGGAG
AAATCATCATAAAGCAATAAAGGGAGAAAAAACAATAATACCACGAGTTATTAAGAAAGAACG
AGAGCCAAAATAAGTGTAAATTAGATAACTTAGGTAAAAAAGACCTGAGGAACAAAGACCAT
GGGCGACTATCAACAGGAGGGAAGCTCTATTCCCATAACTTCCCATAAATAGCATCCCTAAG
ATAATAAATGCTATATGAGCAACCCTAGAATAAGCGATTAAGGACTTCACATCAGTTTGACGG
AGTGAGAGCAGACATCTTAAAACGCCTCCTCATCCGGCTAAGAGACAGATATAGCCGAGTAG
ACTTACCCCTATTAAAGGTTGCACTAAGAATAGCCCATACCCGCCTAGCTTTAATAAGATAGC
AGCTAAAACCATTGAGCCAGTGACAGGAGCTTCTACATGAGCTTTAGGCAATCAGACATGAG
TAAAGTATATGGGCAACTTAACTAAAAAGGCGAAGACTATAAAGAACACAAAGAGTTTGTCTG
CACTGGAGGGAAGAAAAAAAGAGGTTGAAGTCTGGTACTCTATATCTAGGCCCAAGAGAATA
AAGAGGAGAGGCAGTGAAGCTAAGAGGGTATAGAATAAAAAATAAAGGGATGCGGGGAGAC
GCTCGGGCTGGTAACCTCAGCCCATAATTAAGAGAAGGGTGGGGATTAGAGAGGACTCAAAA
AAAATATAAAAGGAGATAAGAGAATTGGAAGTGAATGTGAGGACTAACAGCAAAAAGAGAAG
AATAATTAAAATGCTGTAGGTACGCTTATAGGAAGGGGATCAGCATATAATCATTAAGAAGAA
AATTCACCCCCTAAGGATAAGAAGATAGTGAGATCAACTGAATTTGAAAGAAGAACACAAGA
TAAGAAATGGGGAATAAAACAAGAGACAAGAAGGCATAAAAAGTTTTCTAGCCATCTAATATA
GAGACCCGATCTGAGCCGGAGGACCGCACCAAAGTTACTAAGAGCGAGAGCCCTAGTGCTC

TCTCACATACAGATAAAGTCAAGTAAGTAAATCTGTTCAGGTAGGTTAAACAACTAGAGTAGC
ACAGTACCCCGAATAACAACAAAACAAGAAACTCCAAACACAAAAGGGTGACCAAGAGATGT
TTAGTGGAAGAAAGAAAAATAATAAGAGCTATAAATAGACAGATAAGTAACAAAGATTCAATG
AGACTAAGTGGTGAATTATCACACTGGTCTTGTAAACCAGAGTTGGACAAGTTCCCTAAGTCT
TCAAAAGGGTGAATCTCACACATCTACCTTCCAAAGGTAGAATTCTTATTAAATTACCTTTTG
ACATTATAGTAGTTATACTAGCTATCAGTTCCGCGATAATTTTTATCAATCATCCGCTATCGT
TTACTCTCTGCCTTCTACTCCAAACAGTCCTCATATGCGGGATATTGACGACTTTATCCCCTT
GAGTCTCCCTTGTCTTATTTCTTATCTTCTTGGGGGGGATTCTGGTGATGTTTTTATATGTTG
CGTCACTGAGAGCAAATGAGAGATTCGTTATGGATAGATCTATCTTATTGACAGTAGCAGTAA
CTGTTATCACAACTCTAGTTCTCTGATCGGAGATTAGAGTAACTAATCCCTCTAGTAAAAGGC
TGCATCAAAATATAGACAGGGTATTGCATTGGACAGGAAGCCCTCTCTTCCTTTCTCTCACTA
TTTATTTATTTTTAGCCCTTCTACTGATTGTAGAATTTTTAAATGTTAATAAAAAACCCTTGCG
GTCTCTCTTATAATGGTATCTCTACGGAAGAATCACCCAGCTCTAAGTATTGCTAATTCTGCC
CTTGTTGATCTACCTGTGCCCTCGAATATTTCTATCTGATGGAACATAGGGTCATTGTTGGGG
CTATGCCTGATCACTCAGATCATTACGGGCCTATTCTTAGCTATGCATTACACAGCAGATGTT
GATCTTGCATTCTCAAGTGTAGCCCACATCTGTCGAGACGTAAATTATGGGTGGCTTCTTCGA
TCTTTACATGCTAATGGAGCATCTTTTTTTTTTATTTGTGTGTACCTTCACATCGGACGGGGA
CTGTACTACGGATCATTTCGATATATAGAAACATGAAACACGGGAATCGCCCTATTATTTATT
CTCATGGGGACAGCTTTTTTAGGATATGTGCTACCCTGAGGGCAGATATCTTTTTGGGGGGC
TACAGTAATTACTAACCTTGTGTCTGCAATCCCTTACATGGGAGTGGATATTGTCCAGTGGGT
GTGAGGCGGATTTGCTGTAGATAACCCTACTTTAACACGATTCTTTACTTTACATTTCTTGTT
GCCATTTCTACTCGCCGGAACAACAATAATCCACTTATTGTTTTTGCACCAGACTGGATCAGG
AAATCCTTTGGGCATTAATAGAAGTATTGATAAAGTACCATTTCACCCTTATTTTTCCATTAA
GGATATTGTAGGGTTCTTAGTGTTTTTAACAGCACTAGTAATTTTAACTCTTTTAGAACCTAAT
GTTATAGGAGACCCAGACAACTTTATCCCCGCCAATCCTCTAGTCACCCCTGTCCATATTCA
GCCAGAATGGTACTTCCTTTTTGCCTACGCCATTTTACGGTCTATTCCTAACAAACTAGGGGG
GGTACTCGCACTTGTGATCTCAATCCTAATTCTGGCGAGAGTGCCTGTTACCTTTAACCCCA
AATTTCGGGGTATTAGATTTTACCCATTAGTGCAGACTTTATTCTGGTCTTTAATCTCAACTG
TGCTCCTTCTCACATGAATTGGAGCACGACCCGTGGAAGACCCCTTTATCTTAACAGGTCAG
ATTCTAACTGTAGTCTACTTCAGATTCTATGTTATCACCCCTATAATTTTCAAAATGCTAGATA
ACCTTATTAAGTGGTTCTTTGGCCGATAGAAGCGACTGTCTTGAAAACAGTAGAGGGTAAATT
AAGCTACCAAGAACCTAGTAAGGTTAATAAAAATGTGCTTTACCGTAGTATTTTTTTTACTGC
ATACAAAAGTATTCATCCCTATAAAAAGTATGAAACCAAACCTTCTTATTGAACTATTAGAGT
AAATAGCTTAGTCCGAGATAAAAGGGCATAAACCTGATAGACAAAGGAAGGAATCTTTTCCA
AGAAAGGGATATAAGGTGATCATAACGGTAACGAGGATAAGACCCTCGAGACCAGAGAAACA
CATACACCACAAAGATAAAAACTAAAATATCCGCACCAGAGAAGATAAGCATAAATATAAATG
ATATAAGAAGGATACTCGCGTATTCAGATAGCATGATAAGAGCAAAACCTACCCTTCTATACT
CGGTATTGAACCCAGATACAAGCTCAGACTCCCCTTCAGCTAAATCAAAAGGAGTACGGTTT
GTTTCAGCTAGAATTGTGACAAGTCAAGCTAAAGAAAGAGGAAGTATTAGTAGGAGAAGGGG
ACCTGTGTATAGGGAAATGTGAGCAATAAATTGATACCTGTAGGCCCCCCATATCAAAAGAG
GAGATAGAAGGACGAGAATAAGAGAGACCTCGTAAGAAATGGTCTGGGCACCAGCCCGTATA
CCTCCTAGCAGAGAATACTTAGAATTAGAGGATCAACCAGCTGTTATTACAGTGTACACACTT

AATCTAACAACACATAAGAAAAGGACCACTGAATAGGAGTAGGCGTACGCGTATCTGTAAAA
AGGGATTAAAGTTCATCCAACTAAAGAAAGAGAAAGACTAAGGACAGGAGAGATGTAAAAAG
GGAGGGTATTAGACATCCTGGGGTGGGATATCTCTTTAGTAAAAAGCTTGACACCATCGGCA
AAGGGTTGAAGTAAGCCTAAAAATCCGACTTTGTTAGGACCCTTACGAAGTTGAATATACCCT
AAAATCTTACGTTCTAAGAGAGTCAAGAAAGCGACAGATACCAACACTAGAATAATTTGTAAC
AAAACAGATAATATTATTATAAGCAAGGCTTACCAGCTAGAAAATATTCTATAGATAGGTCCT
AAGCCTATTGCATTTATCTGCCAAGATGGTAAAATTCTAGAGGGTCCTTTCGTACTACTCTAG
GAAAAAGTATTTTGAGGGTAGAAACTAACCTGGCTTACGCCGGTCTGAACTCAGATCACGTA
GGGGTTTAAAGGTCGAACAGACCTCCTCTCAAAGCTGCTGCACCTTGTAGGTTCCCTGAACC
AACATCGAGGTCGTAATCATCTTTGTCGATTTGAACTCTCAAAAGATATAACGCTGTTATCCC
TAGGGTAACTTGGTCTAATTTCAGTCAAGGATCTTTAACCACAAAGAAGTGTTTTAATAAGCT
GAGGGGTTGTTCTCCTCTCAGTGTCGCCCCAACACAATAAAAATCCTCTAAGATTCTAACCA
CCAAACTCTTCAAGTCTATTTATAAAGATCCATAGGGTCTTATCGTCCTTCAAAATAACCTGA
GCTTTTTCACCCAGTGATTAATTTCAAGCTAATAAAAGAGACAGGAAGTCCCTCGTCCGACC
CTTCATTCTAGCCCCTAATTAAGGGGCAAATGATTATGCTACCTTCGCATGGTCAGTATACCA
CGGCCCTTCAAATCATCAGTGGGCAGGCTAGACCTTATAAAACTATAAGGCCATGTCTTTGTT
AAACAGGCGGAGACTTCTTTGCCGAGTTCCTTTTCTACTATAAAAGTCTTTAAATAAGTGTGA
ATAATTACATTTTAAAAATTTTAATTTTACTCATTTAATCATTATTCATATTTTAGATGAGTACA
AAACAATGAGCATCTTTATTATTAATATAATTTAAATCTTTTTCTTTATACCCGAGTTGCTAAA
CTTCAGCTACTTCTAAGCTTTAAAAAGTATAGATATTACAGAAATATTTAACTGTTAATAGAG
CTCATCCCCTATCAATTGAGTAAAACTATATTACCTCCTCTAAAAGGATCGATGATCAACCAG
ATATATTTGTCCGAATGACTTATAGAAACGACCCCTAAATTGGCTAGCATGATGATACATGCA
TAATCAAAAGGGGTAATTCACAAAATTTCGGGAATACTCCATAGAGATACAAAGTTGATTAAC
CCTGATACAAAAGGTACATACAACAACTTATTAATAATGTAGATGATTTCTCTCACGATACTA
ACCGCCAAACCAATGTTTTATAACTATTACTCCTGATACAATTATTTGCGTACAAAAAACAGG
CATTCTGTGCTTTTAGAAGACCCTGGCGGGATCTCCTCAGTGTAAGTGAGGAACTTATTCAA
GCTCCCTTCTAATCTGTCTAGATATACCTTCCAGTACACCTACTTTGTTACGACTTATCTCTT
CAGACAAGAGAGCGACGGGCGATGTGTACATTCCTAAGAGCCTACTTCAAATTTAAAAGGTT
TACTTCTAAGTCCACCTTCAGACAATAATTAAACATCGTCTTCCGTGTAACAAGTTATTGTAA
CCCATCTCTACTCTACCATAAGCTACACCTTGACCTGACTTATAAAGATTCCTTTATCGAGAG
GAGATGTTAGTACAGTCCCCTAGACGGCGGTATATATACTGAAGACTAGATAAAGTACACCA
GTGCGTGGACTATCGATTAAAGGACAGGTTCCCCTAGTAGGTTTAAGAACCGCCAAATCCTT
CGGGTTTTTAGAATACTACCCAAGATATAGGGCAAGCACAATAGTGGGGTATCTAATCCCAG
TAATTTTAGTGATGCAGATCAAGTTATAGAGAAACCTGGATAAGATATTTAGATTTCACCCTT
TAAAGGCTTATTCTTATTCCAAAAAACAAAGAAATTTAACTTAAAAGGTTAAATTCAACATTCT
TGTGTGACCGCAGCTGCTGGCACAAGATTGGCTTGTCTAACATTCTTTGACTAGTTCTATGCC
AGTAAATACTAATATTAACAACTGCACTTATAAATCCGAAAGAAACAATTTGCATATTGCCCC
TAGAAAGATTAAACCCTTAGACAGGATCAAACTTTACGAAACTTTTAGTTTATGGAAGAATGC
ATAAGAATGAGAACTATAAGAAAACGGAAGTGCAGGTAAAGGTTTGTGTGTGTTACAATATTA
ACAATAAGGTCCACATTAGAAGCAATATTTACTTCCTACCTTAGAGCCTCTAGTCTCTCCTC
>Eu17_scaffold-96
TGCTCTATTAGAGGGGATACCCCGTTTTGTAGTCCTAACTGAAATCATTGAGACATTTTCTAA

AGGGGAAGTTTCCCTTGGATGGAGCTTAAGTCCATTGCATTTAATTTCTGCCACATTAGAAAG
AAGAGATGAGAGTTAGTTCCTCGTATCTATGGTCGGCAGGAGGATGAGGGTGATGTCATTCA
ATGTTAGACCTTAAATTAAGAGAAAAGAGGTTTGCTCGTTTTAAGGTAAAGGCCTCTCATAGA
CAAAAGATAAACCCGAGGGTCCCCAAAAAAGAGATTATTCTACCTACTGAAGAGACCATGTT
TCAAGAAGCATAACTGTCCGGGTAATCGGCGTATCGTCGCGGTATTCCTGCTAGCCCAAGAA
AATGTTGAGGAAAGAAAGTAATGTTTACTCCCAAAAACATGATTATGAAATGAATTTTTAAAT
AAGTTGTGTTGAGAGATACTCCTGTTAGAAGCGGGAATCAGTGGGTAAATCCCCCTAAAATG
GCAAATACAGCTCCCATTGAAAGGACATAGTGAAAATGTGCTACTACGTAGTAGGTATCGTG
CAAAATAATGTCAACTCTTGAGTTAGCTAATACCACTCCTGTTAGTCCTCCTACAGTAAATAA
GAACACGAACCCTAAGGCTCATATAAGAGAAGGTCTCATTGTAAACCGGGTCCCATGTAATG
TCCCAATCCACCTAAAGATTTTGATCCCTGTGGGGATCGCAATAATTATAGTTGCAGCTGTGA
AGTAGGCTCGTGTGTCAACATCTATGCCCACCGTAAATATATGATGCGCCCATACTACAAAT
CCTAATACCCCGATTGCCAACATGGCATAGATTATCCCCAAAGTACCAAATGCTTCCTTTTTT
CCTCTCTCTTGTCTAATAATGTGAGATACTATACCAAATCCAGGGAGAATTAAGATATACACC
TCAGGATGCCCAAAAAATCAAAAGAGATGTTGGTAAAGAATAGGATCTCCCCCTCCCGCAGG
ATCGAAGAACGACGTGTTCAGATTACGATCCGTCAATAGTATAGTGATGGCTCCTGCTAAGA
CAGGCAGAGACAGCAGGAGAAGAACCGCTGTAATTCCAACTGATCATACAAAAAGAGGTATC
CGGTCCAGCGACATTGACTGGGGTCGCATGTTTAAAATAGTAGTAATGAAATTAACTGCTCCT
AGGATCGAAGAAATCCCTGCAAGATGAAGAGAAAAGATAGAGAGATCTACAGATGGTCCCGC
ATGGGCGATTCCCGATGAGAGAGGGGGATACACCGTCCATCCGGTCCCTGCTCCCCTTTCTA
CTAGGGATCCTGCTAATAGGAGAGTCAAAGATGGCGGCAGTATTCAAAATCTTAAGTTGTTAA
GGCGTGGGAAGGCTATATCTGGTGCTCCCAGTATGATTGGCACTAATCAATTACCAAATCCC
CCAATTAGGATGGGCATCACTATAAAGAAAATCATGACAAAAGCATGGGCGGTTACAATTAC
GTTGTAAAGTTGTTCATCCCCTAACAAGGATCCTGGCTGGCCTAACTCAGCTCGAATCAACA
TCCTAAGAGAAGTGCCTACCATACCTGATCAAGCCCCAAATAAAAAATAAAGTGTACCAATG
TCTTTATGATTAGTAGAATAGAGTCATCGTTGCAAAAGTAGAGTGGCTGATTTAGGTGTAAGG
CTGTAAACCTTAATAAGGAGTTGAACTTCCTCTGCTAAGTCTTATAGTTTAAAAGAATAAAAG
ACTGCAAATCTTTAGGTGCTCTTAGCTAAGACTTTATATCTTGGTGTATTGTGCACTTAAGGT
TTTGATCCTTAGGGTAGAGTCCTTATCTCTAGGTATAAAGATAGGGTGCCTGATTAAAGGGTT
ACGTTGATACTGTAAACATGCATAAATGCCCCTGTCTAGAACCTAAGATTCTCTTATTATCAG
CTTTGAAGGCTACCAGTTTGTGTTAACTTAAGGCTCTAAAGTATAACCACAGGTGTAACCCAA
TGAAATCCCACCACTCATCACCCCCAAAAATTATTACGAAAGTTGGATACAGGTGATAAAAC
AGGGATCATTATAATTTTAAAGTAAAAAAATAGAGATAAGCACGTGGACGAGATAAGTCCGG
CTAAAACCACTAGCGACGGTATTGAATTTTCTAGAGTCATTCATTTAATTATGAACCCTAGAA
GAGGGGGAAGTCCCCCCAAAGAAAGGAGACAAGAACTAAGAGCTCTTTTTATTCCTATTGAT
ATGGGGGTGTTCATTTGCCTGACGAAAGATACACCCAGCCTCTGTAGAGTCAATACTAAAGT
TAGAGTAATTAATGTGTAAACTGAAAAATAAGATACTAGTAACGATATACTTCTTATATTTATA
AATACTCAAGCTAAGTGAGCAATAGAGGAATAAGTGATAAGTAAGCGCAAGTTTGATTGTAAG
ATACCCCCCAAGGCTCCAATAAGAGCCGAAAACACCACAATGATTGTCCTATTAAATGTGAG
TATTAAAAGACCTAGCAAGGGCCCCAATTTTTGAAAGGTCAGTAAAAAAAGAACGGAAACTC
ACGTTATATTGGGTGTTACTGCCGGTACCCACAGATGAAGAGGAGCTACTCCAATTTTTAGAA
GTAGTCCACAAATTACTCCAATATAAAGGAGTGGGTTAATAACTCCCGACAGGAATAGAAGG

GATCCTAATGATTGAACTAGAAAATACTTCAAACTTACTTCTTTATCCTTCGATACCAAAATA
GGGATAAACCTCAGGGCATTTAACTCTAGGGCTAGCCACAACCCTAGCCAAGACTCAGTAGA
GAATATTATGATGTAAGATAGGATTAAAATTAATCACTTTATTATAAAGAAGACAACTACTTTT
CTTTAAAGAGGAGATGACTCTATCGGTAGGGTATGAACCTAATAGCTTGGTATTTAGCTTACC
TCTTTGAAAGAACGAATTATAAATATAAGTTAAATTTGTGCTTAGTCATGACTAGTTAAAATCA
ATTCTACGGGTATTAACTCCATATAAAGACATAAAATACCAAATTCCGTCAAGAACAAGAAAA
CTGGGGGAATCAATTAAGATGTGTTGAGTAAGAGACGGTTTTTTTATGCCCCTGCTATGAGTG
CCAAGTAGTCTATGGTGTTTTATTCAAGTCTTTGTCCCCATTGATCATCTTAGTTTTGCTGGG
AGGCTAGTCATATAAGTTTATCTTAGATAACGAGTAGATTGTGCAAACAACCCAATTGGTAAC
ATATATGGCTATCTGTAGAGAGGG
>Eu17_scaffold-31
GACAGTTATGCTTCTTGAAACATGGTCTCTTCAGTAGGTAGAATAATCTCTTTTTTGGGGACC
CTCGGGTTTATCTTTTGTCTATGAGAGGCCTTTACCTTAAAACGAGCAAACCTCTTTTCTCTT
AATTTAAGGTCTAACATTGAATGACATCACCCTCATCCTCCTGCCGACCATAGATACGAGGA
ACTAACTCTCATCTCTTCTTTCTAATGTGGCAGAAATTAAATGCAATGGACTTAAGCTCCATC
CAAGGGAAACTTCCCCTTTAGAAAATGTCTCAATGATTTCAGTTAGGACTACAAAACGGGGTA
TCCCCTCTAATAGAGCAATTAGTATTTTTCATGATCATGCATTGCTAATCGTAGTGTTAATTA
CATCCTTAGTAGGATTTTTTATAGCGTCTCTGTTTTTTAATAATTACGTACATCGGTATTTACT
GGATGGGCAAATGATTGAAACTATTTGGACTGTGTTACCTGCAGTGATCCTAATTTTTATTGC
TTTGCCTTCTATCCGACTCCTTTACTTAATTGATGAAGTACAAAACCCGGCTCTAACCATTAA
AGTAACAGGACATCAATGGTACTGAGGATATGAGTACTCAGATTTTAATGACTTACAATTCGA
CTCTTACATAGTGCCTACTCAAGACCTGGAACAAGGATTATTCCGCCTCTTAGATGTTGATAA
CCGAGTCCCCCTCCCTTTTAATCAACCAATTCGCCTTATAATTACATCCGACGATGTTCTGCA
TTCGTGGGCTCTTCCTTCCATTGGAATCAAGATAGATGCTATCCCTGGACGACTGAACCAAT
CAGGGATACTTATTAATATACCAGGCATTTTCTACGGACAGTGCTCAGAGATCTGTGGAGCG
GGCCATAGATTTATACCAATCGTTCTAGAATCAGTAAATACTAGAAGGTTCCTCAATTGATTG
CAAGGACAAATTTCATAGGATGGCTGATAGAGAGCGAAGGCTTTTTAAGCCTTAGTAGGGTG
TTACTACCTCTTATGATAGGAATTGAGTTTATCAGAATAGGAGAGTGTCATTCTTCAGGTACT
CACAGAGTAATTTCTTATCCCACAAATAGCTCCACTTCCGTGAATGACTCTATTAATTACTGC
AGTTATATTTGTAGCTCTAGTGATAACTATAATTTACTTCATTATCCAGCCTGTAACCCTTAAA
AATAAGGTTAGTACTCCGCGATTAAGAGTAAATGATTGAGCATGATAAGTAATCTATTTTCGG
TATTTGACCCTACATCCTCCCTTTTAACAAACTGACTGTCCATAGCGTTAACATTTTTTATGC
TACCCTGAGCATTCTGAGCTATACCCACACGAATTCAAATAATTTGAGGTACTGTTATAACCA
AGCTAGACCAAGAGTTTTCTCTTTTGTTAGGACCTAATAAAAGAGGGTCACCACTTTTACTCG
TAAGAGTTTTTTCATTTATTCTTTTCAACAATTTAATGGGGATATTCCCCTATCTTTTTACTGC
AACCAGTCATCTGGCTGTCACTCTTGCCCTAGCACTGCCTTTATGACTATCATTCTTACTATA
TGGCTGAATTAAAGAGAGTAAACACGCGTTAGCACATTTAGTACCGCTAGGAACTCCTCCTG
TTCTGATGCCATTTATGGTGGTGATTGAGCTAGTAAGTAATTTTATCCGGCCTGTCACCCTTT
CAGTACGACTGGCCGCCAATATAATTGCTGGTCACCTACTTCTTACCCTCCTGGGTAACCAA
GCAATTGGAGCAAGGACACTAACGATAGCAATCGTATTAGTAAGACAAGTAATACTTCTGGC
ACTTGAATTTTCTGTAGCAATTATTCAAGCATATGTATTTGCCACCTTATCTACACTCTACGC
TAGAGAGTAATGACTCAATTAAACCACCCGTATCATATTGTTAACATCAGACCTTGGCCGATT

GTTAGAGCCTTAGCAGCTTTTACGTTAACTACCGGGCTTGTTCAATGATTTCATCAGTTTAAC
TCGTCTCTTTTTATTTTAGGAACACTAAGAGCATGCATTGTATCGATCCAGTGGTGACGTGAT
ATTAGCCGTGAGAGAACACTCCAGGGGCACCATTCTTCTAAGGTAGGGACAGGATTACGATG
AGGGATAATTCTGTTTATCGCATCTGAAATTTTTTTCTTTGTATCGTTTTTTTGGGCTTTCTTT
CATAGCAGACTCTCTCCCAATGTAGAAGTAGGGGCAGTATGACCCCCAATCGGGATTGAAGG
ATTTAACCCCTTCCAAATCCCTTTATTAAATACTAGGATTCTCTTGGCCAGAGGGGTGACTAT
CACATGAGCCCACCACGGGCTAATAGAAAATAATTTTGACCAGTGTAAACAGGGACTGATAA
TAACTGTTGCTCTCGGGGTATATTTCTCATTCCTTCAAGGAGTGGAGTATATCGAGGCATCAT
TTTCTATCGCTGACAGCGTTTACGGGTCCACCTTTTTTGTAGCGACAGGATTTCATGGACTTC
ATGTAATTATCGGGTCATCATTCCTACTAGTATGCCTCACACGGCACTTACGATGCCACTTCT
CCCAAGAACATCACTTTGGATTCGAAGCTGCTGCTTGATACTGACATTTTGTGGATGTAGTGT
GGCTTTTCTTGTACATCTCTATCTACTGATGAGGTGAGTAACTTGTTTAGTATATAAGTATAT
CTGACTTCCAATCAGAAGGTCCATGTATGGAGTAAGTAATTATTAGAATTGTCCTGCTCTGAA
TTTCACTGATGATGCTGTCTAGAATCATACTATTAGCCAGTAGAATAATTAGAAAAAAGACAG
TAATAGAACGAGAAAAGAACTCACCCTTTGAGTGCGGATTTGACCCGCAAAATTCAACACGA
ATACCGTTCTCGCTGCGATTCTTCGTTATCACACTCATTTTCCTTATTTTCGATGTAGAAATC
ACACTTCTACTCCCAATCAATTCTCTAACGGGGCTAGAAATTATGAGGGTCACGATTCTTGTA
TTCTTTATTATTATTTTAGTAGCAGGAGTCTTATACGAATGAGAAGAGGGGGCGCTCAGATGA
ATTAAGTAGGAGCATAGTTTAACTAAAATACTTGATTTGCAATCAAGAGATAACAAGTGTTTG
CTCTCAGGAAAGGAATTGATTTATCATTCTTAGTTTCGACCTAAAATTAGGCTTTTAGCCCCT
TTCTTTAATAGAAGCTATTGAGCAATCCACTGTTACTGGAGAGAAAGGATCTATTTATCCCTA
TTAAAAGACAAAGAAGAGCCACGGAAAGCTTCTAACTTTACCTTGAGGTAGTTTAAATCTACC
TTTTGTCTGCTTTAATAGTATAAACAAATACGTCACATTTTCAATGTGTAGATAGAGTCACTCT
TTATAGCTGTCTAAGAGACAAATTTATAGAGTCAGAGATAAAGAAAAAAGGAAGTTCGGGGG
CCTCTCCTTCTCCTGTATACTCTCA
>Eu17_scaffold-12
CGTACTCGAATAAGGGAACCATAGGTTTCCTTAATGAGGAGAGACTAGGGGCTCTCAGTAAG
GAAGTAAAGACTGCTTGTAACGTGGACCTTGTTCATAATATTACAGTAATCATCAACTAATAT
TTATATCTCTTTTAATCCTTTATTAGAGTTTTATTTTGGTCGTCTACAAACTGTAAATTTCGTA
AAGTTTGATCCTGTCTAGTAGTCTAATTTTTCCAGAGACAATATGCATATTCTTTCATCTGGA
GTTATAAGTGCAGTTGTTAATATTAGAGTTTATCACTATAGAACTAGTCAAAGAATGTTAGAC
AAGCCAACCTTGTGCCAGCAGCTGCGGTCATACAAGGGTGTTGAGTTAGATTATTTAAGTTA
AATTTCTTTCTTTTTTGGAGTAAAAATAACCTTTTTAAGGGTGAAATCTAAATATTTTATTTAA
ACTTCTTTATAATTTGATCTGTAGCATCTATACCACTGGGATTAGATACCCCACTACTTTGCT
TGCAATATACCTTGGGTAGTATTCTAGAAACCTAAAGGATTTGGCGGTTTTTAAACCTACTAG
GGGAACCTGTCCTTTAATCGATAGTCCACGCGCTGGCACACTTTATTTTGTCTTCAGTATATA
TACCGCCGTCTAGGGGACTGTACTAACATTTCCTCTTAATAAGGGAATCTTTATAAGTCAGGT
CAAGGTGTAGCTTATGGTAAAGTAGAGATGGGTTACAATAACTCGTTACACGAAAGATGATCT
TTAATTATTATCTGAAGGTGGACTTAGAAGTAAATCTTTTCAATTTGAAGTAGGCTCTTAGGA
ATGTACACATCGCCCGTCGCTCTCTTGTCTGAAGAGATAAGTCGTAACAAAGTAGTCATACT
GGAAGGTATGTCTAGACAGACTAGAAGGGAGCTTGAATAAGTTCCTCACTTACACTGAGGAG
ATCCCGTTAGGGCCTTCTAAAGATATGGCATGCCTGTTTTTTGTATTCAAACAATTGTAAGAG

GAGTAATAGACATAAAACATCGTATCGGCGTTTAGTATCGTGAGAGAAATTATTTATATTGTT
AATAAGTTGTTGTACGTACCTTTTGTATCAGGGTTGGTCAATTTTACTTCTTTAAAGAGTATTC
CCGAAATTTTGTGAATTACCCTTCTTGATTGTGTATGTTTCATCATGCTAGTTAATTCTAGGGT
CGTTTCTATAAGTCATTCGGACAAATATATCTGGTTGATCATCTGTCCTTTTAGAGGAGGTAA
TATTCTTTACTTAGTTGATAGGGGATGAGCTCTATTAATAGTTAGATACTTTTATAATTTTTAT
ACTCTTTAAAGCTTAGAAGTAGCTGAAGTTTAGCAACTTGGGTATGAATAAAAAGATTTAAGA
AGTATTAAAACTATGGATGCTTCGTGTTTTACATTAATCTCAGACACAGATAATGATTAAATG
AGTAAAATTAAAAGTCCTAAAGTATAACCATTTATACTAAGTCAAATTCTTTTAAAGCAAGAA
AGGAACTCGGCAAAGAAATCTCCGCCTGTTTAACAAAGACATGGCCTTATAGTTTTGTAAGGT
CTAGCCTGCCCACTGATGATTTGAAGGGCCGTGGTATACTGACCATGCGAAGGTAGCATAAT
CATTTGCCCCTTAATTAGGGGCTAGAATGAAGGGCTGGACGAGGGACTTCCTGTCTCTTTTG
TTAACTTGAAATTAATCACTGGGTGAAAAAGCTCAGGTTATCTTGAAGGACGATAAGACCCTA
TGGATCTTTATAAATTTACTTGAAGAGTTTGGCTGTTAGAATCTTAGAGAAGTTTTATTGTGTT
GGGGCGACACTGAGAGGAGAATAATCCCTCAGTTTACTGAAACACATCTTTGTGGTTAAAGA
TCCTTGATTGAAATTAGATTAAGTTACCCTAGGGATAACAGCGTTATATCTTTTGAGAGTTCA
TATCGACAAAGATGATTACGACCTCGATGTTGGTTCAGGGAACCTACAAGGTGCAGCAGTTT
TGAGAGGAGGTCTGTTCGACCTTTAAACCCCTACGTGATCTGAGTTCAGACCGGCGTAAGCC
AGGTTAGTTTCTACCCTCAAAATATTTCATTTTAGAGTAGTACGAAAGGACCCTCTATAATTT
TACCATCTTGGCAGACAAATGCAATAGGCTTAGGACCTATCTATAGAACACTTTCTAGCTGGT
AAGCTTTGCTTATAATATTATTATCCGTCTTATTACAGATTATTCTCGTGTTGGTATCTGTCGC
TTTCTTGACTCTTCTAGAACGTAAAGTTCTAGGCTATATTCAACTTCGTAAGGGCCCTAATAA
AGTTGGATTTCTAGGTCTTCTTCAGCCTTTTGCTGATGGTATTAAGCTCTTTACTAAGGAGAT
GTCTCACCCCAGCATATCAAATACTCTACCCTTCTATATTTCTCCCGTTCTCAGTCTCTCCCT
TTCCTTAATCAGATGAACCCTCATTCCTTTTTATGGGTATATATACACTTACTCTTATTCGGTG
GTTCTCTTTTTGTGTGTCGTGAGATTAAGCGTGTATACCGTAATAACAGCCGGCTGGTCATCC
AATTCTAAGTATTCCCTTCTAGGAGGCATGCGGGCCGGTGCCCAAACCATCTCTTATGAGGT
TTCACTTATTCTCGTTTTACTATCCCCTCTTTTAATGTGAGGGGTTTATAGGTATCAATCTATT
GTTCATATCTCTTCTTACACAGGTTCTCTTGTATTGTTAATACTCCCCCTTTCTTTGGCTTGG
CTTGTAACAATTCTGGCTGAAACAAACCGTACTCCTTTTGATCTAGCTGAAGGGGAGTCTGA
GCTAGTTTCTGGCTTTAACACAGAGTACAGAAGGGTAGGTTTTGCCCTCATCATGTTGGCTG
AATACGCCAGTATTCTTCTTATATCTTTTATATTTATACTCCTCTTTTCCGGAGTTAACACACT
AGTCTTCATTTTTGTGGTTTATGTGTTTCTTTGGTCCCGGGGTTCTTACCCTCGTTACCGTTA
TGATCATCTTATAGCCCTTTCTTGGAAAAGATTTCTTCCTTTGTCTATTAGATTTATACCCCTT
TATCTTGGTATAAGTTACTTACTTTAATGTGTTAATAATGAGACCCGTTTTTTACTTCTTTTAT
AAAGGTCAAAATACCTTTATGCAGTAAAAAAAATACTACGATAAAGCAAATTTTTATTTGTCT
CATCAGATTCCTGGTGGCTTAATTCACCCTTTACTGTTTTCAAGACAGTCACCTCTATCGGTC
AAAGAACCACCTAATCATATTATCTAGTTGTTTAAAAATAATAGGGGTGATAATGTAATAACT
AAAGTACACCACGGTCAAAATTTGGCCTGTTAAGATAAAGGGGTCTTCTACGGGTCGTGCTC
CGATTCATGTAAGAAGGAGAACAGTTGAGATCAAAGATCAAAATAAAGTCTGTACTAACGGG
TAAAATCTAATCCCTCGAAATTTAGGGCTGAAAGTAATAGGTACTCTGGCTAGGATTAAGATT
GAAACTACCAGTGCGAGTACCCCCCCCAATTTATTCGGAATAGATCGTAAAATAGCGTAGGC
GAAAAGGAAGTATCATTCTGGTTGAATATGAACAGGAGTGACGAGAGGATTGGCGGGGATGA

AGTTGTCTGGGTCTCCTATAATATTAGGTTCCAAAAGGGTTAAAATTACTAGCGCTGTTAGAA
ACACTAAGAATCCTACAATATCTTTAATAGAGAAATAAGGGTGAAATGGTACTTTATCAATAT
TTCTATTAATCCCCAAAGGATTTCCTGACCCAGTTTGATGTAAAAATAGTAAGTGGACTATAG
TTATTCCGGCAAGTAGGAATGGTAGAACGAAATGAAAAGTAAAGAATCGTGTCAAAGTAGGG
TTATCTACAGCAAATCCTCCTCATACTCATTGAACAATGTCTACTCCTATGTAAGGAATTGCA
GACACGAGGTTAGTAATAACTGTAGCTCCCCAAAAAGATATTTGTCCCCAAGGTAGGACATA
GCCTAAAAAAGCTGTTCCTATCAGGATAAATAATAGTGCGATCCCAGTGTTTCATGTCTCTAT
GTATCGGAATGATCCGTAGTAAAGTCCTCGCCCGATGTGGAGATATACACAAATGAAAAAAA
AAGATGCTCCATTAGCATGTAATGATCGAAGGAGTCATCCATAATTAACATCCCGACAAATGT
GGGCCACACTTGAGAATGCAAGTTCAACGTCCGCTGTGTAATGTATTGCTAAAAATAGGCCC
GTAACGATTTGGGTGATTAGACATAGCCCCAACAGTGACCCCATGTTTCACCAAATAGAGAT
ATTTGAGGGAACTGGTAGATCAACGAGAGCAGAATTAGCAATTCTTAGAGCTGGGTGATTCT
TTCGTAGAGATACCATTATAAGAGTGATCGTAGTGGCTTTTTGTTAACGTTCAAAAACTCTAC
AATAAGGAGAAGGGCTAAAAATAGGTAGATAGTCAGAGAAAGATAGAGAGGCCCCCTACTTC
AGTGTAGCACCCTGTCTATATTCTGATAAGATCTTTTATCAGCAGGATATATTACTTCAATTTT
TGACCAGAGAACTGCGGTCATAATAGCGGTTACAACTATCATTACTAAGGTGGCTCTATCTAT
GATGAACCTTTCATTTGCTCTCAGTGATGCAACATACAGGAACATTACTAGAATTCCCCCTAA
GAAGATAAGAAACAAAATAAGAGAGATTCAGGGGGAAAGAGGTGTTAATGTAACACATACAA
CGATTGTCTGGAGTAGGAGACAGAGAGTAAACGATAAAGGGTGGTTGATGAAAATTATTGCG
GAACTAATCGTTAGTATAATTAATATAATATCAAAAGGTAATTTAATAAGAATTCTACCTTTGG
AAGGTAGGCGTGTGATATTTCACCCTCTTGAAGACTCAGGGGACTTCCCAACTCTGGTTTAC
AAGACCAGTATGATAATTCACCACTTAGTCTTATTGAATTTCTACTTCTCGTTTGTTTATTGAT
GGCCCTGATTATCTTTCTTTCCTCCACTAAGCATCTTTTGGTTATTCTCTTATGTTTGGAGTTT
CTTATCTTATTATTATTTGTATTACTGTGCTATTCTAGTTATCTGACTTATCTAAATAGATTTA
CTTATTTGACTTTATCCGTATGTGAGAGAGCATTAGGTCTTTCACTTTTAGTAACTTTGGTCC
GGTCTTCTGGTTCAGATCAGGTATCTATATTAGATGGCTAGAAAATTTTTTACACCCTCTTGT
ATTTTATTCTATTCTTCATTTTTTATCTTGTGTTGTTCTTTTAAGTTCAGCTGATCCCATTATCT
CCTTGTTCTTAGAGGTTGAATCTTCTATCTAATAATTATATGTTGGTCTCCTTCCTACAAATAT
ACTTACACAACTCTTATTGTTCTTCTCTTCTTACTGCTAGCTTTAACTTTCACTTCTAATTCTC
TTATTTCTTTTTATATCTTCTTTGAGTCCTCTTTAATCCCCACCTTTCTTCTAATTATAGGTTG
AGGCTACCAGCCTGAACGTCTCCCTGCATCTCTTTATTTTCTTTTTTATACCCTTTTGGCTTC
ATTACCTCTTCTTTTCATCCTTTTAGGGTTAGAGATAGAATACCAGACCTCCCTTGTGTTTCT
TCTACCCTCTAGCTCAGATAAACTCTTTGTGCTTTTTACAATTCTTGCCTTTCTAGTTAAGTTG
CCCATGTACTTTACTCACATCTGATTGCCCAAAGCTCATGTAGAAGCTCCCGTTACGGGCTC
GATGGTCCTAGCGGCTATTCTACTAAAGCTAGGGGGGTATGGCCTGTTTCTAGTGCAACCTC
TTATAGGAGTAAGTTTACTCGGCTATCTTTGTCTTCTAGCGGGATGAGGGGGTGTTTTAAGAT
GCTTGCTCTCTCTCCGTCAAACTGATGTTAAATCATTGATCGCTTACTCAAGGGTTGCTCATA
TGGCACTTATCATTCTAGGAATGTTATTTATGGGCATTTATGGAAACAGCGCCTCTCTTCTCT
TAATAATCGCTCACGGCCTTTGTTCTTCCGGTCTTTTTTATCTAAGCTATCTCACGTATGTTT
GTTTCGGTTCTCGTTCTTTTCTAATAACTCGTGGCATCCTTGTCTTTTCTCCTTTTGTCACCTT
ATGATGGTTCCTTCTCATTATCTTCAATATGGGAGTCCCCCCCTCCCTAAATTTGTTCTCGGA
ACTCTATTTGTTTGTTGCCTCTAGGGGATTACATCTGTTGTGTCTTGTTCTAGCAGGCTTAAT

TTCATTTCTGTCAGCCTGTTACTGTTTGTTTCTTTACTCCTGAACAATTCATGGGGAGACTCT
GGTAAAAACTGGCCTTCAGTACTCTCACGCTAGTGAACTCTTAGTAAGAACTCTCCATTTTAT
CCCTCTTCTTCTATTAGTGACCCTTTGTTCTTGTAGTCTAAAGGAGGATTCTAGATTGTGATT
CTAGCGGTGAATATCTCCAAGGACTTTGACTTACTACATTTATAGGTTCTTTCTTTTCCTCAT
GGGGTGCATTTTTATGGGCTTATCTCTTTTATCCTACCTGAGGGACTCAATTTATCTGGTTTC
AGTGTCCTTTTTTTCTTTCGTTTATCAAGTACACTTTGACTGAATTTCTCTTTCATTTCTTTTT
CTCGTAAGGCTAATCTCTTCCCAAGTTCTAATGTACTCTACTTATTACATAGGAGGCGAGGCA
TTTTATGATCGTTTTAAGTATCTATTATTACTTTTTGTCTCTTCTATGGCCTTTCTTATTATCTC
TTCTGATGGTCTTAGTCTCCTATTGGGATGAGACGGGCTGGGAGTTACTTCTTATGCCTTAAT
TCTTTTTTACTCTAATTCTAAGTCTTCTTCAAGGGGGATAATTACCGCCTTAAGTAACCGGGT
TGGGGATATTCTTATTCTCTGATCTCTAGGTCTGAACTATGCTCTAGGTTCTTGAGACTACAG
GTTTATTTCCGTTGAATCTTCTTACGTAGTAATATTCCTCCTCTTATTAGCGTCTTTAACCAAA
AGAGCTCAAATTCCTTTCTCAGCTTGATTACCCGCGGCCATAGCAGCTCCTACCCCAGTTTC
CTCACTTGTACACTCATCAACTCTTGTAACAGCAGGTGTCTTTCTAATAATTCGTCTTTCTCC
CTGTTTTTCTTACACGGGGAACCTGCTTTTAATTTTACTTGGTTCTATTACCGCACTTCTCTC
TGGTCTCGTGGCCATAGCGGAGTATGACCTGAAACGAGTCATTGCTCTTTCTACACTGAGTC
AACTCGGCGTAATAATATTCTCCCTGGGGGTGGGCTGTCCTCTCCTATGTTATTTTCATCTAT
TTACTCATGCTCTGTTTAAAGCTCTCTTATTTATGTGTTCTGGAGTAATCATCCATTCTTCCG
GGGGTACTCAAGATATTCGTCGTCTAGGAGGAGTCTCTCTTTTCCTCCCGTTTTCTTGCTTAG
CTCTTAGGGCTGCCTCTTTTAGTTTAATGGGGTTTCCGTTTTTGGCCGGGTTCTACTCTAAGG
ATATGATTATTGAGAGTTCTACTAGAGAGTTATTGATATTCCCATCTCTCGCTATTCTCGCTG
CTGCTCTTCTGACCTGCGGCTATTCCTTCCGTCTCATTTCTGTAGCTTTAACTTCTTCCGCCT
GTAACTGAGTTTCCTTCATTAGTGGAGATGAAGGAGATTACGTCGTTCCACTCAGGGTCTTAT
ACAGGGGGGCTTTGTTTGGGGGAGTATTGTTTTATTGATCCTTTTTAGGTGTAGAGAGCCTTA
TGGTCTCTCCTATGGAAAAATTGACTTTGCTTGCATTTATTAG
>Eu36_scaffold-34
CCCCATACCTCCCCTCTCTACAGATAGCCATATATGTTACCAATTGGGTTGTTTGCACAATCT
ACTCGTTATCTAAGATAAACTTATATGACTAGCCTCCCAGCAAAACTAAGATGATCAATGGGG
ACAAAGACTTGAATAAAACACCATAGACTACTTGGCACTCATAGCAGGGGCATAAAAAAACC
GTCTCTTACTCAACACATCTTAATTGATTCCCCCAGTTTTCTTGTTCTTGACGGAATTTGGTA
TTTTATGTCTTTATATGGAGTTAATACCCGTAGAATTGATTTTAACTAGTCATGACTAAGCAC
AAATTTAACTTATATTTATAATTCGTTCTTTCAAAGAGGTAAGCTAAATACCAAGCTATTAGGT
TCATACCCTACCGATAGAGTCATCTCCTCTTTAAAGAAAAGTAGTTGTCTTCTTTATAATAAA
GTGATTAATTTTAATCCTATCTTACATCATAATATTCTCTACTGAGTCTTGGCTAGGGTTGTG
GCTAGCCCTAGAGTTAAATGCCCTGAGGTTTATCCCTATTTTGGTATCGAAGGATAAAGAAGT
AAGTTTGAAGTATTTTCTAGTTCAATCATTAGGATCCCTTCTATTCCTGTCGGGAGTTATTAA
CCCACTCCTTTATATTGGAGTAATTTGTGGACTACTTCTAAAAATTGGAGTAGCTCCTCTTCA
TCTGTGGGTACCGGCAGTAACACCCAATATAACGTGAGTTTCCGTTCTTTTTTTACTGACCTT
TCAAAAATTGGGGCCCTTGCTAGGTCTTTTAATACTCACATTTAATAGGACAATCATTGTGGT
GTTTTCGGCTCTTATTGGAGCCTTGGGGGGTATCTTACAATCAAACTTGCGCTTACTTATCAC
TTATTCCTCTATTGCTCACTTAGCTTGAGTATTTATAAATATAAGAAGTATATCGTTACTAGTA
TCTTATTTTTCAGTTTACACATTAATTACTCTAACTTTAGTATTGACTCTACAGAGGCTGGGTG

TATCTTTCGTCAGGCAAATGAACACCCCCATATCAATAGGAATAAAAAGAGCTCTTAGTTCTT
GTCTCCTTTCTTTGGGGGGACTTCCCCCTCTTCTAGGGTTCATAATTAAATGAATGACTCTAG
AAAATTCAATACCGTCGCTAGTGGTTTTAGCCGGACTTATCTCGTCCACGTGCTTATCTCTAT
TTTTTTACTTTAAAATTATAATGATCCCTGTTTTATCACCTGTATCCAACTTTCGTAATAATTT
TTGGGGGTGATGAGTGGTGGGATTTCATTGGGTTACACCTGTGGTTATACTTTAGAGCCTTAA
GTTAACACAAACTGGTAGCCTTCAAAGCTGATAATAAGAGAATCTTAGGTTCTAGACAGGGG
CATTTATGCATGTTTACAGTATCAACGTAACCCTTTAATCAGGCACCCTATCTTTATACCTAG
AGATAAGGACTCTACCCTAAGGATCAAAACCTTAAGTGCACAATACACCAAGATATAAAGTCT
TAGCTAAGAGCACCTAAAGATTTGCAGTCTTTTATTCTTTTAAACTATAAGACTTAGCAGAGG
AAGTTCAACTCCTTATTAAGGTTTACAGCCTTACACCTAAATCAGCCACTCTACTTTTGCAAC
GATGACTCTATTCTACTAATCATAAAGACATTGGTACACTTTATTTTTTATTTGGGGCTTGATC
AGGTATGGTAGGCACTTCTCTTAGGATGTTGATTCGAGCTGAGTTAGGCCAGCCAGGATCCT
TGTTAGGGGATGAACAACTTTACAACGTAATTGTAACCGCCCATGCTTTTGTCATGATTTTCT
TTATAGTGATGCCCATCCTAATTGGGGGATTTGGTAATTGATTAGTGCCAATCATACTGGGAG
CACCAGATATAGCCTTCCCACGCCTTAACAACTTAAGATTTTGAATACTGCCGCCATCTTTGA
CTCTCCTATTAGCAGGATCCCTAGTAGAAAGGGGAGCAGGGACCGGATGGACGGTGTATCCC
CCTCTCTCATCGGGAATCGCCCATGCGGGACCATCTGTAGATCTCTCTATCTTTTCTCTTCAT
CTTGCAGGGATTTCTTCGATCCTAGGAGCAGTTAATTTCATTACTACTATTTTAAACATGCGA
CCCCAGTCAATGTCGCTGGACCGGATACCTCTTTTTGTATGATCAGTTGGAATTACAGCGGT
TCTTCTCCTGCTGTCTCTGCCTGTCTTAGCAGGAGCCATCACTATACTATTGACGGATCGTAA
TCTGAACACGTCGTTCTTCGATCCTGCGGGAGGGGGAGATCCTATTCTTTACCAACATCTCT
TTTGATTTTTTGGGCATCCTGAGGTGTATATCTTAATTCTCCCTGGATTTGGTATAGTATCTC
ACATTATTAGACAAGAGAGAGGAAAAAAGGAAGCATTTGGTACTTTGGGGATAATCTATGCC
ATGTTGGCAATCGGGGTATTAGGATTTGTAGTATGGGCGCATCATATATTTACGGTGGGCATA
GATGTTGACACACGAGCCTACTTCACAGCTGCAACTATAATTATTGCGATCCCCACAGGGAT
CAAAATCTTTAGGTGGATTGGGACATTACATGGGACCCGGTTTACAATGAGACCTTCTCTTAT
ATGAGCCTTAGGGTTCGTGTTCTTATTTACTGTAGGAGGACTAACAGGAGTGGTATTAGCTAA
CTCAAGAGTTGACATTATTTTGCACGATACCTACTACGTAGTAGCACATTTTCACTATGTCCT
TTCAATGGGAGCTGTATTTGCCATTTTAGGGGGATTTACCCACTGATTCCCGCTTCTAACAGG
AGTATCTCTCAACACAACTTATTTAAAAATTCATTTCATAATCATGTTTTTGGGAGTAAACATT
ACTTTCTTTCCTCAACATTTTCTTGGGCTAGCAGGAATACCGCGACGATACGCCGATTACCC
GGACAGTTATGCTTCTTGAAACATGGTCTCTTCAGTAGGTAGAATAATCTCTTTTTTGGGGAC
CCTCGGGTTTATCTTTTGTCTATGAGAGGCCTTTACCTTAAAACGAGCAAACCTCTTTTCTCT
TAATTTAAGGTCTAACATTGAATGACATCACCCTCATCCTCCTGCCGACCATAGATACGAGG
AACTAACTCTCATCTCTTCTTTCTAATGTGGCAGAAATTAAATGCAATGGACTTAAGCTCCAT
CCAAGGGAAACTTCCCCTTTAGAAAATGTCTCAATGATTTCAGTTAGGACTACAAAACGGGG
TATCCCCTCTAATAGAGCAATTAGTATTTTTTCATGATCATGCATTGCTAATCGTAGTGTTAAT
TACATCCTTAGTAGGATTTTTTATAGCGTCTCTGTTTTTTAATAATTACGTACATCGGTATTTA
CTGGATGGGCAAATGATTGAAACTATTTGGACTGTGTTACCTGCAGTGATCCTAATTTTTATT
GCTTTGCCTTCTATCCGACTCCTTTACTTAATTGATGAAGTACAAAACCCGGCTCTAACCATT
AAAGTAACAGGACATCAATGGTACTGAGGATATGAGTACTCAGATTTTAATGACTTACAATTC
GACTCTTACATAGTGCCTACTCAAGACCTGGAACAAGGATTATTCCGCCTCTTAGATGTTGAT

AACCGAGTCCCCCTCCCTTTTAATCAACCAATTCGCCTTATAATTACATCCGACGATGTTCTG
CATTCGTGGGCTCTTCCTTCCATTGGAATCAAGATAGATGCTATCCCTGGACGACTGAACCA
ATCAGGGATACTTATTAATATACCAGGCATTTTCTACGGACAGTGCTCAGAGATCTGTGGAGC
GGGCCATAGATTTATACCAATCGTTCTAGAATCAGTAAATACTAGAAGGTTCCTCAATTGATT
GCAAGGACAAATTTCATAGGATGGCTGATAGAGAGCGAAGGCTTTTTAAGCCTTAGTAGGGT
GTTACTACCTCTTATGATAGGAATTGAGTTTATCAGAATAGGAGAGTGTCATTCTTCAGGTAC
TCACAGAGTAATTTCTTATCCCACAAATAGCTCCACTTCCGTGAATGACTCTATTAATTACTG
CAGTTATATTTGTAGCTCTAGTGATAACTATAATTTACTTCATTATCCAGCCTGTAACCCTTAA
AAATAAGGTTAGTACTCCGCGATTAAGAGTAAATGATTGAGCATGATAAGTAATCTATTTTCG
GTATTTGACCCTACATCCTCCCTTTTAACAAACTGACTGTCCATAGCGTTAACATTTTTTATG
CTACCCTGAGCATTCTGAGCTATACCCGCACGAATTCAAATAATTTGAGGTACTGTTATAACC
AAGCTAGACCAAGAGTTTTCTCTTTTGTTAGGACCTAATAAAAGAGGGTCACCACTTTTACTC
GTAAGAGTTTTTTCATTTATTCTTTTCAACAATTTAATGGGGATATTCCCCTATCTTTTTACTG
CAACCAGTCATCTGGCTGTCACTCTTGCCCTAGCACTGCCTTTATGACTATCATTCTTACTAT
ATGGCTGAATTAAAGAGAGTAAACACGCGTTAGCACATTTAGTACCGCTAGGAACTCCTCCT
GTTCTGATGCCATTTATGGTGGTGATTGAGCTAGTAAGTAATTTTATCCGGCCTGTCACCCTT
TCAGTACGACTGGCCGCCAATATAATTGCTGGTCACCTACTTCTTACCCTCCTGGGTAACCA
AGCAATTGGAGCAAGGACACTAACGATAGCAATCGTATTAGTAAGACAAGTAATACTTCTGG
TACTTGAATTTTCTGTAGCAATTATTCAAGCATATGTATTTGCCACCTTATCTACACTCTACG
CTAGAGAGTAATGACTCAATTAAACCACCCGTATCATATTGTTAACATCAGACCTTGGCCGAT
TGTTAGAGCCTTAGCAGCTTTTACGTTAACTACCGGGCTTGTTCAATGATTTCATCAGTTTAA
CTCGTCTCTTTTTATTTTAGGAACACTAAGAGCATGCATTGTATCGATCCAGTGGTGACGTGA
TATTAGCCGTGAGAGAACACTCCAGGGGCACCATTCTTCTAAGGTAGGGACAGGATTACGAT
GAGGGATAATTCTGTTTATCGCATCTGAAATTTTTTTCTTTGTATCGTTTTTTTGGGCTTTCTT
TCATAGCAGACTCTCTCCCAATGTAGAAGTAGGGGCAGTATGACCCCCAATCGGGATTGAAG
GATTTAACCCCTTCCAAATCCCTTTATTAAATACTAGGATTCTCTTGGCCAGAGGGGTGACTA
TCACATGAGCCCACCACGGGCTAATAGAAAATAATTTTGACCAGTGTAAACAGGGACTGATA
ATAACTGTTGCTCTCGGGGTATATTTCTCATTCCTTCAAGGAGTGGAGTATATCGAGGCATCA
TTTTCTATCGCTGACAGCGTTTACGGGTCCACCTTTTTTGTAGCGACAGGATTTCATGGACTT
CATGTAATTATCGGGTCATCATTCCTACTAGTATGCCTCACACGGCACTTACGATGCCACTTC
TCCCAAGAACATCACTTTGGATTCGAAGCTGCTGCTTGATACTGACATTTTGTGGATGTAGTG
TGGCTTTTCTTGTACATCTCTATCTACTGATGAGGTGAGTAACTTGTTTAGTATATAAGTATAT
CTGACTTCCAATCAGAAGGTCCATGTATGGAGTAAGTAATTATTAGAATTGTCCTGCTCTGAA
TTTCACTGATGATGCTGTCTAGAATCATACTATTAGCCAGTAGAATAATTAGAAAAAAGACAG
TAATAGAACGAGAAAAGAACTCACCCTTTGAGTGCGGATTTGACCCGCAAAATTCAACACGA
ATACCGTTCTCGCTGCGATTCTTCGTTATCACACTCATTTTCCTTATTTTCGATGTAGAAATC
ACACTTCTACTCCCAATCAATTCTCTAACGGGGCTAGAAATTATGAGGGTCACGATTCTTGTA
TTCTTTATTATTATTTTAGTAGCAGGAGTCTTATACGAATGAGAAGAGGGGGCGCTCAGATGA
ATTAAGTAGGAGCATAGTTTAACTAAAATACTTGATTTGCAATCAAGAGATAACAAGTGTTTG
CTCTCAGGAAAGGAATTGATTTATCATTCTTAGTTTCGACCTAAAATTAGGCTTTTAGCCCCT
TTCTTTAATAGAAGCTATTGAGCAATCCACTGTTACTGGAGAGAAAGGATCTATTTATCCCTA
TTAAAAGACAAAGAAGAGCCACGGAAAGCTTCTAACTTTACCTTGAGGTAGTTTAAATCTACC

TTTTGTCTGCTTTAATAGTATAAACAAATACGTCACATTTTCAATGTGTAGATAGAGTCACTCT
TTATAGCTGTCTAAGAGACAAATTTATAGAGTCAGAGATAAAGAAAAAAGGAAGTTCGGGGG
CCTCTCCTTCTCCTGTATACTCTCAC
>Eu36_scaffold-12
GTAGGACAATAGTGACTAAAGGAGGAGAGACGTAGTCGTACTCGAATAAGGGAACCATAGGT
TTCCTTAATGAGGAGAGACTAGGGGCTCTCAGTAAGGAAGTAAAGACTGCTTGTAACGTGGA
CCTTGTTCATAATATTACAGTAATCATCAACTAATATTTATATCTCTTTTAATCCTTTATTAGA
GTTTTATTTTGGTCGTCTACAAACTGTAAATTTCGTAAAGTTTGATCCTGTCTAGTAGTCTAAT
TTTTCCAGAGACAATATGCATATTCTTTCATCTGGAGTTATAAGTGCAGTTGTTAATATTAGA
GTTTATCACTATAGAACTAGTCAAAGAATGTTAGACAAGCCAACCTTGTGCCAGCAGCTGCG
GTCATACAAGGGTGTTGAGTTAGATTATTTAAGTTAAATTTCTTTCTTTTTTGGAGTAAAAATA
ACCTTTTTAAGGGTGAAATCTAAATATTTTATTTAAACTTCTTTATAATTTGATCTGTAGCATC
TATACCACTGGGATTAGATACCCCACTACTTTGCTTGCAATATACCTTGGGTAGTATTCTAGA
AACCTAAAGGATTTGGCGGTTTTTAAACCTACTAGGGGAACCTGTCCTTTAATCGATAGTCCA
CGCGCTGGCACACTTTATTTTGTCTTCAGTATATATACCGCCGTCTAGGGGACTGTACTAACA
TTTCCTCTTAATAAGGGAATCTTTATAAGTCAGGTCAAGGTGTAGCTTATGGTAAAGTAGAGA
TGGGTTACAATAACTCGTTACACGAAAGATGATCTTTAATTATTATCTGAAGGTGGACTTAGA
AGTAAATCTTTTCAATTTGAAGTAGGCTCTTAGGAATGTACACATCGCCCGTCGCTCTCTTGT
CTGAAGAGATAAGTCGTAACAAAGTAGTCATACTGGAAGGTATGTCTAGACAGACTAGAAGG
GAGCTTGAATAAGTTCCTCACTTACACTGAGGAGATCCCGTTAGGGCCTTCTAAAGATATGG
CATGCCTGTTTTTTGTATTCAAACAATTGTAAGAGGAGTAATAGACATAAAACATCGTATCGG
CGTTTAGTATCGTGAGAGAAATTATTTATATTGTTAATAAGTTGTTGTACGTACCTTTTGTATC
AGGGTTGGTCAATTTTACTTCTTTAAAGAGTATTCCCGAAATTTTGTGAATTACCCTTCTTGAT
TGTGTATGTTTCATCATGCTAGTTAATTCTAGGGTCGTTTCTATAAGTCATTCGGACAAATAT
ATCTGGTTGATCATCTGTCCTTTTAGAGGAGGTAATATTCTTTACTTAGTTGATAGGGGATGA
GCTCTATTAATAGTTAGATACTTTTATAATTTTTATACTCTTTAAAGCTTAGAAGTAGCTGAAG
TTTAGCAACTTGGGTATGAATAAAAAGATTTAAGAAGTATTAAAACTATGGATGCTTCGTGTT
TTACATTAATCTCAGACACAGATAATGATTAAATGAGTAAAATTAAAAGTCCTAAAGTATAAC
CATTTATACTAAGTCAAATTCTTTTAAAGCAAGAAAGGAACTCGGCAAAGAAATCTCCGCCTG
TTTAACAAAGACATGGCCTTATAGTTTTGTAAGGTCTAGCCTGCCCACTGATGATTTGAAGGG
CCGTGGTATACTGACCATGCGAAGGTAGCATAATCATTTGCCCCTTAATTAGGGGCTAGAAT
GAAGGGCTGGACGAGGGACTTCCTGTCTCTTTTGTTAACTTGAAATTAATCACTGGGTGAAAA
AGCTCAGGTTATCTTGAAGGACGATAAGACCCTATGGATCTTTATAAATTTACTTGAAGAGTT
TGGCTGTTAGAATCTTAGAGAAGTTTTATTGTGTTGGGGCGACACTGAGAGGAGAATAATCC
CTCAGTTTACTGAAACACATCTTTGTGGTTAAAGATCCTTGATTGAAATTAGATTAAGTTACC
CTAGGGATAACAGCGTTATATCTTTTGAGAGTTCATATCGACAAAGATGATTACGACCTCGAT
GTTGGTTCAGGGAACCTACAAGGTGCAGCAGTTTTGAGAGGAGGTCTGTTCGACCTTTAAAC
CCCTACGTGATCTGAGTTCAGACCGGCGTAAGCCAGGTTAGTTTCTACCCTCAAAATATTTC
ATTTTAGAGTAGTACGAAAGGACCCTCTATAATTTTACCATCTTGGCAGACAAATGCAATAGG
CTTAGGACCTATCTATAGAACACTTTCTAGCTGGTAAGCTTTGCTTATAATATTATTATCCGT
CTTATTACAGATTATTCTCGTGTTGGTATCTGTCGCTTTCTTGACTCTTCTAGAACGTAAAGT
TCTAGGCTATATTCAACTTCGTAAGGGCCCTAATAAAGTTGGATTTCTAGGTCTTCTTCAGCC

TTTTGCTGATGGTATTAAGCTCTTTACTAAGGAGATGTCTCACCCCAGCATATCAAATACTCT
ACCCTTCTATATTTCTCCCGTTCTCAGTCTCTCCCTTTCCTTAATCAGATGAACCCTCATTCC
TTTTTATGGGTATATATACACTTACTCTTATTCGGTGGTTCTCTTTTTGTGTGTCGTGAGATTA
AGCGTGTATACCGTAATAACAGCCGGCTGGTCATCCAATTCTAAGTATTCCCTTCTAGGAGG
CATGCGGGCCGGTGCCCAAACCATCTCTTATGAGGTTTCACTTATTCTCGTTTTACTATCCCC
TCTTTTAATGTGAGGGGTTTATAGGTATCAATCTATTGTTCATATCTCTTCTTACACAGGTTCT
CTTGTATTGTTAATACTCCCCCTTTCTTTGGCTTGGCTTGTAACAATTCTGGCTGAAACAAAC
CGTACTCCTTTTGATCTAGCTGAAGGGGAGTCTGAGCTAGTTTCTGGCTTTAACACAGAGTAC
AGAAGGGTAGGTTTTGCCCTCATCATGTTGGCTGAATACGCCAGTATTCTTCTTATATCTTTT
ATATTTATACTCCTCTTTTCCGGAGTTAACACACTAGTCTTCATTTTTGTGGTTTATGTGTTTC
TTTGGTCCCGGGGTTCTTACCCTCGTTACCGTTATGATCATCTTATAGCCCTTTCTTGGAAAA
GATTTCTTCCTTTGTCTATTAGATTTATACCCCTTTATCTTGGTATAAGTTACTTACTTTAATG
TGTTAATAATGAGACCCGTTTTTTACTTCTTTTATAAAGGTCAAAATACCTTTATGCAGTAAAA
AAAATACTACGATAAAGCAAATTTTTATTTGTCTCATCAGATTCCTGGTGGCTTAATTCACCC
TTTACTGTTTTCAAGACAGTCACCTCTATCGGTCAAAGAACCACCTAATCATATTATCTAGTT
GTTTAAAAATAATAGGGGTGATAATGTAATAACTAAAGTACACCACGGTCAAAATTTGGCCTG
TTAAGATAAAGGGGTCTTCTACGGGTCGTGCTCCGATTCATGTAAGAAGGAGAACAGTTGAG
ATCAAAGATCAAAATAAAGTCTGTACTAACGGGTAAAATCTAATCCCTCGAAATTTAGGGCTG
AAAGTAATAGGTACTCTGGCTAGGATTAAGATTGAAACTACCAGTGCGAGTACCCCCCCCAA
TTTATTCGGAATAGATCGTAAAATAGCGTAGGCGAAAAGGAAGTATCATTCTGGTTGAATATG
AACAGGAGTGACGAGAGGATTGGCGGGGATGAAGTTGTCTGGGTCTCCTATAATATTAGGTT
CCAAAAGGGTTAAAATTACTAGCGCTGTTAGAAACACTAAGAATCCTACAATATCTTTAATAG
AGAAATAAGGGTGAAATGGTACTTTATCAATATTTCTATTAATCCCCAAAGGATTTCCTGACC
CAGTTTGATGTAAAAATAGTAAGTGGACTATAGTTATTCCGGCAAGTAGGAATGGTAGAACGA
AATGAAAAGTAAAGAATCGTGTCAAAGTAGGGTTATCTACAGCAAATCCTCCTCATACTCATT
GAACAATGTCTACTCCTATGTAAGGAATTGCAGACACGAGGTTAGTAATAACTGTAGCTCCC
CAAAAAGATATTTGTCCCCAAGGTAGGACATAGCCTAAAAAAGCTGTTCCTATCAGGATAAAT
AATAGTGCGATCCCAGTGTTTCATGTCTCTATGTATCGGAATGATCCGTAGTAAAGTCCTCGC
CCGATGTGGAGATATACACAAATGAAAAAAAAAGATGCTCCATTAGCATGTAATGATCGAAG
GAGTCATCCATAATTAACATCCCGACAAATGTGGGCCACACTTGAGAATGCAAGTTCAACGT
CCGCTGTGTAATGTATTGCTAAAAATAGGCCCGTAACGATTTGGGTGATTAGACATAGCCCC
AACAGTGACCCCATGTTTCACCAAATAGAGATATTTGAGGGAACTGGTAGATCAACGAGAGC
AGAATTAGCAATTCTTAGAGCTGGGTGATTCTTTCGTAGAGATACCATTATAAGAGTGATCGT
AGTGGCTTTTTGTTAACGTTCAAAAACTCTACAATAAGGAGAAGGGCTAAAAATAGGTAGATA
GTCAGAGAAAGATAGAGAGGCCCCCTACTTCAGTGTAGCACCCTGTCTATATTCTGATAAGA
TCTTTTATCAGCAGGATATATTACTTCAATTTTTGACCAGAGAACTGCGGTCATAATAGCGGT
TACAACTATCATTACTAAGGTGGCTCTATCTATGATGAACCTTTCATTTGCTCTCAGTGATGC
AACATACAGGAACATTACTAGAATTCCCCCTAAGAAGATAAGAAACAAAATAAGAGAGATTCA
GGGGGAAAGAGGTGTTAATGTAACACATACAACGATTGTCTGGAGTAGGAGACAGAGAGTAA
ACGATAAAGGGTGGTTGATGAAAATTATTGCGGAACTAATCGTTAGTATAATTAATATAATAT
CAAAAGGTAATTTAATAAGAATTCTACCTTTGGAAGGTAGGCGTGTGATATTTCACCCTCTTG
AAGACTCAGGGGACTTCCCAACTCTGGTTTACAAGACCAGTATGATAATTCACCACTTAGTCT

TATTGAATTTCTACTTCTCGTTTGTTTATTGATGGCCCTGATTATCTTTCTTTCCTCCACTAAG
CATCTTTTGGTTACTCTCTTATGTTTGGAGTTTCTTATCTTATTATTATTTGTATTACTGTGCT
ATTCTAGTTATCTGACTTATCTAAATAGATTTACTTATTTGACTTTATCCGTATGTGAGAGAGC
ATTAGGTCTTTCACTTTTAGTAACTTTGGTCCGGTCTTCTGGTTCAGATCAGGTATCTATATT
AGATGGCTAGAAAATTTTTTACACCCTCTTGTATTTTATTCTATTCTTCATTTTTTATCTTGTG
TTGTTCTTTTAAGTTCAGCTGATCCCATTATCTCCTTGTTCTTAGAGGTTGAATCTTCTATCTA
ATAATTATATGTTGGTCTCCTTCCTACAAATATACTTACACAACTCTTATTGTTCTTCTCTTCT
TACTGCTAGCTTTAACTTTCACTTCTAATTCTCTTATTTCTTTTTATATCTTCTTTGAGTCCTC
TTTAATCCCCACCTTTCTTCTAATTATAGGTTGAGGCTACCAGCCTGAACGTCTCCCTGCATC
TCTTTATTTTCTTTTTTATACCCTTTTGGCTTCATTACCTCTTCTTTTCATCCTTTTAGGGTTA
GAGATAGAATACCAGACCTCCCTTGTGTTTCTTCTACCCTCTAGCTCAGATAAACTCTTTGTG
CTTTTTACAATTCTTGCCTTTCTAGTTAAGTTGCCCATGTACTTTACTCACATCTGATTGCCC
AAAGCTCATGTAGAAGCTCCCGTTACGGGCTCGATGGTCCTAGCGGCTATTCTACTAAAGCT
AGGGGGGTATGGCCTGTTTCTAGTGCAACCTCTTATAGGAGTAAGTTTACTCGGCTATCTTTG
TCTTCTAGCGGGATGAGGGGGTGTTTTAAGATGCTTGCTCTCTCTCCGTCAAACTGATGTTAA
ATCATTGATCGCTTACTCAAGGGTTGCTCATATGGCACTTATCATTCTAGGAATGTTATTTAT
GGGCATTTATGGAAACAGCGCCTCTCTTCTCTTAATAATCGCTCACGGCCTTTGTTCTTCCGG
TCTTTTTTATCTAAGCTATCTCACGTATGTTTGTTTCGGTTCTCGTTCTTTTCTAATAACTCGT
GGCATCCTTGTCTTTTCTCCTTTTGTCACCTTATGATGGTTCCTTCTCATTATCTTCAATATGG
GAGTCCCCCCCTCCCTAAATTTGTTCTCGGAACTCTATTTGTTTGTTGCCTCTAGGGGATTAC
ATCTGTTGTGTCTTGTTCTAGCAGGCTTAATTTCATTTCTGTCAGCCTGTTACTGTTTGTTTCT
TTACTCCTGAACAATTCATGGGGAGACTCTGGTAAAAACTGGCCTTCAGTACTCTCACGCTA
GTGAACTCTTAGTAAGAACTCTCCATTTTATCCCTCTTCTTCTATTAGTGACCCTTTGTTCTTG
TAGTCTAAAGGAGGATTCTAGATTGTGATTCTAGCGGTGAATATCTCCAAGGACTTTGACTTA
CTACATTTATAGGTTCTTTCTTTTCCTCATGGGGTGCATTTTTATGGGCTTATCTCTTTTATCC
TACCTGAGGGACTCAATTTATCTGGTTTCAGTGTCCTTTTTTTCTTTCGTTTATCAAGTACACT
TTGACTGAATTTCTCTTTCATTTCTTTTTCTCGTAAGGCTAATCTCTTCCCAAGTTCTAATGTA
CTCTACTTATTACATAGGAGGCGAGGCATTTTATGATCGTTTTAAGTATCTATTATTACTTTTT
GTCTCTTCTATGGCCTTTCTTATTATCTCTTCTGATGGTCTTAGTCTCCTATTGGGATGAGAC
GGGCTGGGAGTTACTTCTTATGCCTTAATTCTTTTTTACTCTAATTCTAAGTCTTCTTCAAGG
GGGATAATTACCGCCTTAAGTAACCGGGTTGGGGATATTCTTATTCTCTGATCTCTAGGTCTG
AACTATGCTCTAGGTTCTTGAGACTACAGGTTTATTTCCGTTGAATCTTCTTACGTAGTAATA
TTCCTCCTCTTATTAGCGTCTTTAACCAAAAGAGCTCAAATTCCTTTCTCAGCTTGATTACCC
GCGGCCATAGCAGCTCCTACCCCAGTTTCCTCACTTGTACACTCATCAACTCTTGTAACAGC
AGGTGTCTTTCTAATAATTCGTCTTTCTCCCTGTTTTTCTTACACGGGGAACCTGCTTTTAATT
TTACTTGGTTCTATTACCGCACTTCTCTCTGGTCTCGTGGCCATAGCGGAGTATGACCTGAAA
CGAGTCATTGCTCTTTCTACACTGAGTCAACTCGGCGTAATAATATTCTCCCTGGGGGTGGG
CTGTCCTCTCCTATGTTATTTTCATCTATTTACTCATGCTCTGTTTAAAGCTCTCTTATTTATG
TGTTCTGGAGTAATCATCCATTCTTCCGGGGGTACTCAAGATATTCGTCGTCTAGGAGGAGT
CTCTCTTTTCCTCCCGTTTTCTTGCTTAGCTCTTAGGGCTGCCTCTTTTAGTTTAATGGGGTT
TCCGTTTTTGGCCGGGTTCTACTCTAAGGATATGATTATTGAGAGTTCTACTAGAGAGTTATT
GATATTCCCATCTCTCGCTATTCTCGCTGCTGCTCTTCTGACCTGCGGCTATTCCTTCCGTCT

CATTTCTGTAGCTTTAACTTCTTCCGCCTGTAACTGAGTTTCCTTCATTAGTGGAGATGAAGG
AGATTACGTCGTTCCACTCAGGGTCTTATACAGGGGGGCTTTGTTTGGGGGAGTATTGTTTTA
TTGATCCTTTTTAGGTGTAGAGAGCCTTATGGTCTCTCCTATGGAAAAATTGACTTTGCTTGC
ATTTATTAG
>Ea3_scaffold-157
TTAGAGAATTGATTGGGAGTAGAAGTGTGATTTCTACATCGAAAATAAGGAAAATGAGTGTGA
CAACGAAGAATCGTATCGAGAATGGTATTCGTGTTGAATTTTGCGGGTCAAATCCGCACTCA
AAGGGTGAGTTCTTTTCTCGTTCCATTATTGTCTTTTTTCTAATTATTCTACTGGCTAATAGTA
TGATTCTAGACAGTATCATCAGTGAAATTCAGAGCAGGACAATTCTAATAATTACTTACTCCA
TACATGGACCTTCTGATTGGAAGTCAGATATACTTATATACTAAACAAGTTACTCACCTCATC
AGTAGATAGAGATGTACAAGAAAAGCCACACTACATCCACAAAATGCCAGTATCAAGCAGCA
GCTTCGAACCCAAAGTGATGTTCTTGGGAGAAGTGGCATCGTAAGTGCCGTGTGAGACACAC
TAGTAGGAATGATGACCCGATAATTACATGAAGTCCATGAAATCCTGTCGCTACGAAAAAGG
TGGACCCGTAAACGCTGTCAGCGATAGAAAATGATGCTTCAACATACTCTACTCCTTGAAGG
AACGAGAAATATACCCCGAGAGCAACAGTTATTATTAGCCCCTGTTTACACTGGTCAAAATTA
TTTTCTATTAGTCCGTGGTGGGCTCATGTGATAGTCACCCCTCTGGCCAAAAGAATTCTAGTA
TTTAATAAAGGGATTTGGAAGGGGTTAAATCCTTCAATCCCGATTGGGGGTCATACTGCTCCT
ACTTCTACATTGGGAGAGAGTCTGCTATGAAAGAAAGCCCAAAAAAACGATACAAAGAAAAA
AATTTCAGATGCGATAAACAGAATTATACCTCATCGTAGTCCTGTCCCCACCTTAGAAGAATG
GTGCCCCTGGAGTGTTCTCTCACGGCTAATATCACGTCACCACTGCATTGATACAATGCATG
CTCTTAGTGTTCCTAAAATAAAAAGAGACGAGTTAAACTGATGAAATCATTGAACAAGCCCGG
TAGTTAACGTAAAAGCTGCTAAGGCTCTAACAATTGGCCAAGGCCTGATGTTAACAATATGAT
ACGGATGGTTTAATTGAGTCATTACTCTCTGGCGTAGAGTGTAGATAAGGTGGCAAATACATA
CGCTTGAATAATTGCTACAGAAAATTCAAGCACCAGAAGTATTACTTGTCTTACTAATACGAT
TGCTATTGGCACTATTCCTGCTCCAATTGCTTGGTTACCCAGGAGGGTAAGAAGTAGGTGAC
CAGCAATTATATTGGCGGCCAGTCGTACTGAAAGGGTGACAGGCCGGATAAAATTACTTACT
AGCTCAATTACCACCATAAATGGCATCAGAACAGGGGGAGTTCCTAGCGGTACTAAATGTGC
TAATGCGTGCTTACTCTCTTTAATTCAGCCATATAGTAAGAATGATAGCCATAAAGGCAGTGC
TAGAGCAAGAGTGACAGCTAGATGACTGGTTGCAGTAAAAAGATAGGGGAATATCCCTATTA
AATTGTTGAAAAGAATAAATGAAAAAACTCTTACGAGTAAAAGTGGTGATCCTCTTTTATTAG
GTCCTAACAAAAGAGAAAACTCCTGGTCCAGTTTGGTCATAACAGTACCTCAAATTATTTGAA
TTCGTGTCGGTATAGCTCAGAACGCTCAGGGTAGCATAAAAAATGTTAACGTTATGGACAGT
CAGTTGGTTAAAAGGGAGGATGTAGGGTCAAATACCGAAAATAGATTACTTATCATGTTCAAT
CATTTACTCTTAATCGCGGAGTACTAACTTTATTTTTAAGGGTCACAGGTTGGGTAATGAAGT
AAATTATAGTTATCACCAGAGCTACAAATATAACTGCAGTAATTAATAGAGTCATTCACGGGA
GTGGAGCTATTTGTGGTATAAGAAATTACTCTGTGAGTACCTGAAGAATGACACTCTCCTATT
CTGATAAACTCAATTCCTATCATAAGAGGTAGTAACACCCTACTAAGGCTTAAAAAGCCTTCG
CTCTCTATCAGCCATCCTATGAAATTTGTCCTTGCAATCAGTTGAGGAACCTTCTAGTATTTA
CTGATTCTAGAACGATTGGTATAAACCTATGGCCCGCTCCACAGATTTCTGAGCACTGTCCG
TAGAAAATGCCTGGCATATTAATAAGTATCCCTGATTGGTTCAGTCGTCCAGGGATAGCATCT
ATCTTGATTCCAATGGAAGGAAGAGCCCACGAATGCAGAACATCGTCGGATGTAATTATAAG
GCGAATTGGTTGATTAAAAGGGAGGGGGACTCGGTTATCTACATCTAAAAGGCGGAATAATC

CTTGTTCCAGGTCTTGAGTAGGCACTATATAGGAGTCGAATTGTAAGTCATTAAAATCTGAGT
ATTCATATCCTCAGTATCATTGATGTCCTGTCACTTTAATGGTTAGAGCCGGGTTTTGTACTT
CATCAATTAAGTAAAGGAGTCGGATAGAAGGCAAAGCAATAAAAATTAGGATTACTGCAGGT
AACACAGTCCAAATAGTTTCAATCATTTGCCCATCCAGCAAATAGCGATGTACGTAAGTATTA
AAAAACAGAGACGCTATAAAAAACCCTACTAAAGACGTAATTAACACCACGATTAGCAATGC
ATGATCATGAAAAAATACCAATTGCTCTATTAGAGGGGATACCCCGTTTTGTAGTCCTAACTG
AAACCATTGAGACATCTTCTAAAGGGGAAGTTTCCCTTGGATGGAGCTTAAGTCCATTGCATT
TAATTTCTGCCACATTAGAAAGAAGAGATAAGAGTTAGTTCCTCGTATCTATGGTCGGCGGGT
GGATGAGGGTGATGTCATTCAATGTTAGACCTTAAATTAAGAGAAAAGAGGTTTGCTCGTTTT
AAGGTAAAGGCCTCTCACAGACAAAAGATAAACCCGAGGGTCCCCAAAAAAGAAATAATTCT
TCCTACTGAAGAAACCATGTTTCAAGAAGCATAACTGTCCGGGTAATCAGCGTATCGTCGCG
GTATTCCTGCTAGCCCGAGAAAATGTTGAGGAAAGAAAGTAATGTTTACCCCCAAAAACATG
ATTATGAAATGAATTTTTAAATAAGTTGTGTTGAGAGATACTCCTGTTAGAAGCGGGAACCAA
TGGGTAAATCCCCCTAAAATGGCAAATACAGCTCCCATTGAAAGGACATAGTGAAAATGCGC
TACAACGTAGTATGTATCGTGTAAGATAATATCAACTCTCGAGTTAGCTAACACCACTCCCGT
CAGTCCTCCTACAGTAAATAAGAACACGAATCCTAAAGCTCATATAAGAGAAGGTCTCATTGT
AAACCGGGTCCCGTGTAATGTCCCAATCCATCTAAAGATCTTGATCCCTGTAGGGATTGCAA
TAATTATAGTCGCAGCTGTGAAGTAGGCTCGTGTGTCGACATCTATACCCACCGTAAATATAT
GATGTGCCCAGACTACAAATCCTAATACTCCGATTGCTAGCATAGCATAAATTATACCCAAAG
TACCAAATGCTTCCTTTTTTCCTCTCTCTTGTCTGATAATGTGAGACACTATGCCAAATCCGG
GGAGAATTAAGATGTACACCTCAGGATGCCCAAAAAATCAAAAGAGATGTTGGTAAAGAATA
GGATCTCCTCCTCCCGCAGGATCGAAGAACGACGTGTTTAGATTACGATCTGTTAATAGTAT
AGTGATGGCTCCTGCTAAGACAGGCAGAGACAGCAGGAGAAGAACTGCTGTAATTCCAACTG
ATCACACAAAAAGTGGTATCCGGTCCAGGGACATTGACTGGGGCCGCATATTTAAAATAGTA
GTAATGAAATTAACTGCTCCTAGGATCGAAGAAATCCCTGCAAGATGGAGAGAAAAAATAGA
GAGATCCACAGATGGTCCTGCATGGGCGATTCCTGACGAGAGAGGGGGATACACCGTCCAT
CCGGTCCCTGCTCCCCTTTCTACTAGAGACCCTGCTAATAAGAGAGTCAAAGATGGTGGTAG
TATTCAAAATCTTAAGTTGTTAAGGCGTGGGAAGGCTATATCTGGTGCCCCTAGCATGATTGG
TACTAGTCAATTACCAAACCCCCCAATTAGGATGGGCATTACTATAAAGAAAATCATGACAAA
AGCATGGGCGGTCACAATAACGTTGTAAAGTTGTTCATCCCCTAACAAGGATCCTGGCTGAC
CTAACTCAGCTCGAATCAACATCCTAAGAGAAGTGCCTACCATACCTGATCAAGCCCCAAAT
AAAAAATAGAGTGTACCAATGTCTTTGTGATTAGTAGAATAGAGTCATCGTTGCAAAAGTAGA
GTGGCTGATTTAGGTATAAGGCTGTAAACCTTAATAAGGAGTTGTACTTCCTCTACTAAGTCT
TATAGTTTAAAAGAATAAAAGACTGCAAATCTTTAGGTGCTCTTAGCTAAGACTTTATACCTT
GGTGTATTGTGCACTTAAGGTTTTGATCCTTGGGGTAGAGTTCTTATCTCTAGGTATAAAGAT
AGGGTGCCTGATTAAAAGGGTTACGTTGATACTGTAAACATGCATAAATGCCCCTGTCTAGA
ACCTAAGATTTTCTTATTATCAGCTTTGAAGGCTACTAGTTTGTGTTAACTTAAGGCTCTAAA
GTATAACTATAGGTGTAACCCAGTGAAATCTTACTACTCATCACCCCCAAAAATTACTACGAA
AGTTGGATACAGGGGATAAAATCGGGATTATTATAATTTTAAAGTAAAAAAATAGAGATAAGC
ACGTGGATGAGACAAGTCCGGCTAAAACCACTAGTGATGGTATTGAATTTTCTAGAGTTATTC
ATTTAATTATGAACCCTAGAAGAGGGGGAAGTCCCCCTAAAGAAAGGAGACAAGAACTAAGG
GCTCTTTTTATTCCTATTGACATGGGGATGTTCATTTGCCTGACGAAAGATACACCCAGTCTT

TGTAGAATCAATACTAAAGTTAGAGTAATTAATGTGTAAACTGAAAAATAAGATACTAGTAAT
GATATACTTCTTATATTTATAAATACTCAAGCCAAGTGTGCAATAGAGGAATAGGTGATAAGT
AGACGCAAGTTTGATTGTAAGATACCCCCCAAGGCTCCAATGAGAGCCGAAAACACCACAAT
GATTGTCCTATTGAATGTGAGCATTAAGAGACCTAGTAAGGGCCCCAATTTTTGAAAGGTCAG
TAAAAAAAGAACGGAAACTCACGTTATACTGGGCGTTACTGCCGGTACCCATAGATGGAGAG
GAGCTACTCCAATTTTTAGAAGTAACCCGCAGATTACCCCAATATAAAGTAGTGGGTTAATGA
CTCCTGATAGAAATAGAAGGGAACCTAATGATTGAACTAGAAAATATTTCAAACTTACTTCCT
TATCCTTCCATACCAAAATAGGGATAAACCTCAGGGCATTTAACTCTAGGGCTAGCCATAAC
CCTAGCCAAGACTCAGTAGAGAATATTATGATGTAAGACAGGATTAAAATTAATCACTTTATT
ATAAAGAAGACAACTACTTTTCCTTAAAGAGGAGATGACTCTATCGGCAGGGTATGAACCTA
ATAGCTTTTTATTTAGCTTACCTCTTTGAAAGGATGAATTATAAATACGAGTTAAATTTGTGGT
CAGTCACGACCAGTTAAAATCAATTCTATGGATGTTAGCCCCATATAAAGATACAAAATACCA
AATTCTGTCAAGAACAAGAAAACTCGGATACATCAATTAAGATATGTTGAGTAAGAGACAGCT
TTTTTATGCCCCTGCTATAAATGCCAAGTAGTCTACAGCATTCTATTCAAGTCTTTGTTCTCA
CTGATCACCTTAGTTTTGCTGGGAGGCTACTCGCATAAGCTTATCTGAGATAACGAGTAGGTT
GCGTAAACAACCTAATTAGGAACATATATGGCTATCTGTAGAGAGGGGAGGTATGGGGGACT
AGTATT
>Ea3_scaffold-12
AATACTAGTCCCCCATACCTCCCCTCTCTACAGATAGCCATATATGTTCCTAGAAAAGAATTC
ACCCTAACATCTTCAGTGTTATGCTCTAGTTAAGCTATAGGAACTAGATAAACCAAATCCCAG
TAAAAGACACTAAAAGAAGGAGTTTAAACCCTACTGACGTGAAAGTTTCACTTAGAGAACTGA
GAAAGAGAATTCCTGTCTTCTGAACACCAGGACCTAAGTGTTCTACTCAGCCGTGGTCTCCT
TGGTTATAGACGATGTCTCCTCTTTTAAGTAATGGGGCCCTAAGAACAGATGTTAAAAAAGGA
AGAAACATTATGAAGGATAAAAAATGAAGTGTATGCCCTTTACTTATTATCAGGCTAAAGCCT
ACTAAAATACCTACTCTAATAAATGCAAGCAAAGTCAGTTTTTCCATAGGAGAGACCATAAGG
CTCTCTGCACCTAAGAAGGATCAATAAAACAATACTCCCCCAAACAAAGCCCCTCTGTATAA
AACCCTGAGTGAAACGACGTAATCTCCTTCATCTCCACTAATAAAGGAAACTCAGTTACAGG
CGGAAGAAGTTAAAGCTACAGAAATGAGACGAAAGGAATAGCCGCAGGTCAGAAGAGCGGC
AGCGAGAATAGCGAGAGATGGGAATATCAATAACTCTCTAGTAGAACTCTCGATAATCATAT
CCTTAGAGTAGAACCCGGCCAAAAACGGAAACCCCATTAAACTAAAAGAGGCAACTCTAAGA
GCTAGGCAAGAAAACGGGAGGAAAAGAGAAACTCCTCCTAGACGACGAATATCTTGGGTACC
CCCGGAAGAATGGATGATTACTCCAGAACACATGAATAAGAGAGCTTTAAACAGAGCATGAG
TAAATAGATGAAAATAACACAGGAGAGGACAGCCCACCCCCAGGGAGAATATTATAACGCCA
AGTTGACTCAGTGTAGAAAGAGCAATGACTCGTTTCAGGTCATACTCCGCTATGGCCACGAG
ACCAGAGAGAAGTGCGGTAATAGAGCCAAGTAAGATTAAAAGCAGGTTCCCCGTGTAAGAAA
AACAGGGAGAAAGACGAATTATTAAAAAGATACCTGCTGTTACAAGAGTTGATGAGTGTACA
AGTGAGGAAACGGGGGTAGGGGCTGCTATAGCCGCGGGTAACCAAGCTGAGAAAGGAATTT
GAGCTCTTTTGGTTAAAGACGCTAATAAGAGGAGAAATATTACTACGTAAGAAGATTCGACG
GAAATAAACCTGTAGTCTCAAGAACCTAAAGCATAGTTCAGACCTAGAGATCAGAGAATAAG
AATATCCCCAACCCGGTTACTTAAGGCGGTAATTATCCCCCTTGAAGAAGACTTAGAATTGG
AGTAAAAAAGAATTAAGGCATAAGAAGTAACTCCCAACCCGTCTCATCCTAATAAGAGACTA
AGACCATCAGAAGAGATAATAAGAAAGGCCATAGAAGAAACAAAAAGTAGTAATAGATACTT

AAAACGATCATAAAATGCCTCCCCTCCTATGTAATAAGTAGAGTATATTAAAACTTGGGAAGA
GATTAGCCTTACAAGAAAAAGAAATGAAAGAGAAATTCAATCAAATTGTACTTGATAAATGAA
AGAAAAAAAGGACACTGAAACTAGATAAATTGAGTCCCTCAGGTATGATAAAAGAGATAAGC
CTATAAAAATGCACCCCATGAGGAGAAGAAAGAACCTATAAATGTAGTAAGTCAAAGTCCTT
GGAGATATTCACCGCTAGAATCACAATCTAGAATCCTCCTTTAGACTACAAGAACAAAGAGTT
ACTAATAAAAGAAGGGGGATAAAATGAAGAGTTCTTACTAAGAGTTCACTAGCGTGGGAGTA
CTGGAGGCCAGTTTTTACTAGAGTCTCCCCATGAATTGTTCAGGAGTAAAGAAACAAACAGT
AACAGGCTGACAGGAATGAGATTAAGCCCGCTAGAACAAGACACAACAAATGTAATCCCCTA
GAAGCAACAAACAAATAGAGTTCCGAAAACAAATTTAGGGAAGGGGGGACTCCCATATTGAA
GATAATGAGAAAGAACCACCATAAGGTGACAAAAGGAGAAAAGACAAGGATGCCACGAGTTA
TTAGAAAAGAACGAGAACCGAAACAAACATACATGAGATAGCTTAGATAAAAAAGACCGGAA
GAACAAAGGCCGTGAGCGATTATTAAAAGAAGAGAGGCGCTGTTTCCATAAATGCCCATAAA
TAACATTCCTAGCACGATAAATGCCATGTGAGCAACCCTTGAGTAAGCGATCAATGATTTAAC
ATCAGTTTGACGGAGAGAGAGCAAGCATCTTAAAACACCTCCTCACCCAGCTAGGAGACAAA
GATAGCCGAGTAAACTTACTCCTATAAGGGGTTGCACTAGAAACAGGCCATACCCACCTAGC
TTTAGTAGAATAGCCGCTAGAACCATCGAGCCCGTAACGGGAGCTTCTACATGAGCTTTAGG
CAATCAGATATGAGTAAAGTATATGGGTAACTTAACTAGAAAGGCAAGAATTGTAAAAAGCAC
AAAGAGTTTATCTGAGCTAGAGGGTAGAAGAAAAACAAGGGAGGTCTGGTATTCTACCTCTA
ACCCCAAAAGGATGAAAAGGAGAGGTAATGAAGCCAAAAGGGTATAAAAAAGAAAATAAAGA
GATGCAGGTAGACGTTCGGGCTGGTAGCCTCAACCTATAATTAGAAGAAAGGTGGGGATTAA
AGAGGACTCAAAGAAGATATAAAAAGAAATAAGAGAATTAGAAGTAAAAGTTAAGGCTAGCA
GTAAAAAGAGAAGAATAATAAGAGTTATATAAGTATATTTGTAGGAAGGAGACCAACATATAA
TTATTAGATAGAAGATTCAACCTCTAAGAACAAGGAGATAATGGGATCAGCTGAACTTAAAAG
AACAACACAAGATAAAAAATGAAGAATAAAATAAGCCACAAGAGGGTGTAAAAAATTTCCTAG
CCATCTAATATAGACACCTGATCTGAACCAGAAGACCGGACCAAAGTTACTAAAAGTGAAAG
ACCTAATGCTCTCTCACATACGGATAAAGTCAAATAAGTAAATCTATTAAGATAAGTCAGATA
ACTAGAATAGCACAGCAACCCGAATAATAATAAGATAAGAAACTCCAGACATAAGAGAGTGA
CCAAAAGATGCTTAGTGGAGGAAAGAAAGATAATCAAGGCCATCAATAGACAAACGAGAAGT
AGGAATTCAATGAGACTAAGTGGTGAATTATCATACTGGTCTTGTAAACCAGAGTTGGGAAGT
CCCCTGAGTCTTCAAGAGGGTGAAATCTCACACGCCTACCTTCCAAAGGTAGAATTCTCATT
AAATTACCTTTTGACATTATATTAATCATACTAACGATTAGTTCCGTAATAATTTTCATCAACC
ATCCTTTATCGTTTACTCTATGTCTCCTACTACAGACAATCGTCATATGTGTCACATTGACAC
CTCTTTCCCCCTGAATCTCTCTCATTTTATTTCTTATCTTTTTAGGGGGAATTCTAGTAATGTT
CCTGTATGTTGCATCACTGAGAGCAAATGAAAGGTTCATCATAGATAGAACCACCTTAATAAT
AATAGCCGTAACCGCTATTATGATCGCAGTTCTCTGGTCAAAAATTGAAGTAATATATCCTGC
TGATAAAAGATCTTATCAGAATATAGACAGGGTACTGCATTGAACGAGGGGGCCTCTCTATC
TTTCTCTGACTATCTACCTATTTTTAGCCCTTCTCCTTATTGTAGAGTTTTTGAACATTAATAA
AAAGCCATTACGATCACTCTTATAATGGTATCTCTACGAAAGAATCACCCAGCTCTAAGAATT
GCTAATTCTGCTCTCGTTGATCTACCAGTTCCCTCAAATATCTCTGTTTGGTGAAACATGGGG
TCACTGTTGGGGCTGTGTCTAATCACCCAAATCGTTACGGGCCTATTTTTAGCGATACATTAC
ACAGCGGACGTTGAACTTGCATTCTCAAGTGTGGCCCACATTTGTCGGGATGTTAATTATGG
ATGACTCCTTCGATCATTGCATGCTAATGGAGCATCTTTTTTTTTCATTTGTGTGTATCTTCAC

ATCGGGCGAGGACTTTACTACGGATCGTTCCGATACATAGAAACATGAAACACGGGAATCGC
GCTATTATTTATCCTGATAGGGACAGCTTTTTTAGGTTATGTCCTACCTTGGGGACAAATATC
TTTTTGGGGAGCTACAGTTATTACTAACCTCGTGTCTGCTATTCCTTACGTAGGAGTAGACAT
TGTTCAATGAGTATGAGGAGGATTTGCTGTAGATAACCCTACTTTGACACGATTCTTTACTTT
TCATTTCGTACTACCGTTCCTACTTGCCGGAATAACTATAATCCACTTACTATTTTTACATCA
AACTGGGTCAGGAAATCCTTTAGGTATTAATAGTAATATTGACAAAGTACCATTTCACCCTTA
TTTCTCTATTAAAGATGTTGTGGGATTCTTAGTATTCCTAACGGCGCTAGTAATTTTAACCCT
TTTGGAACCTAATATTATAGGAGACCCAGACAACTTCATCCCCGCCAATCCTCTTGTCACTCC
TGTTCATATTCAACCAGAATGATACTTCCTTTTCGCCTACGCTATTTTACGATCCATCCCGAA
TAAATTAGGGGGGGTACTCGCATTGGTAGTTTCAATCTTAATTCTAGCCAGAGTACCTGTTAC
TTTTAGACCTAAATTTCGGGGGATCAGATTTTACCCGTTAGTACAAACTTTATTCTGGTCTTT
GATCTCAACTGTTCTCCTTCTTACATGAATCGGGGCACGACCCGTAGAAGACCCTTTTATCTT
AACAGGCCAAATTTTGACCGTAGTGTACTTTAGTTATTACATTATCACCCCTATTGTTTTTAA
ACAACTAGATAATCTGATTAGGTGGTTCTTTGACCGATAGAGGTGACTGTCTTGAAAACAGTA
AAGGGTGAATTAAGACACCAGGAATCTGATGAGACAAATAAAAATTTGCTTTATCGTAGTATT
TTTTTTACTGCATAAGGATATTTTGACCCTAATAAAATAGGTGAAAACCGGGTCTCGTTATTA
ACACATTAAAGTAAATAACTTATACCGAGATAAAAGGGTATAAATCTAATAGACAAAGGAAGA
AATCTTTTCCAAGAAAGGGCTATAAGATGATCATAACGGTAACGAGGGTAAGAACCCCGAGA
CCAGAGGAACACGTAAACCACAAAAATAAAGACTAGTGTATTAACTCCGGAAAAGAGGAGTA
TAAATATAAAAGATATAAGAAGAATACTGGCGTATTCTGCCAACATGATGAGGGCAAAACCTA
CCCTTCTGTACTCTGTGTTGAAGCCAGAAACTAGCTCAGACTCCCCTTCAGCAAGATCAAAA
GGAGTACGGTTTGTTTCAGCCAGAATTGTTACAAGCCAAGCCAAAGAAAGAGGGAGTATTAA
CAGCACAAGAGAACCGGTATACGAAGAGATATGAACAATAGATTGATAGCTATAAACCCCTC
ACATTAAAAGAGGGGATAGTAGAACGAGAATAAGTGAAACCTCATAAGAGATGGTTTGGGCA
CCGGCCCGCATGCCTCCTAGAAGGGAATACTTAGAGTTGGATGACCAGCCGGCTGTTATTAC
GGTATACACACTTAATCTCACGACACACAAAAAAAGAACCACCGAATAAGAGTAAGTATATAT
GTAGCCGTAAAAAGGAATGAGGGTTCACCCGATTAAAGAAAGGGAGAGACTGAGAACGGGAG
AAATATAGAAGGGTAGAGTATTCGACATGCTGGGGTGAGACATCTCCTTAGTAAAGAGCTTA
ATGCCATCAGCAAAAGGCTGAAGAAGACCTAAAAATCCAACTTTATTGGGGCCCTTACGAAG
TTGGATATACCCTAGAACTTTACGTTCTAGAAGAGTCAAAAAAGCGACAGATACCAGCACAA
GAATAATCTGTAATAAAACGGATAATAATATTATAAGCTTAGCTTACCAGCTAGAAAGTGTTC
TATAGATAGGTCCTAAGCCTATTGCATTTATCTGCCAAGATGGTAAAATCCTAGGGGGTCCTT
TCGTACTACCCTAGAATGAAATATTCTGAGGGTAGAAACTAACCTGGCTTACGCCGGTCTGA
ACTCAGATCACGTAGGGATTTAAAGGTCGAACAGACCTCCTCTCAAAACTGCTGCACCTTGT
AGGTTCCCTGAACCAACATCGAGGTCGTAATCATCTTTGTCGATATGAACTCTCAAAAGATAT
AACGCTGTTATCCCTAGGGTAACTTAGTCTAGTTTCAATCAAGGATCTTTAACCACAAAGATG
TGTTTCAGTAAACTGAGGGATTATTCTCCTCTCAGTGTCGCCCCAACACAATAAAACTTCTCT
AAGATTCTAACAGCCAAACTCTTCAAGTAAATTTATAAAGATCCATAGGGTCTTATCGTCCTT
CAAAATAACCTGAGCTTTTTCACCCAGTGATTAATTTCAAGTTAACAAAAGAGACAGGAAGTC
CCTCGTCCAACCCTTCATTCTAGCCCCTAATTAGGGGGCAAATGATTATGCTACCTTCGCAT
GGTCAGTATACCACGGCCCTTCAAATCATCAGTGGGCAGGCTAGACCTTACAAAACTATAAG
GCCATGTCTTTGTTAAACAGGCGGAGATTTCTTTGCCGAGTTCCTTTCTTGCTTTAAAAGGAT

TTGACTTAGTATAAGTAATTATACTTTAAAACTTTTAATTTTACTCATTTAATCATTATCTGTG
TTTGAGATTACTGTAAAACACGAAGCATCTATAGTTTTAATACTGCTTAAATCTTTTTATCTAT
ACCCAAGTTGCTAAACTTCAGCTACTTCTAAGCTTTAAAGAGTATAAAAATTATAAAAGTATC
TAACTATTGATAGAGCTCATCCCCTATCAATTAAGTAAAGAATATTACCTCCTCTAAAAGGAT
AGATGGTCAACCAGATATATTTGTCCGAATGACTTATAGAAACGACCCTAGAATTAACTAGCA
TGATGAAACATGCACAATCAAAAAGGGTAATTCACAAAATTTCGGGAATACTCTTTAAGGAAG
TAAAATTGACCAACCCTGATACAAAAGGTACGAACAACAACTTATTAACAATACAAATAATTT
CTCTCACGATACTAGCCGCCAATACGATGTTTTATGTCTATTACTCCTCTTACAGTTGTTTGC
GTACAAAAAAAAAGGCATGCCGTATTTTTAGAAGACCCTAGTGGGATCTCCTCAGTGTAAATG
AGGAACTTATTCAAGCTCCCTTCTAGTCTGTCTAGACATACCTTCCAGTACGACTACTTTGTT
ACGACTTATCTCTTCAGACAAGAGAGCGACGGGCGATGTGTACATTCCTAAGAGCCTACTTC
AAATTGAAAAGATTTACTTCTAAGTCCACCTTCAGATAATGATTAAAGACCATCTTTCGTGTA
ACGAGTTATTGTAACCCATCTCTACTTTACCATAAGCTACACCTTGACCTGACTTATAAAGAT
TCCCTTATTAAGAGGAAATGTTAGTACAGTCCCCTAGACGGCGGTATATATACTGAAAACAAG
ATAAAGTATGCCAACGCGTGGACTATCGATTAAAGGACAGGTTCCCCTAGTAGGTTTAAAAA
CCGCCAAATCCTTTAGGTTTCTAGAATACTACCCAAGGTATATTGCAAGCAAAGTAGTGGGG
TATCTAATCCCAGTGATATAGATGCTGCAGATCAAATTATAAAGAAGTTTAAATAAAATATTT
AGATTTCACCCTTAAAAAGGTTATTTTTACTCCAAAAAAGAAAGAAATTTAACTTAAATAGTCT
AACTCAACACCCTTGTATGACCGCAGCTGCTGGCACAAGGTTGGCTTGTCTAACATTCCTTG
ACTAGTTCTATAGTTATAAACTCTAATATTAACAACTGCACTTATAAATCCAGATGAGAAAAT
CTGCATATTGTCTCTAGAAAAATTAGACTACTAGACAGGATCAAACTTTACGAAATTTACAGT
TTGTAGACGACCAAAGTAAAACTCGAATAAAGAATTAAGAGAGATATAAATATAAGAAGATGA
ATACTGTAATATTACGGTCAAGGTCCATGTTACAAGCAGTATTTACTTCCTTACTGAGAGCCC
CTAGTCTCTCCTCATTAAGGAAACCTATGGTTCCCTTATTCGAGTACGACTACGTCTCTCCTC
CTTTAGTCACTATTGTCCTAC
>Ea4_scaffold-403293
TGAGAGTATACAGGAGAAGGAGAGGCCCCCGAACTTCCTTTCTTTTTTTTATATCTGACTCCA
TAAATTTGTCTCTTAGATAGCTATAAAGAGTGACTCTATCTGCACATTGAAAATGTGACGTAT
TTGTTTATACTATTAAAGCAGACAAAAGGTAGATTTAAACTACCTCAAGGTAAAGTTAGAAGC
TTTCCGTGGCTCTTCTTTGTCTTTTAATAGGGATAAATAGATCCTTTCTCTCCAGTAACAGTG
GATTGCTCAATAGCTTCTACTAAAGAAAGGGGCTAAAAGCCTAATTTTAGGTCGAAACTAAGA
ATGATAAATCAATTCCTTTCCTGAGAGCAAACATTTGTTATCTCTTGATTGCAAATCAAGTAT
TTTAGTTAAACTATGCTCCTACTTAATTCATCTGAGCGCTCCCTCTTCTCATTCGTATAAGAC
TCCTGTTACTAAAATAATAATAAAGAATACAAGAATTGTGACTCTCATAACTTCTAGCCTCGT
TAGAGAATTGATTGGGAGTAGAAGTGTGATTTCTACATCGAAAATAAGGAAAATGAGTGTGAC
AACGAAGAATCGTATCGAGAATGGTATTCGTGTTGAATTTTGCGGGTCAAATCCGCACTCAA
AGGGTGAGTTCTTTTCTCGTTCCATTATTGTCTTTTTTCTAATTATTCTACTGGCTAATAGTAT
GATTCTAGACAGTATCATCAGTGAAATTCAGAGCAGGACAATTCTAATAATTACTTACTCCAT
ACATGGACCTTCTGATTGGAAGTCAGATATACTTATATACTAAACAAGTTACTCACCTCATCA
GTAGATAGAGATGTACAAGAAAAGCCACACTACATCCACAAAATGCCAGTATCAAGCAGCAG
CTTCGAACCCAAAGTGATGTTCTTGGGAGAAGTGGCATCGTAAGTGCCGTGTGAGACACACT
AGTAGGAATGATGACCCGATAATTACATGAAGTCCATGAAATCCTGTCGCTACGAAAAAGGT

GGACCCGTAAACGCTGTCAGCGATAGAAAATGATGCTTCAACATACTCTACTCCTTGAAGGA
ACGAGAAATATACCCCGAGAGCAACAGTTATTATTAGCCCCTGTTTACACTGGTCAAAATTAT
TTTCTATTAGTCCGTGGTGGGCTCATGTGATAGTCACCCCTCTGGCCAAAAGAATTCTAGTAT
TTAATAAAGGGATTTGGAAGGGGTTAAATCCTTCAATCCCGATTGGGGGTCATACTGCTCCTA
CTTCTACATTGGGAGAGAGTCTGCTATGAAAGAAAGCCCAAAAAAACGATACAAAGAAAAAA
ATTTCAGATGCGATAAACAGAATTATACCTCATCGTAGTCCTGTCCCCACCTTAGAAGAATGG
TGCCCCTGGAGTGTTCTCTCACGGCTAATATCACGTCACCACTGCATTGATACAATGCATGC
TCTTAGTGTTCCTAAAATAAAAAGAGACGAGTTAAACTGATGAAACCATTGAACAAGCCCGGT
AGTTAACGTAAAAGCTGCTAAGGCTCTAACAATTGGCCAAGGCCTGATGTTAACAATATGATA
TGGATGGTTTAATTGAGTCATTACTCTCTGGCGTAGAGTGTAGATAAGGTGGCAAATACATAC
GCTTGAATAATTGCTACAGAAAATTCAAGCACCAGAAGTATTACTTGTCTTACTAATACGATT
GCTATTGGCACTATTCCTGCTCCAATTGCTTGGTTACCCAGGAGGGTAAGAAGTAGGTGACC
AGCAATTATATTGGCGGCCAGTCGTACTGAAAGGGTGACAGGCCGGATAAAATTACTTACTA
GCTCAATTACCACCATAAATGGCATCAGAACAGGAGGAGTTCCTAGCGGTACTAAATGTGCT
AATGCGTGCTTACTCTCTTTAATTCAGCCATATAGTAAGAATGATAGCCATAAAGGCAGTGCT
AGAGCAAGAGTGACAGCTAGATGACTGGTTGCAGTAAAAAGATAGGGGAATATCCCTATTAA
ATTGTTGAAAAGAATAAATGAAAAAACTCTTACGAGTAAAAGTGGTGATCCTCTTTTATTAGG
TCCTAACAAAAGAGAAAACTCCTGGTCCAGTTTGGTCATAACAGTACCTCAAATTATTTGAAT
TCGTGTCGGTATAGCTCAGAACGCTCAGGGTAGCATAAAAAATGTTAACGTTATGGACAGTC
AGTTGGTTAAAAGGGAGGATGTAGGGTCAAATACCGAAAATAGATTACTTATCATGTTCAATC
ATTTACTCTTAATCGCGGAGTACTAACTTTATTTTTAAGGGTCACAGGTTGGGTAATGAAGTA
AATTATAGTTATCACCAGAGCTACAAATATAACTGCAGTAATTAATAGAGTCATTCACGGGAG
TGGAGCTATTTGTGGTATAAGAAATTACTCTGTGAGTACCTGAAGAATGACACTCTCCTATTC
TGATAAACTCAATTCCTATCATAAGAGGTAGTAACACCCTACTAAGGCTTAAAAAGCCTTCGC
TCTCTATCAGCCATCCTATGAAATTTGTCCTTGCAATCAGTTGAGGAACCTTCTAGTATTTAC
TGATTCTAGAACGATTGGTATAAACCTATGGCCCGCTCCACAGATTTCTGAGCACTGTCCGT
AGAAAATGCCTGGCATATTAATAAGTATCCCTGATTGGTTCAGTCGTCCAGGGATAGCATCTA
TCTTGATTCCAATGGAAGGAAGAGCCCACGAATGCAGAACATCGTCGGATGTAATTATAAGG
CGAATTGGTTGATTAAAAGGGAGGGGGACTCGGTTATCTACATCTAAAAGGCGGAATAATCC
TTGTTCCAGGTCTTGAGTAGGCACTATATAGGAGTCGAATTGTAAGTCATTAAAATCTGAGTA
TTCATATCCTCAGTATCATTGATGTCCTGTCACTTTAATGGTTAGAGCCGGGTTTTGTACTTC
ATCAATTAAGTAAAGGAGTCGGATAGAAGGCAAAGCAATAAAAATTAGGATTACTGCAGGTA
ACACAGTCCAAATAGTTTCAATCATTTGCCCATCCAGCAAATAGCGATGTACGTAAGTATTAA
AAAACAGAGACGCCATAAAAACCCTACTAAAGACGTAATTAACACCACGATTAGCAATGCAT
GATCATGAAAAATACCAATTGCTCTATTAGAGGGGATACCCCGTTTTGTAGTCCTAACTGAAA
CCATTGAGACATCTTCTAAAGGGGAAGTTTCCCTTGGATGGAGCTTAAGTCCATTGCATTTAA
TTTCTGCCACATTAGAAAGAAGAGATAAGAGTTAGTTCCTCGTATCTATGGTCGGCGGGTGG
ATGAGGGTGATGTCATTCAATGTTAGACCTTAAATTAAGAGAAAAGAGGTTTGCTCGTTTTAA
GGTAAAGGCCTCTCACAGACAAAAGATAAACCCGAGGGTCCCCAAAAAAGAAATAATTCTTC
CTACTGAAGAAACCATGTTTCAAGAAGCATAACTGTCCGGGTAATCAGCGTATCGTCGCGGT
ATTCCTGCTAGCCCGAGAAAATGTTGAGGAAAGAAAGTAATGTTTACCCCCAAAAACATGATT
ATGAAATGAATTTTTAAATAAGTTGTGTTGAGAGATACTCCTGTTAGAAGCGGGAACCAATGG

GTAAATCCCCCTAAAATGGCAAATACAGCTCCCATTGAAAGGACATAGTGAAAATGCGCTAC
AACGTAGTATGTATCGTGTAAGATAATATCAACTCTCGAGTTAGCTAACACCACTCCCGTCAG
TCCTCCTACAGTAAATAAGAACACGAATCCTAAAGCTCATATAAGAGAAGGTCTCATTGTAAA
CCGGGTCCCGTGTAATGTCCCAATCCATCTAAAGATCTTGATCCCTGTAGGGATTGCAATAA
TTATAGTCGCAGCTGTGAAGTAGGCTCGTGTGTCGACATCTATACCCACCGTAAATATATGAT
GTGCCCAGACTACAAATCCTAATACTCCGATTGCTAGCATAGCATAAATTATACCCAAAGTAC
CAAATGCTTCCTTTTTTCCTCTCTCTTGTCTGATAATGTGAGACACTATGCCAAATCCGGGGA
GAATTAAGATGTACACCTCAGGATGCCCAAAAAATCAAAAGAGATGTTGGTAAAGAATAGGA
TCTCCTCCTCCCGCAGGATCGAAGAACGACGTGTTTAGATTACGATCTGTTAATAGTATAGTG
ATGGCTCCTGCTAAGACAGGCAGAGACAGCAGGAGAAGAACTGCTGTAATTCCAACTGATCA
CACAAAAAGTGGTATCCGGTCCAGGGACATTGACTGGGGCCGCATATTTAAAATAGTAGTAA
TGAAATTAACTGCTCCTAGGATCGAAGAAATCCCTGCAAGATGGAGAGAAAAAATAGAGAGA
TCCACAGATGGTCCTGCATGGGCGATTCCTGACGAGAGAGGGGGATACACCGTCCATCCGG
TCCCTGCTCCCCTTTCTACTAGAGACCCTGCTAATAAGAGAGTCAAAGATGGTGGTAGTATT
CAAAATCTTAAGTTGTTAAGGCGTGGGAAGGCTATATCTGGTGCCCCTAGCATGATTGGTAC
TAGTCAATTACCAAACCCCCCAATTAGGATGGGCATTACTATAAAGAAAATCATGACAAAAGC
ATGGGCGGTCACAATAACGTTGTAAAGTTGTTCATCCCCTAACAAGGATCCTGGCTGACCTA
ACTCAGCTCGAATCAACATCCTAAGAGAAGTGCCTACCATACCTGATCAAGCCCCAAATAAA
AAATAGAGTGTACCAATGTCTTTGTGATTAGTAGAATAGAGTCATCGTTGCAAAAGTAGAGTG
GCTGATTTAGGTATAAGGCTGTAAACCTTAATAAGGAGTTGTACTTCCTCTACTAAGTCTTAT
AGTTTAAAAGAATAAAAGACTGCAAATCTTTAGGTGCTCTTAGCTAAGACTTTATACCTTGGT
GTATTGTGCACTTAAGGTTTTGATCCTTGGGGTAGAGTTCTTATCTCTAGGTATAAAGATAGG
GTGCCTGATTAAAAGGGTTACGTTGATACTGTAAACATGCATAAATGCCCCTGTCTAGAACCT
AAGATTTTCTTATTATCAGCTTTGAAGGCTACTAGTTTGTGTTAACTTAAGGCTCTAAAGTATA
ACTATAGGTGTAACCCAGTGAAATCTTACTACTCATCACCCCCAAAAATTACTACGAAAGTTG
GATACAGGGGATAAAATCGGGATTATTATAATTTTAAAGTAAAAAAATAGAGATAAGCACGTG
GATGAGACAAGTCCGGCTAAAACCACTAGTGATGGTATTGAATTTTCTAGAGTCATTCATTTA
ATTATGAACCCTAGAAGAGGGGGAAGTCCCCCTAAAGAAAGGAGACAAGAACTAAGGGCTCT
TTTTATTCCTATTGACATGGGGATGTTCATTTGCCTGACGAAAGATACACCCAGTCTTTGTAG
AATCAATACTAAAGTTAGAGTAATTAATGTGTAAACTGAAAAATAAGATACTAGTAATGATAT
ACTTCTTATATTTATAAATACTCAAGCCAAGTGTGCAATAGAGGAATAGGTGATAAGTAGACG
CAAGTTTGATTGTAAGATACCCCCCAAGGCTCCAATGAGAGCCGAAAACACCACAATGATTG
TCCTATTGAATGTGAGCATTAAGAGACCTAGTAAGGGCCCCAATTTTTGAAAGGTCAGTAAAA
AAAGAACGGAAACTCACGTTATACTGGGCGTTACTGCCGGTACCCATAGATGGAGAGGAGCT
ACTCCAATTTTTAGAAGTAACCCGCAGATTACCCCAATATAAAGTAGTGGGTTAATGACTCCT
GATAGAAATAGAAGGGAACCTAATGATTGAACTAGAAAATATTTCAAACTTACTTCCTTATCC
TTCCATACCAAAATAGGGATAAACCTCAGGGCATTTAACTCTAGGGCTAGCCATAACCCTAG
CCAAGACTCAGTAGAGAATATTATGATGTAAGACAGGATTAAAATTAATCACTTTATTATAAA
GAAGACAACTACTTTTCCTTAAAGAGGAGATGACTCTATCGGCAGGGTATGAACCTAATAGC
TTTTTATTTAGCTTACCTCTTTGAAAGGATGAATTATAAATATGAGTTAAATTTGTGGTCAGTC
ACGACCAGTTAAAATCAATTCTATGGATGTTAGCCCCATATAAAGATACAAAATACCAAATTC
TGTCAAGAACAAGAAAACTCGGATACATCAATTAAGATATGTTGAGTAAGAGACAGCTTTTTT

ATGCCCCTGCTATAAATGCCAAGTAGTCTACAGCATTCTATTCAAGTCTTTGTTCTCACTGAT
CACCTTAGTTTTGCTGGGAGGCTACTCACATAAGCTTATCTGAGATAACGAGTAGGTTGCGT
AAACAACCTAATTAGGAACATATGTGGCTATCTGTAGAGAGGGGAGGTA
>Ea4_scaffold-4
AATGAGGAGAGACTAGGGGCTCTCAGTAAGGAAGTAAATACTGCTTGTAACATGGACCTTGA
CCGTAATATTACAGTATTCATCTTCTTATATTTATATCTCTCTTAATTCTTCATTCGAGTTTTA
CTTTGGTCGTCTACAAACTGTAAATTTCGTAAAGTTTGATCCTGTCTAGTAGTCTAATTTTTCT
AGAGACAATATGCAGATTTTCTCATCTGGATTTATAAGTGCAGTTGTTAATATTAGAGTTTAT
AACTATAGAACTAGTCAAGGAATGTTAGACAAGCCAACCTTGTGCCAGCAGCTGCGGTCATA
CAAGGGTGTTGAGTTAGACTATTTAAGTTAAATTTCTTTCTTTTTTGGAGTAAAAATAACCTTT
TTAAGGGTGAAATCTAAATATTTTATTTAAACTTCTTTATAATTTGATCTGCAGCATCTATATC
ACTGGGATTAGATACCCCACTACTTTGCTTGCAATATACCTTGGGTAGTATTCTAGAAACCTA
AAGGATTTGGCGGTTTTTAAACCTACTAGGGGAACCTGTCCTTTAATCGATAGTCCACGCGTT
GGCATACTTTATCTTGTTTTCAGTATATATACCGCCGTCTAGGGGACTGTACTAACATTTCCT
CTTAATAAGGGAATCTTTATAAGTCAGGTCAAGGTGTAGCTTATGGTAAAGTAGAGATGGGTT
ACAATAACTCGTTACACGAAAGATGGTCTTTAATCATTATCTGAAGGTGGACTTAGAAGTAAA
TCTTTTCAATTTGAAGTAGGCTCTTAGGAATGTACACATCGCCCGTCGCTCTCTTGTCTGAAG
AGATAAGTCGTAACAAAGTAGTCGTACTGGAAGGTATGTCTAGACAGACTAGAAGGGAGCTT
GAATAAGTTCCTCATTTACACTGAGGAGATCCCACTAGGGTCTTCTAAAAATACGGCATGCCT
TTTTTTTGTACGCAAACAACTGTAAGAGGAGTAATAGACATAAAACATCGTATTGGCGGCTAG
TATCGTGAGAGAAATTATTTGTATTGTTAATAAGTTGTTGTTCGTACCTTTTGTATCAGGGTTG
GTCAATTTTACTTCCTTAAAGAGTATTCCCGAAATTTTGTGAATTACCCTTTTTGATTGTGCAT
GTTTCATCATGCTAGTTAATTCTAGGGTCGTTTCTATAAGTCATTCGGACAAATATATCTGGT
TGACCATCTATCCTTTTAGAGGAGGTAATATTCTTTACTTAATTGATAGGGGATGAGCTCTAT
CAATAGTTAGATACTTTTATAATTTTTATACTCTTTAAAGCTTAGAAGTAGCTGAAGTTTAGCA
ACTTGGGTATAGATAAAAAGATTTAAGCAGTATTAAAACTATAGATGCTTCGTGTTTTACAGT
AATCTCAAACACAGATAATGATTAAATGAGTAAAATTAAAAGTTTTAAAGTATAATTACTTATA
CTAAGTCAAATCCTTTTAAAGCAAGAAAGGAACTCGGCAAAGAAATCTCCGCCTGTTTAACAA
AGACATGGCCTTATAGTTTTGTAAGGTCTAGCCTGCCCACTGATGATTTGAAGGGCCGTGGT
ATACTGACCATGCGAAGGTAGCATAATCATTTGCCCCCTAATTAGGGGCTAGAATGAAGGGT
TGGACGAGGGACTTCCTGTCTCTTTTGTTAACTTGAAATTAATCACTGGGTGAAAAAGCTCAG
GTTATTTTGAAGGACGATAAGACCCTATGGATCTTTATAAATTTACTTGAAGAGTTTGGCTGT
TAGAATCTTAGAGAAGTTTTATTGTGTTGGGGCGACACTGAGAGGAGAATAATCCCTCAGTTT
ACTGAAACACATCTTTGTGGTTAAAGATCCTTGATTGAAACTAGACTAAGTTACCCTAGGGAT
AACAGCGTTATATCTTTTGAGAGTTCATATCGACAAAGATGATTACGACCTCGATGTTGGTTC
AGGGAACCTACAAGGTGCAGCAGTTTTGAGAGGAGGTCTGTTCGACCTTTAAATCCCTACGT
GATCTGAGTTCAGACCGGCGTAAGCCAGGTTAGTTTCTACCCTCAGAATATTTCATTCTAGAG
TAGTACGAAAGGACCCCCTAGGATTTTACCATCTTGGCAGATAAATGCAATAGGCTTAGGAC
CTATCTATAGAACACTTTCTAGCTGGTAAGCTAAGCTTATAATATTATTATCCGTTTTATTACA
GATTATTCTTGTGCTGGTATCTGTCGCTTTTTTGACTCTTCTAGAACGTAAAGTTCTAGGGTA
TATCCAACTTCGTAAGGGCCCCAATAAAGTTGGATTTTTAGGTCTTCTTCAGCCTTTTGCTGA
TGGCATTAAGCTCTTTACTAAGGAGATGTCTCACCCCAGCATGTCGAATACTCTACCCTTCTA

TATTTCTCCCGTTCTCAGTCTCTCCCTTTCTTTAATCGGGTGAACCCTCATTCCTTTTTACGG
CTACATATATACTTACTCTTATTCGGTGGTTCTTTTTTTGTGTGTCGTGAGACTAAGTGTGTAT
ACCGTAATAACAGCCGGCTGGTCATCCAACTCTAAGTATTCCCTTCTAGGAGGCATGCGGGC
CGGTGCCCAAACCATCTCTTATGAGGTTTCACTTATTCTCGTTCTACTATCCCCTCTTTTAAT
GTGAGGGGTTTATAGCTATCAATCTATTGTTCATATCTCTTCGTATACCGGTTCTCTTGTGCT
GTTAATACTCCCTCTTTCTTTGGCTTGGCTTGTAACAATTCTGGCTGAAACAAACCGTACTCC
TTTTGATCTTGCTGAAGGGGAGTCTGAGCTAGTTTCTGGCTTCAACACAGAGTACAGAAGGG
TAGGTTTTGCCCTCATCATGTTGGCAGAATACGCCAGTATTCTTCTTATATCTTTTATATTTAT
ACTCCTCTTTTCCGGAGTTAATACACTAGTCTTTATTTTTGTGGTTTACGTGTTCCTCTGGTCT
CGGGGTTCTTACCCTCGTTACCGTTATGATCATCTTATAGCCCTTTCTTGGAAAAGATTTCTT
CCTTTGTCTATTAGATTTATACCCTTTTATCTCGGTATAAGTTATTTACTTTAATGTGTTAATA
ACGAGACCCGGTTTTCACCTATTTTATTAGGGTCAAAATATCCTTATGCAGTAAAAAAAATAC
TACGATAAAGCAAATTTTTATTTGTCTCATCAGATTCCTGGTGTCTTAATTCACCCTTTACTGT
TTTCAAGACAGTCACCTCTATCGGTCAAAGAACCACCTAATCAGATTATCTAGTTGTTTAAAA
ACAATAGGGGTGATAATGTAATAACTAAAGTACACTACGGTCAAAATTTGGCCTGTTAAGATA
AAAGGGTCTTCTACGGGTCGTGCCCCGATTCATGTAAGAAGGAGAACAGTTGAGATCAAAGA
CCAGAATAAAGTTTGTACTAACGGGTAAAATCTGATCCCCCGAAATTTAGGTCTAAAAGTAAC
AGGTACTCTGGCTAGAATTAAGATTGAAACTACCAATGCGAGTACCCCCCCTAATTTATTCGG
GATGGATCGTAAAATAGCGTAGGCGAAAAGGAAGTATCATTCTGGTTGAATATGAACAGGAG
TGACAAGAGGATTGGCGGGGATGAAGTTGTCTGGGTCTCCTATAATATTAGGTTCCAAAAGG
GTTAAAATTACTAGCGCCGTTAGGAATACTAAGAATCCCACAACATCTTTAATAGAGAAATAA
GGGTGAAATGGTACTTTGTCAATATTACTATTAATACCTAAAGGATTTCCTGACCCAGTTTGA
TGTAAAAATAGTAAGTGGATTATAGTTATTCCGGCAAGTAGGAACGGTAGTACGAAATGAAAA
GTAAAGAATCGTGTCAAAGTAGGGTTATCTACAGCAAATCCTCCTCATACTCATTGAACAATG
TCTACTCCTACGTAAGGAATAGCAGACACGAGGTTAGTAATAACTGTAGCTCCCCAAAAAGA
TATTTGTCCCCAAGGTAGGACATAACCTAAAAAAGCTGTCCCTATCAGGATAAATAATAGCG
CGATTCCCGTGTTTCATGTTTCTATGTATCGGAACGATCCGTAGTAAAGTCCTCGCCCGATGT
GAAGATACACACAAATGAAAAAAAAAGATGCTCCATTAGCATGCAATGATCGAAGGAGTCAT
CCATAATTAACATCCCGACAAATGTGGGCCACACTTGAGAATGCAAGTTCAACGTCCGCTGT
GTAATGTATCGCTAAAAATAGGCCCGTAACGATTTGGGTGATTAGACACAGCCCCAACAGTG
ACCCCATGTTTCACCAAACAGAGATATTTGAGGGAACTGGTAGATCAACGAGAGCAGAATTA
GCAATTCTTAGAGCTGGGTGATTCTTTCGTAGAGATACCATTATAAGAGTGATCGTAATGGCT
TTTTATTAATGTTCAAAAACTCTACAATAAGGAGAAGGGCTAAAAATAGGTAGATAGTCAGAG
AAAGATAGAGAGGCCCCCTCGTTCAATGCAGTACCCTGTCTATATTCTGATAAGATCTTTTAT
CAGCAGGATATATTACTTCAATTTTTGACCAGAGAACTGCGATCATAATAGCGGTTACGGCTA
TTATTATTAAGGTGGTTCTATCTATGATGAACCTTTCATTTGCTCTCAGTGATGCAACATACA
GGAACATTACTAGAATTCCCCCTAAAAAGATAAGAAATAAAATGAGAGAGATTCAGGGGGAA
AGAGGTGTCAATGTGACACATATGACGATTGTCTGTAGTAGGAGACATAGAGTAAACGATAA
AGGATGGTTGATGAAAATTATTACGGAACTAATCGTTAGTATGATTAATATAATGTCAAAAGG
TAATTTAATAAGAATTCTACCTTTGGAAGGTAGGCGTGTGAGATTTCACCCTCTTGAAGACTC
AGGGGACTTCCCAACTCTGGTTTACAAGACCAGTATGATAATTCACCACTTAGTCTCATTGAA
TTCCTACTTCTCGTTTGTCTATTGATGGCCTTGATTATCTTTCTTTCCTCCACTAAGCATCTTT

TGGTCACTCTCTTATGTCTGGAGTTTCTTATCTTATTATTATTCGGGTTGCTGTGCTATTCTAG
TTATCTGACTTATCTTAATAGATTTACTTATCTGACTTTATCCGTATGTGAGAGAGCATTAGGT
CTTTCACTTTTAGTAACTTTGGTCCGGTCTTCTGGTTCAGACCAGGTGTCTATATTAGATGGC
TAGGAAATTTTTTACACCCTCTTGTGGCTTATTTTATTCTTCATTTTTTATCTTGTGTTGTTCT
TTTAAGTTCAGCTGATCCCATTATCTCCTTGTTCTTAGAGGTTGAATCTTCTATCTAATAATTA
TATGTTGGTCTCCTTCCTACAAATATACTTATATAACTCTTATTATTCTTCTCTTTTTACTGCT
AGCCTTAACTTTTACTTCTAATTCTCTTATTTCTTTTTATATCTTCTTTGAGTCCTCTTTAATC
CCCACCTTTCTTCTAATTATAGGTTGAGGCTACCAGCCCGAACGTCTACCTGCATCTCTTTAT
TTTCTTTTTTATACCCTTTTGGCTTCATTACCTCTCCTTTTCATCCTTTTGGGGTTAGAGGTAG
AATACCAGACCTCCCTTGTTTTTCTTCTACCCTCTAGCTCAGATAAACTCTTTGTGCTTTTTA
CAATTCTTGCCTTTCTAGTTAAGTTACCCATATACTTTACTCATATCTGATTGCCTAAAGCTC
ATGTAGAAGCTCCCGTTACGGGCTCGATGGTTCTAGCGGCTATTCTACTAAAGCTAGGTGGG
TATGGCCTGTTTCTAGTGCAACCCCTTATAGGAGTAAGTTTACTCGGCTATCTTTGTCTCCTA
GCTGGGTGAGGAGGTGTTTTAAGATGCTTGCTCTCTCTCCGTCAAACTGATGTTAAATCATTG
ATCGCTTACTCAAGGGTTGCTCACATGGCATTTATCGTGCTAGGAATGTTATTTATGGGCATT
TATGGAAACAGCGCCTCTCTTCTTTTAATAATCGCTCACGGCCTTTGTTCTTCCGGTCTTTTT
TATCTAAGCTATCTCGTGTATGTTTGTTTCGGTTCTCGTTCTTTTCTAATAACTCGTGGCATC
CTTGTCTTTTCTCCTTTTGTCACCTTATGGTGGTTCTTTCTCATTATCTTCAATATGGGAGTCC
CCCCTTCCCTAAATTTGTTTTCGGAACTCTATTTGTTTGTTGCTTCTAGGGGATTACATTTGTT
GTGTCTTGTTCTAGCGGGCTTAATCTCATTCCTGTCAGCCTGTTACTGTTTGTTTCTTTACTC
CTGAACAATTCATGGGGAGACTCTAGTAAAAACTGGCCTCCAGTACTCCCACGCTAGTGAAC
TCTTAGTAAGAACTCTTCATTTTATCCCCCTTCTTTTATTAGTAACTCTTTGTTCTTGTAGTCT
AAAGGAGGATTCTAGATTGTGATTCTAGCGGTGAATATCTCCAAGGACTTTGACTTACTACAT
TTATAGGTTCTTTCTTCTCCTCATGGGGTGCATTTTTATAGGCTTATCTCTTTTATCATACCTG
AGGGACTCAATTTATCTAGTTTCAGTGTCCTTTTTTTCTTTCATTTATCAAGTACAATTTGATT
GAATTTCTCTTTCATTTCTTTTTCTTGTAAGGCTAATCTCTTCCCAAGTTTTAATATACTCTAC
TTATTACATAGGAGGGGAGGCATTTTATGATCGTTTTAAGTATCTATTACTACTTTTTGTTTCT
TCTATGGCCTTTCTTATTATCTCTTCTGATGGTCTTAGTCTCTTATTAGGATGAGACGGGTTG
GGAGTTACTTCTTATGCCTTAATTCTTTTTTACTCCAATTCTAAGTCTTCTTCAAGGGGGATA
ATTACCGCCTTAAGTAACCGGGTTGGGGATATTCTTATTCTCTGATCTCTAGGTCTGAACTAT
GCTTTAGGTTCTTGAGACTACAGGTTTATTTCCGTCGAATCTTCTTACGTAGTAATATTTCTC
CTCTTATTAGCGTCTTTAACCAAAAGAGCTCAAATTCCTTTCTCAGCTTGGTTACCCGCGGCT
ATAGCAGCCCCTACCCCCGTTTCCTCACTTGTACACTCATCAACTCTTGTAACAGCAGGTAT
CTTTTTAATAATTCGTCTTTCTCCCTGTTTTTCTTACACGGGGAACCTGCTTTTAATCTTACTT
GGCTCTATTACCGCACTTCTCTCTGGTCTCGTGGCCATAGCGGAGTATGACCTGAAACGAGT
CATTGCTCTTTCTACACTGAGTCAACTTGGCGTTATAATATTCTCCCTGGGGGTGGGCTGTCC
TCTCCTGTGTTATTTTCATCTATTTACTCATGCTCTGTTTAAAGCTCTCTTATTCATGTGTTCT
GGAGTAATCATCCATTCTTCCGGGGGTACCCAAGATATTCGTCGTCTAGGAGGAGTTTCTCT
TTTCCTCCCGTTTTCTTGCCTAGCTCTTAGAGTTGCCTCTTTTAGTTTAATGGGGTTTCCGTT
TTTGGCCGGGTTCTACTCTAAGGATATGATTATCGAGAGTTCTACTAGAGAGTTATTGATATT
CCCGTCTCTCGCTATTCTCGCTGCCGCTCTTCTGACCTGCGGCTATTCCTTTCGTCTCATTTC
TGTAGCTTTAACTTCTTCCGCCTGTAACTGAGTTTCCTTTATTAGTGGAGATGAAGGAGATTA

CGTCGTTTCACTCAGGGTTTTATACAGAGGGGCTTTGTTTGGGGGAGTATTGTTTTATTGATC
CTTCTTAGGTGCAGAGAGCCTTATGGTCTCTCCTATGGAAAAACTGACTTTGCTTGCATTTAT
TAGAGTAGGTATTTTAGTAGGCTTTAGCCTGATAATAAGTAAAGGGCATACACTTCATTTTTT
ATCCTTCATAATGTTTCTTCCTTTTTTAACATCTGTTCTTAGGGCCCCATTACTTAAAAGAGG
AGACATCGTCTATAACCAAGGAGACCACGGCTGAGTAGAACACTTAGGTCCTGGTGTTCAGA
AGACAGGAATTCTCTTTCTCAGTTCTCTAAGTGAAACTTTCACGTCAGTAGGGTTTAAACTCC
TTCTTTTAGTGTCTTTTACTGGGATTTGGTTTATCTAGTTCCTATAGCTTAACTAGAGCATAAC
ACTGAAGATGTTAGGGTGAATTCTTTTCTAGGAACATATATGGCTATCTGTAGAGAGGGGAG
GTA
>Ea3_scaffold-278847
TGGTTAGTACCAATCATGTTGGGAGCGCCAGATATGGCCTTCCCACGTCTTAACAACCTG
AGATTTTGGATACTACCACCATCTCTAACTCTTCTATTAGCAGGATCTCTGGTAGAAAGA
GGAGCAGGGACCGGATGAACGGTGTATCCCCCTCTTTCCTCAGGAATCGCACATGCAGGA
CCATCTGTAGATCTTTCTATTTTTTCTCTCCATCTTGCGGGGATTTCTTCGATCCTAGGG
GCAG
>Ea3_scaffold-458031
AACTCTCAAAAGATATAACGCTGTTATCCCTAGGGTAACTTGATCTAGTTTCAGTTAAGG
ATCTTTAACCACAGAGATGTGTTTCAATAGACTGAGGGATTATTCTCCTCTCAGTGTCGC
CCCAACACAATAAAACTTC
>Ea4_scaffold-236301
AACAACTTTACAACGTTATTGTGACCGCCCACGCTTTTGTCATGATTTTTTTTATGGTAA
TACCTATCTTAATTGGGGGGTTCGGTAACTGGTTAGTACCAATCATGTTGGGAGCGCCAG
ATATGGCCTTCCCACGTCTTAACAACCTGAGATTTTGGATACTACCACCATCTCTAACTC
TTCTATTAGCAGGATCTCTGGTAGAAAGAGGAGCAGGGACCGGATGAACGGTGTATCCCC
CTCTTTCCTCAGGAATCGCACATGCAGGACCATCTGTAGATCTTTCTATTTTTTCTCTCC
ATCTTGCGGGGATTTCTTCGATCCTAGGGGCAGTTAATTTCATCACTACTATTTTAAACA
TGCGGCCTCAATCAATGTCGCTGGACC
>Eu17_scaffold-250790
CCTGCAAGATGGAGAGAAAAAATAGAAAGATCTACAGATGGTCCTGCATGTGCGATTCCT
GAGGAAAGAGGGGGATACACCGTTCATCCGGTCCCTGCTCCTCTTTCTACCAGAGATCCT
GCTAATAGAAGAGTTAGAGATGGTGGTAGTATCCAAAATCTCAGGTTGTTAAGACGTGGG
AAGGCCATATCTGGCGCTCCCAACATGATTGGTACTAACCAGTTACCGAACCCCCCAATT
AAGATAGGTATTACCATAAAAAAAATCATGACAAAAGCGTGGGCGGTCACAATAACGTTG
TAAAGTTGTTCGTCCCCTAACAAGGATCCTGGCTG
>Eu36_scaffold-122170
GCCACTCTACTTTTGCAACGATGACTCTATTCCACTAATCACAAAGATATCGGCACACTT
TATTTTTTATTCGGGGCTTGATCAGGCATAGTAGGCACTTCTCTTAGAATGTTGATTCGA
GCTGAATTAGGTCAGCCAGGATCCTTGTTAGGGGACGAACAACTTTACAACGTTATTGTG
ACCGCCCACGCTTTTGTCATGATTTTTTTTATGGTAATACCTATCTTAATTGGGGGGTTC
GGTAACTGGTTAGTACCAATCATGTTGGGAGCGCCAGATATGGCCTTCCCACGTCTTAAC
AACCTGAGATTTTGGATACTACCACCATCTCTAACTCTTCTATTAGCAGGATCTCTGGTA
GAAAGAGGAGCAGGGACCGGATGAACGGTGTATCCCCCTCTTTCCTCAGGAATCGCACAT

GCAGGACCATCTGTAGATCTTTCTATTTTTTCTCTCCATCTTGCAGG
