# Supplementary material for: Complete mitochondrial genomes of three fairy shrimps from snowmelt pools in Japan
Source: BMC Zool. 2022 Feb 9;7:11. doi: 10.1186/s40850-022-00111-2 (PMC10127424; doi:10.1186/s40850-022-00111-2)
Supplement: Supplementary file 3 — Additional file 3. [file 40850_2022_111_MOESM3_ESM.pdf]

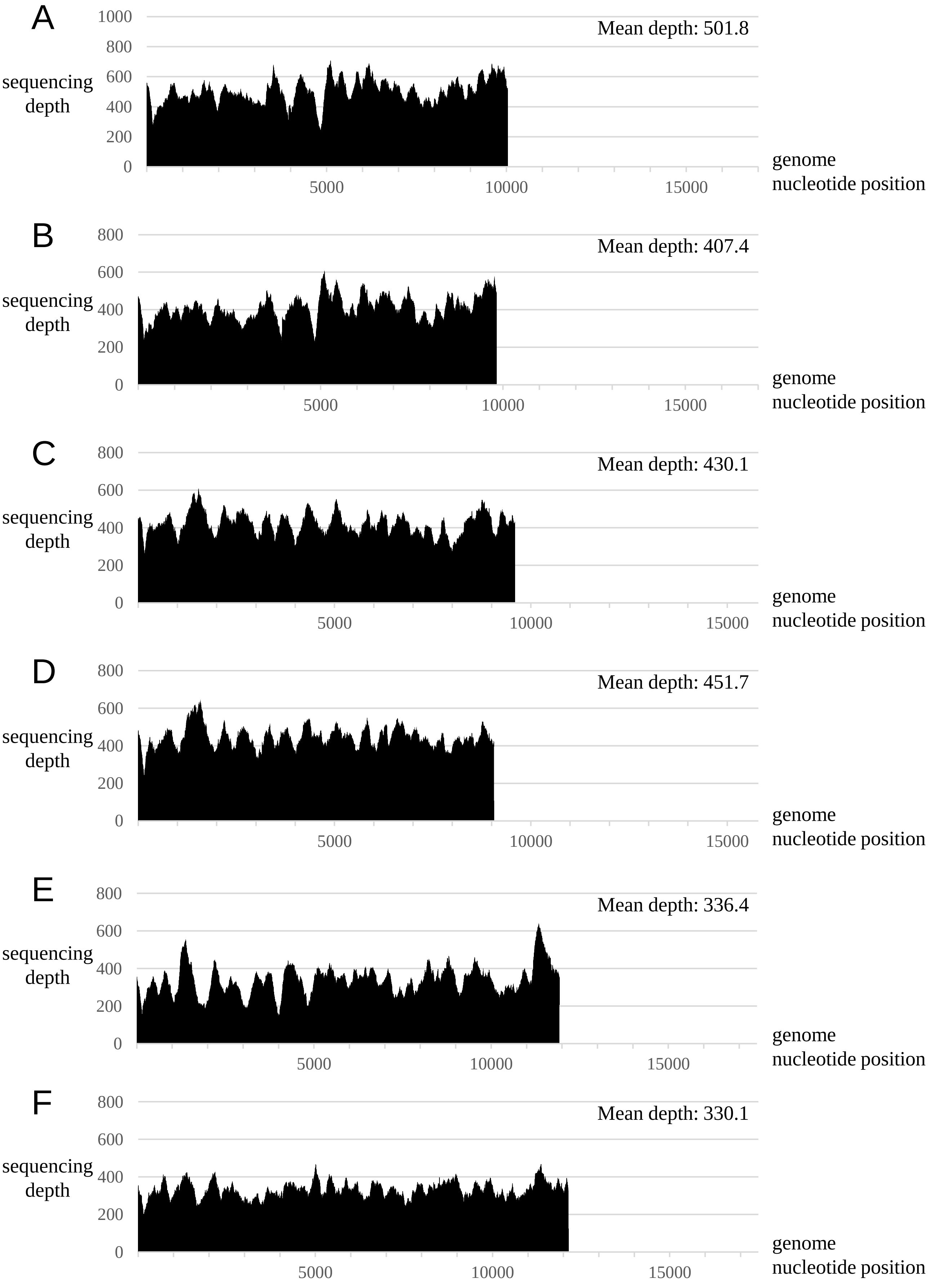

Supplementary Fig. S1 Sequencing depth of reads remapped to mitochondrial genomes of *E. hatanakai* (Eh1) (A), *E. hatanakai* (Eh6) (B), *E. uchidai* (Eu17) (C), *E. uchidai* (Eu36) (D), *E. asanumai* (Ea3) (E), and *E. asanumai* (Ea4) (F). Vertical axis: sequencing depth, horizontal axis: genome nucleotide position.
