# Supplementary material for: Complete mitochondrial genomes of three fairy shrimps from snowmelt pools in Japan
Source: BMC Zool. 2022 Feb 9;7:11. doi: 10.1186/s40850-022-00111-2 (PMC10127424; doi:10.1186/s40850-022-00111-2)
Supplement: Supplementary file 8 — Additional file 8. [file 40850_2022_111_MOESM8_ESM.pdf]

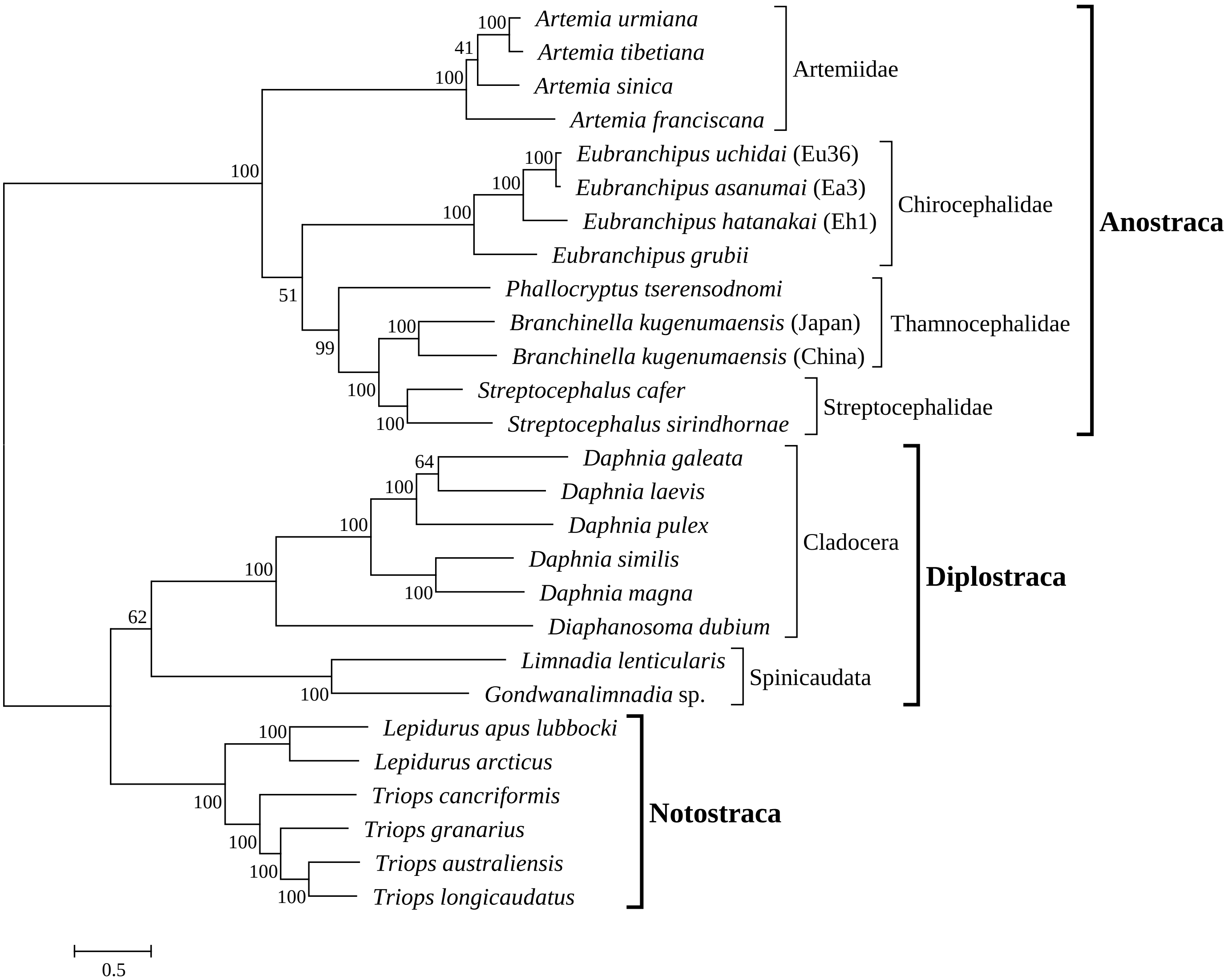

Supplementary Fig. S3 The maximum likelihood tree of Branchiopoda species based on the concatenated 13 protein-coding genes in the mitochondrial genome. A scale bar representing the number of nucleotide substitutions per site is shown. Values near the nodes represent the percent bootstrap probability.
