# Supplementary material for: Complete mitochondrial genomes of three fairy shrimps from snowmelt pools in Japan
Source: BMC Zool. 2022 Feb 9;7:11. doi: 10.1186/s40850-022-00111-2 (PMC10127424; doi:10.1186/s40850-022-00111-2)
Supplement: Supplementary file 9 — Additional file 9. [file 40850_2022_111_MOESM9_ESM.pdf]

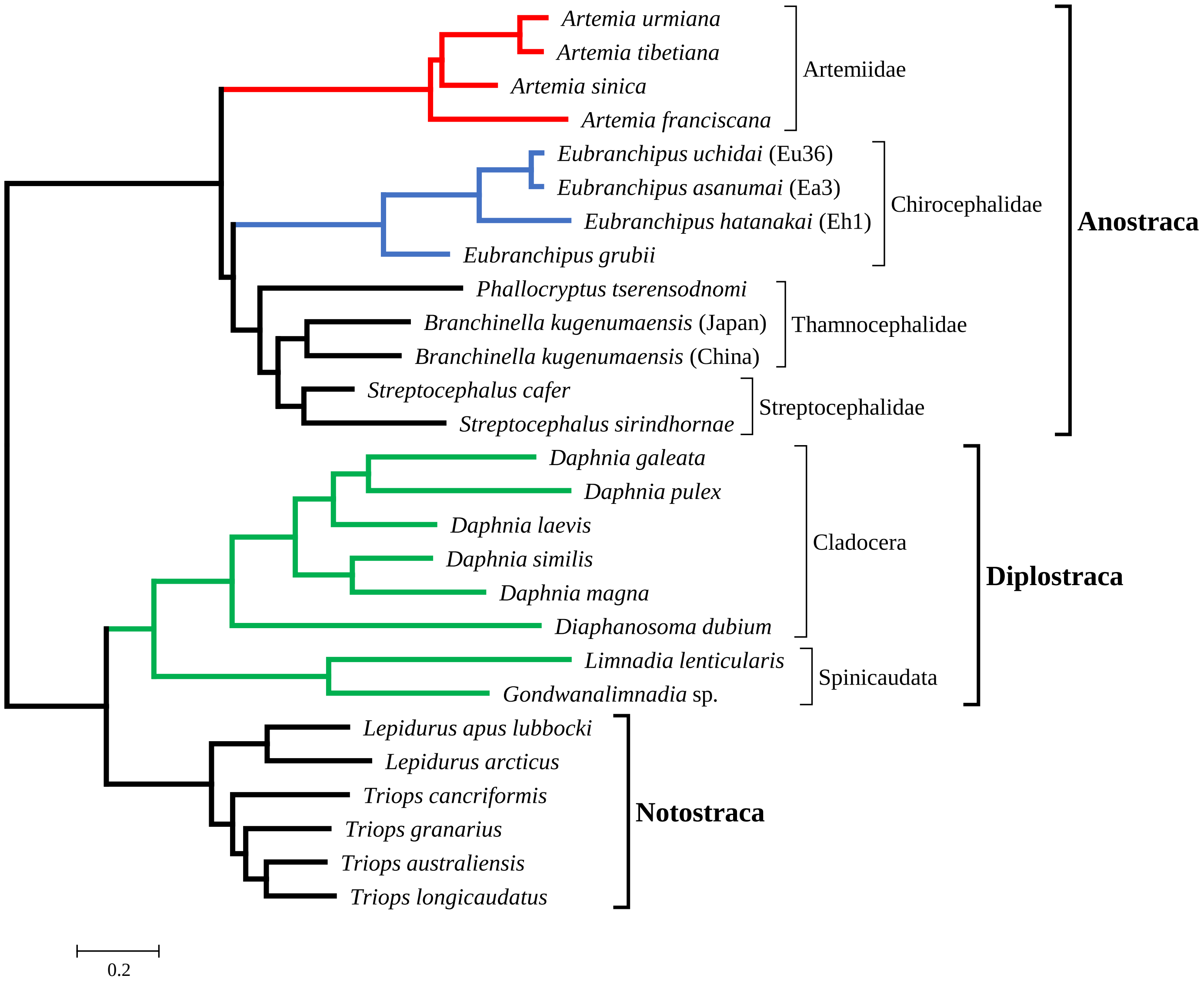

Supplementary Fig. S4 The neighbor-joining tree of Branchiopoda species based on the concatenated 13 protein-coding genes in the mitochondrial genome (the same as **Fig. 4**). A scale bar representing the number of nucleotide substitutions per site is shown. For substitution rate comparisons (**Table 4**), local clock models assumed that Artemiidae (#1, red branches), Chirocephalidae (#2, blue branches), and Diplostraca (#3, green branches) have higher substitution rates than Notostraca, Thamnocephalidae, and Streptocephalidae (black branches). Three rates (#1, #2, and #3) are assumed to be equal in the 'local clock1' model and to be independent in the 'local clock2' model.
